# Facile construction of dual‐response super‐resolution probes for tracking organelles dynamics

**DOI:** 10.1002/EXP.20230145

**Published:** 2024-03-12

**Authors:** Daili Liu, Guiqian Fang, Yanfeng Wang, Caicai Meng, Zhidong Liu, Qixin Chen, Xintian Shao

**Affiliations:** ^1^ School of Chinese Materia Medica Tianjin University of Traditional Chinese Medicine Tianjin China; ^2^ Institute of Materia Medica Science and Technology Innovation Center Shandong First Medical University and Shandong Academy of Medical Sciences Jinan Shandong China; ^3^ Department of Cancer Biology University of Cincinnati College of Medicine Cincinnati Ohio USA; ^4^ School of Life Sciences Science and Technology Innovation Center Shandong First Medical University and Shandong Academy of Medical Sciences Jinan Shandong China; ^5^ State Key Laboratory of Component‐based Chinese Medicine Tianjin University of Traditional Chinese Medicine Tianjin China; ^6^ Departments of Diagnostic Radiology Chemical and Biomolecular Engineering and Biomedical Engineering Yong Loo Lin School of Medicine and Faculty of Engineering National University of Singapore Singapore Singapore

**Keywords:** dual‐labeling, ferroptosis, lipid droplets, mitochondria, nanoscopic, toolbox

## Abstract

Super‐resolution imaging techniques, such as structured illumination microscopy (SIM), have enabled researchers to obtain nanoscale organelle‐level outputs in living systems, but they impose additional stringent requirements on fluorescence probes. However, high‐performance, custom‐designed SIM probes that can explain underlying biological processes remain unavailable. Herein, a customizable engineering toolkit is developed for the facile assembly of SIM probes suitable for subcellular component detection. This toolkit is used to customize a fluorescent molecule, **CPC** (coumarin–phenylhydrazine–carboxyl), capable of simultaneously monitoring peroxynitrite (ONOO^−^) and polarity distribution in mitochondria and lipid droplets (LDs), respectively, through functional ON–OFF mechanisms. The customized **CPC** molecule demonstrated excellent imaging capabilities under SIM, enabled the successful localization of multiple organelles, and reliably tracked the distribution of different components, thus facilitating the study of the interplay between organelles. Using **CPC**, the physical transition of intracellular LDs is demonstrated from heterogeneity to homogeneity. This was specifically observed during ferroptosis where the polarity of the LDs increased and their morphology became more contracted. Furthermore, the loss of LDs functionality could not counteract the accumulation of ONOO^−^ within the mitochondria, leading to the decoupling of mitochondrial LDs during ferroptosis. These results confirmed the potential mechanism of LDs dysfunction and decoupling triggered via cumulative mitochondrial oxidative stress during ferroptosis. To summarize, this toolkit will be a powerful tool for examining subtle variations among components during the interplay between different organelles, thus offering novel avenues for understanding and treating related diseases.

## Graphical abstract

**CPC** probe with dual response to LDs polarity and mitochondrial ONOO^−^ developed using a toolkit, which indicates an increase in homogeneity of lipid droplets and fibrous‐like mitochondria during ferroptosis that inhibits LDs–mitochondrial interactions.

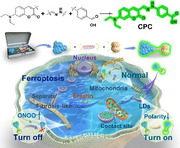

## 1 INTRODUCTION

Recent advances in super‐resolution fluorescence microscopy techniques, such as structured illumination microscopy (SIM),^[^ ^1^ ^]^ photoactivated localization microscopy,^[^ ^2^ ^]^ stochastic optical reconstruction microscopy,^[^ ^3^ ^]^ and stimulated emission depletion,^[^ ^4^ ^]^ have enabled the visualization of dynamic nano‐scale structures by surpassing the Abbe diffraction limit.^[^ ^5^ ^]^ The imaging characteristics of SIM and its excellent fluorescent probes, make it suitable for the dynamic tracking of live cells.^[^ ^5^ ^]^ The structured illumination projected above the sample using specific spatial frequencies or geometries provides achieving super‐resolution images that are observed beyond the diffraction limit.

Although SIM is extremely compatible with fluorescent probes, careful consideration of important design elements is crucial for achieving high‐quality imaging.^[^ ^6^ ^]^ These elements include: (1) small probe size to facilitate high‐density labeling for super‐resolution microscopy;^[^ ^1^, ^6^ ^]^ (2) high quantum yield for clear imaging under low‐light conditions and reduced exposure time;^[^ ^6^, ^7^ ^]^ (3) high photostability for long‐term dynamic imaging;^[^ ^1^, ^7^ ^]^ and (4) other factors such as narrow spectral characteristics and large Stokes shifts for multichannel imaging to enhance the signal‐to‐noise ratio.^[^ ^1c^ ^]^ Thus, iterative trial‐and‐error processes and associated time costs were performed to design probes that meet the abovementioned requirements. Fortunately, many small‐molecule fluorescent probes have recently been successfully developed, enabling applications such as multicolor imaging,^[^ ^1^, ^6^ ^]^ specific organelle targeting,^[^ ^8^ ^]^ the simultaneous detection of multiple analytes,^[^ ^6^, ^9^ ^]^ large Stokes shifts,^[^ ^1c^ ^]^ and high signal‐to‐noise ratios.^[^ ^1^, ^7^ ^]^ These probes have been utilized in SIM imaging and demonstrate novel biological processes, such as ferroptosis.^[^ ^10^ ^]^

Ferroptosis, a recently discovered regulated form of cell death, is caused by the imbalance of redox homeostasis, characterized by the accumulation of considerable amount of iron and lipid peroxide.^[^ ^10^, ^11^ ^]^ Conventional tools such as electron microscopy and conventional fluorescence microscopy capture information related to the morphological changes of lipid accumulation‐induced lipid droplets (LDs) and oxidative stress‐induced mitochondria.^[^ ^12^ ^]^ Although many aspects of morphological regulation are understood, the specific active molecular association changes in LDs/mitochondria during ferroptosis are still unclear, as electron microscopy can only report information related to fixed cells and traditional optical microscopes only provide cellular‐level data.^[^ ^12^, ^13^ ^]^ Thus, to examine specific crosstalk regulatory information on internal active molecules in LDs/mitochondria, SIM super‐resolution imaging utilizing different excellent fluorescent probes is a feasible visualization technique.

To address the aforementioned limitations, we have compiled the practical elements of a fluorescence probe, which have been confirmed in SIM by our group^[^ ^1^, ^6^, ^10^, ^14^ ^]^ and other research teams.^[^ ^8^, ^12^, ^15^ ^]^ Accordingly, we developed a customizable and user‐friendly multimolecular simultaneous response fluorescent probe toolbox that effectively detected the crosstalk information of subcellular multi‐active molecules. As a proof‐of‐concept, our toolkit included components responsive to polarity changes in order to monitor ionic changes in mitochondria and LDs during ferroptosis, facilitating the labeling of LDs and indicating mitochondrial status via reactive oxygen species. By combining these components, we designed fluorescent probes, such as the coumarin–phenylhydrazine–carboxyl (**CPC**)‐based dual‐labeling probe, to detect polarity and changes in peroxynitrite anion (ONOO^−^) levels for the simultaneous labeling of LDs and mitochondria, respectively. Using fluorescent probes along with SIM, we explained the multi‐molecule crosstalk interplay between mitochondria and LDs decoupling during ferroptosis. Moreover, this toolkit expedites the design and development process of fluorescent probes, by reducing the technical barriers for researchers, and fosters the advancement and application of fluorescent probe‐based technology.

## 2 RESULTS AND DISCUSSIONS

### 2.1 Rapid construction of dual‐targeting fluorescent probes using components from a toolkit

**FIGURE 1:**
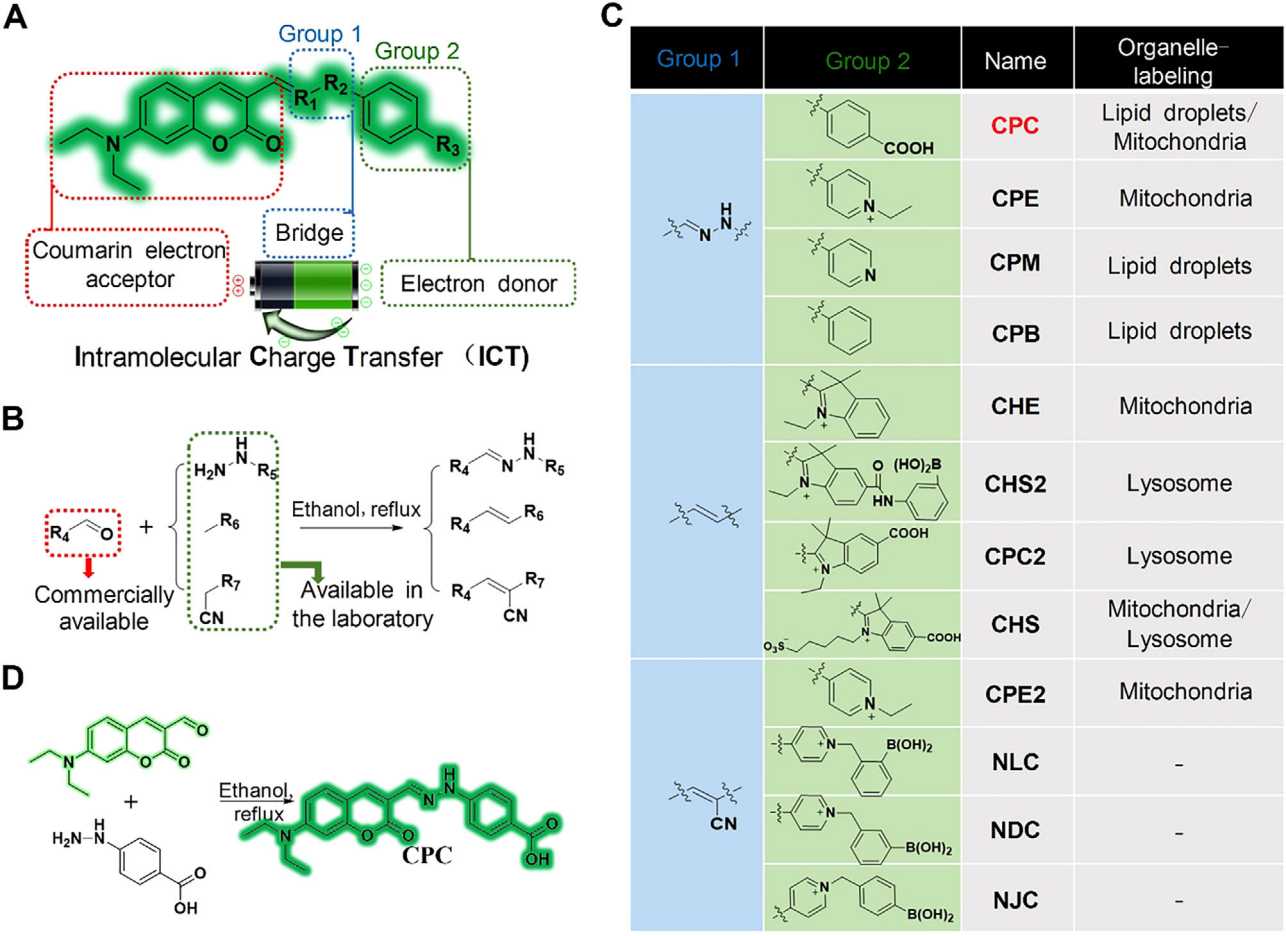
A toolkit platform for dual‐labeling probes. (A) Primary components of dual‐labeling toolkit's fluorescence response mechanism. (B) Universal synthesis engineering workflow of a dual‐labeling toolkit. (C) Replaceable elements and important components of a dual‐labeling toolkit. (D) Representative **CPC** optical molecules constructed using a dual‐labeling toolkit.

To obtain custom‐designed SIM probes, we used a toolkit with components containing the structural properties needed for labeling LDs and mitochondria.^[^ ^7^, ^8^, ^16^ ^]^ The LDs labeling portion of the probe included a fluorescence donor–π–acceptor (D–π–A) structure sensitive to changes in solvent polarity.^[^ ^16c^ ^]^ We selected the coumarin unit as the acceptor component owing to its excellent electron‐withdrawing properties and similarity to Nile Red, a commercial dye used for LDs.^[^ ^7^, ^16^, ^17^ ^]^ The mitochondrial labeling portion of the probe was optimized to respond to ONOO^−^, a reactive oxygen species (ROS) produced by mitochondrial metabolism using a C═N double bond, which readily reacts with ONOO^−^ and is commonly used for designing mitochondria‐labeling probes.^[^ ^6^, ^18^ ^]^ Our design used a coumarin component as an electron acceptor, an ROS‐responsive double bond (**Group 1**) as a π bridge, and in‐house intermediates (**Group 2**) as electron donors, thus resulting in a D–π–A configuration with an intramolecular charge transfer (ICT) ability. Moreover, **Group 1** and **2** were interchangeable elements used to develop different π bridges and electron donors, respectively (Figure 1A). The final step in assembling the dual probe involved a one‐step condensation reaction using commercially available aldehyde‐based coumarins and in‐house intermediates, thus providing a universal synthesis workflow for producing a series of probes (Figure 1B). Using a facile and systematic combinatorial approach with elements from **Group 1** and **2**, we explored a wide range of features and components (Figure 1C). The resulting probes were confirmed to image LDs, mitochondria, and lysosomes (Figures S1–S6). These efforts led us to identify a **CPC**‐based as our primary probe (Figure 1D). To summarize, our method for developing dual‐targeting probes allowed us to quickly discover a promising probe (**CPC**) to meet the stringent requirements of SIM probes, which we then confirmed and characterized.

### 2.2 Dual mode CPC response to polarity and ONOO^−^ in vitro

**FIGURE 2:**
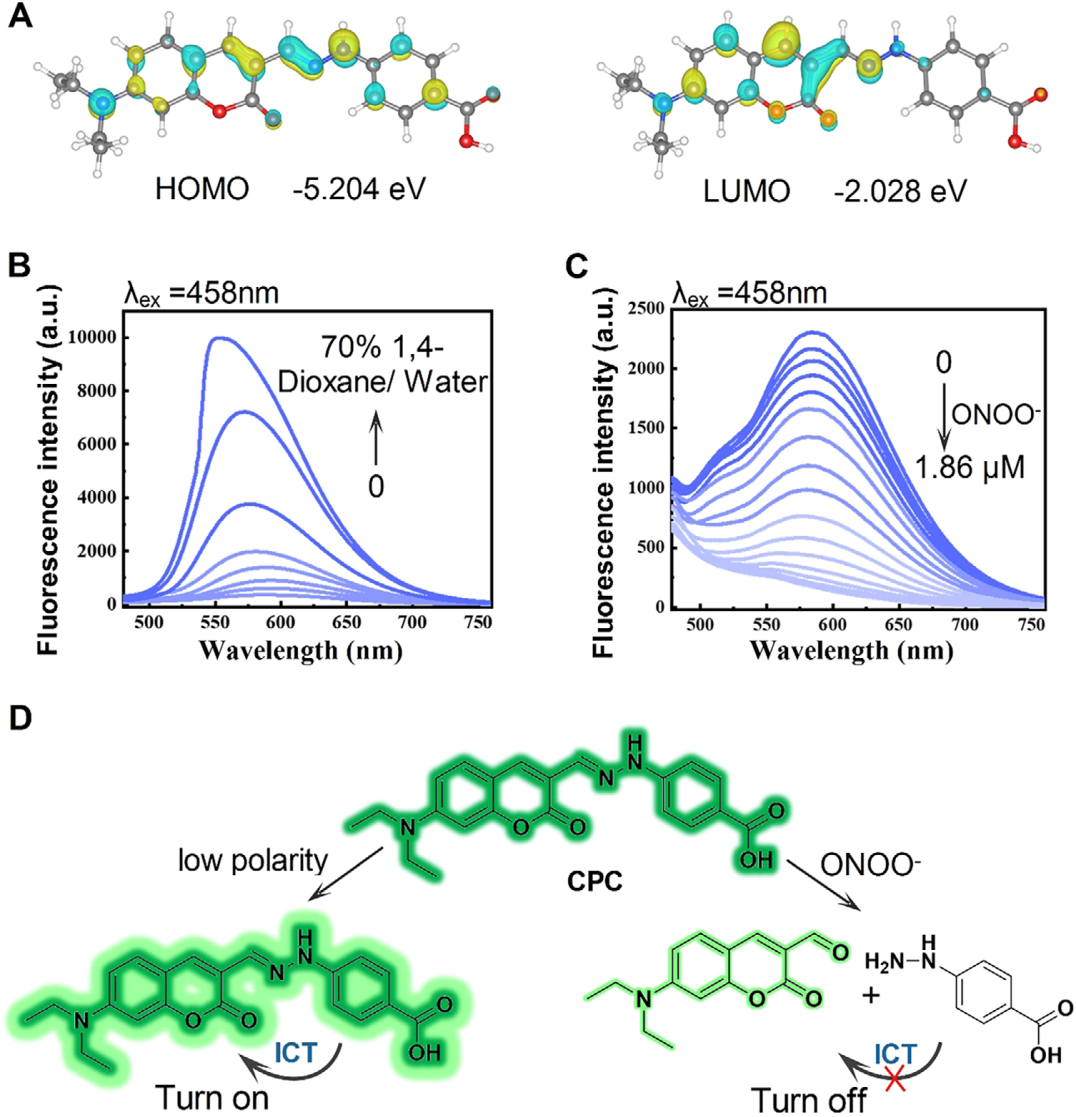
**CPC** optical turned on to respond polarity and optical turned off to respond ONOO^−^. (A) DFT calculation to confirm **CPC** fluorescence response mechanism. (B) **CPC** fluorescence emission spectra in 1,4‐dioxane and water mixtures of various proportions, room temperature, 10 min, *λ*_ex_ = 458 nm. (C) **CPC** fluorescence emission spectra in different ONOO^−^ concentrations in HEPSE (0.01 m), room temperature, 10 min, *λ*_ex _= 458 nm. (D) Schematic of dual **CPC** modes that “turn‐on” in response to polarity and “turn‐off” in response to ONOO^−^.

The presence of a classic ICT process in the coumarin‐based SIM probe was confirmed by density functional theory (DFT) calculations using Gaussian programs. The distribution of the highest occupied molecular orbital electrons was primarily concentrated on the carboxyphenylhydrazine unit, with some electrons distributed in the 7‐diethylamino functional group, while the lowest unoccupied molecular orbital electrons were was primarily dispersed on the aldehyde coumarin. Thus, the carboxyphenylhydrazine unit was considered as the main electron donor for the aldehyde coumarin, forming the original design of the proposed ICT‐based probe (Figure 2A). The optical properties of **CPC** were examined, thus revealing a substantial 124‐nm‐sized Stokes shift that was particularly advantageous in achieving a low signal‐to‐noise ratio (Figure S7).^[^ ^19^ ^]^ Moreover, **CPC** demonstrated a responsive fluorescence behavior toward polarity, with the intensity of the **CPC** emission gradually increasing with the 1,4‐dioxane content increased and exhibiting a blue shift (Figure 2B). The octanol/water partition coefficient (*P*_o/w_) of **CPC** was calculated as 0.482 (Figure S8 and Table S1). In summary, the large Stokes shift, strong fluorescence emissions in low‐polarity solvents, and appropriate lipophilicity of **CPC** were comparable to the properties of previously reported LD‐based probes.^[^ ^7^, ^16^ ^]^ Therefore, **CPC** can be used as a “turn‐on” probe that responds to the polarity of its surroundings. We further examined its reactivity with ONOO^−^ in vitro. The maximum absorbance wavelength of **CPC** was ≈458‐nm, and its intensity decreases when ONOO^−^ was introduced (Figure S9). An increase in the concentration of ONOO^−^ results in a reduction in its fluorescence intensity at 582‐nm (Figure 2C). These observations support the design of the probe, which incorporated a C═N double bond as the reactive element to ONOO^−^. Exposure to ONOO^−^ led to the disruption of the π bridge and a reduction in the fluorescence at 582‐nm. Based on these results, we determined that the limit of detection (LOD) was 123 nm (Figure S10). Thus, exposure to ONOO^−^ results in the disruption of the π‐bridge and fluorescence quenching at 582‐nm.

To confirm the mechanism of **CPC**–ONOO^−^ sensing, we analyzed a solution containing 0.1‐mm **CPC** and 0.1‐mm ONOO^−^ using high‐resolution mass spectrometry (HRMS). The reaction produced products with masses of 246.1188 and 268.1015 that respectively corresponded to the [C+H]^+^ of 246.1125 and [C+Na]^+^ of 268.0944 ion peaks (Figure S11).^[^ ^6^, ^18^ ^]^ To investigate potential interfering, we measured the fluorescence response of **CPC** to Co^2+^, H_2_O_2_, ^1^O_2_, NO, and d‐glucose and found that these responses were negligible (Figure S12). Moreover, **CPC** maintained a high level of fluorescence (>2000 a.u.) over a pH range of 6−12 (Figure S13), displayed a rapid response to ONOO^−^, and required only ≈10 min to quench the fluorescence (Figure S14). Moreover, **CPC** maintained a stable fluorescence intensity without the addition of ONOO^−^ for 30 min (Figure S15). Furthermore, fluorescence lifetime measurements confirmed that **CPC** as an excellent fluorescence probe (Figure S16). These results indicated that **CPC** was a stable probe with a fluorescence function that is “turned‐off” in response to changes in ONOO^−^ levels. To summarize, these results demonstrate the exceptional fluorescence properties of **CPC** in a laboratory setting using a fluorescence response to changes in polarity and ONOO^−^ levels (Figure 2D).

### 2.3 Dual‐mode imaging performance of CPC in living cells

**FIGURE 3:**
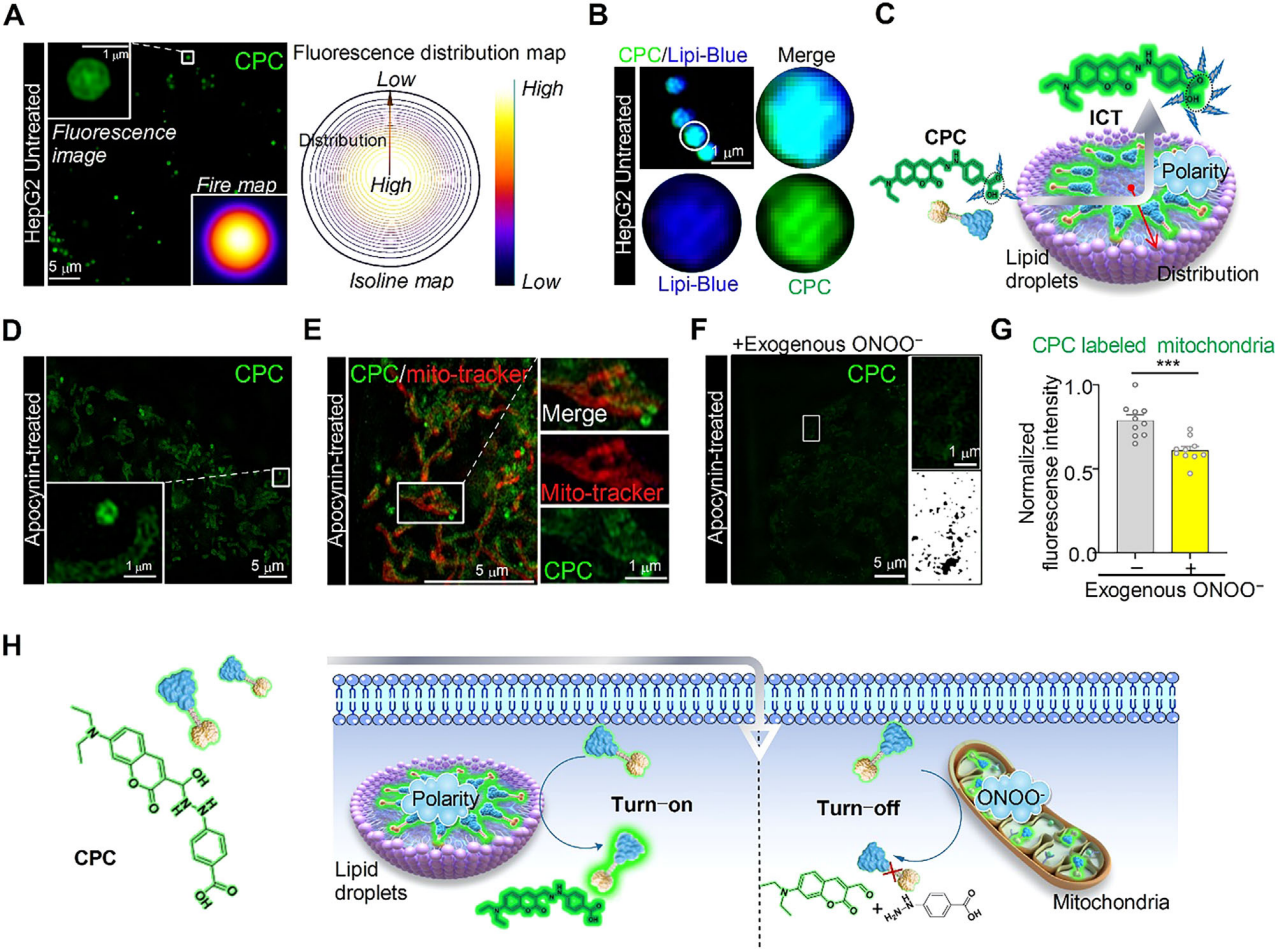
Dual‐mode **CPC** imaging responds to LDs polarity and mitochondrial ONOO^−^ generation in living cells. (A) SIM image of cells stained with **CPC** for 40 min (scale bar = 5 µm). (B) Merged SIM images of **CPC**‐ and Lipi‐Blue‐stained cells (scale bar = 5 µm). (C) Schematic diagram of **CPC** in LDs. (D) SIM image of **CPC** in apocynin‐inducted HepG2 cells (scale bar = 5 µm); enlarged images of regions in (D) (scale bar = 1 µm). (E) Merged SIM images of **CPC**‐ and PKMTDR‐stained apocynin‐inducted cells (scale bar = 5 µm); enlarged images of regions in (E) (scale bar = 1 µm). (F) SIM image of **CPC** in ONOO^−^‐treated apocynin‐inducted HepG2 cells (scale bar = 5 µm). (G) Normalized mean fluorescence intensity of **CPC** in untreated and ONOO^−^‐treated apocynin‐inducted HepG2 cells; data are mean ± SEM (*n *= 10 areas from three cells; ***p < 0.001). (H) Schematic diagram of dual‐mode **CPC** response to LDs polarity and mitochondrial ONOO^−^ generation in living cells. **CPC**: 10 µm, *λ*_ex _= 405 nm, *λ*_em _= 505−550 nm; Lipi‐Blue: 200 nm, *λ*_ex _= 405 nm, *λ*_em _= 417−476 nm; PKMTDR: 250 nm, *λ*_ex _= 640 nm, *λ*_em _= 655−705 nm.

The abovementioned experiments demonstrate that **CPC** meets the requirements of SIM probes. Thus, we used SIM to assess the responsiveness of **CPC** in living cells, a microscopy technique capable of achieving a spatial resolution of up to 100‐nm without the need for complex instruments or sample preparation.^[^ ^15c^ ^]^ Our observation of HepG2 cells incubated with **CPC** demonstrated the presence of many randomly distributed puncta emitting strong green fluorescence (Figure 3A). When HepG2 cells were co‐stained with **CPC** and the commercial LDs probe known as Lipi Blue,^[^ ^20^ ^]^ the **CPC**‐labeled puncta were reported in the inner region of Lipi Blue‐labeled LDs (Figure 3B). In determining the degree of **CPC** and Lipi Blue colocalization, we calculated a high Pearson colocalization coefficient (PCC) of 0.92, indicating that **CPC** was localized in the LDs (Figure S17).^[^ ^20^ ^]^ We discovered that **CPC** fluorescence was extremely responsive to low polarity, as shown in Figure 2B. Moreover, LDs have non‐polar components such as cholesteryl esters and triglycerides.^[^ ^16b,c^ ^]^ Consequently, LDs exhibited intense green fluorescence upon the entry of **CPC** into their low‐polarity internal microenvironment (Figure 3C). Confocal microscopy imaging supported the intense fluorescence result (Figure S18).

Apocynin, a reactive oxygen inhibitor, was introduced to inhibit the production of ONOO^−^ in living cells, as the fluorescence of **CPC** was quenched with ONOO^−^.^[^ ^21^ ^]^ SIM images revealed the fluorescent green particles and fibrous‐like mitochondria of **CPC** (Figure 3D). When induced with 1‐mM apocynin for 4 h (Figure 3E), the **CPC** considerably overlapped with a commercial mitochondrial probe (i.e., PK Mito‐Tracker Deep Red, PKMTDR),^[^ ^22^ ^]^ thus confirming its localization in mitochondria (Figure S19). To confirm the response of **CPC** to ONOO^−^ in mitochondria, a control assay was performed by adding exogenous ONOO^−^ (Figure 3F), which resulted in a considerable reduction in the green fluorescence intensity of **CPC**‐labeled mitochondria (Figure 3G). **CPC** demonstrated bright fluorescence and efficient uptake at concentrations of 0.1−20.0 µm (Figure S20). The fluorescence intensity was stronger at 37°C compared to at 4°C or with metabolic inhibitors (MI) treatment, thus indicating an energy‐dependent entry in the cells. The inhibition of endocytosis with NH_4_Cl^[^ ^6e^ ^]^ reduced the intracellular fluorescence intensity, demonstrating that **CPC** entered cells via an energy‐dependent endocytosis mechanism (Figure S21). These experiments demonstrated that **CPC** enters cells via an energy‐dependent endocytosis mechanism. **CPC** did not demonstrate any effect on cell viability at concentrations of up to 30.0 µm (Figure S22). In addition, **CPC** was co‐stained with commercially available nuclei dye (Hoechest) and lysosome dye (Lyso‐Tracker Blue, LTB), showing the specificity of labeled LDs (Figure S23). These results demonstrated that **CPC** had excellent targetability to LDs and generality in different cell lines such as H9C2 and HeLa cells (Figure S24).

As a group, we found the consensus that **CPC** can effectively label LDs and mitochondria in living cells as a SIM probe, thus demonstrating its low toxicity and excellent cellular permeability. Furthermore, **CPC** responded differently when LDs polarity was present (i.e., turn‐on) or when mitochondrial ONOO^−^ was generated (i.e., turn‐off). Therefore, **CPC** can serve as a fluorescent probe that can simultaneously label and examine the connection between LDs polarity and mitochondrially produced ONOO^−^ in ferroptosis (Figure 3H), in addition to other processes.

### 2.4 CPC homogeneity during ferroptosis

**FIGURE 4:**
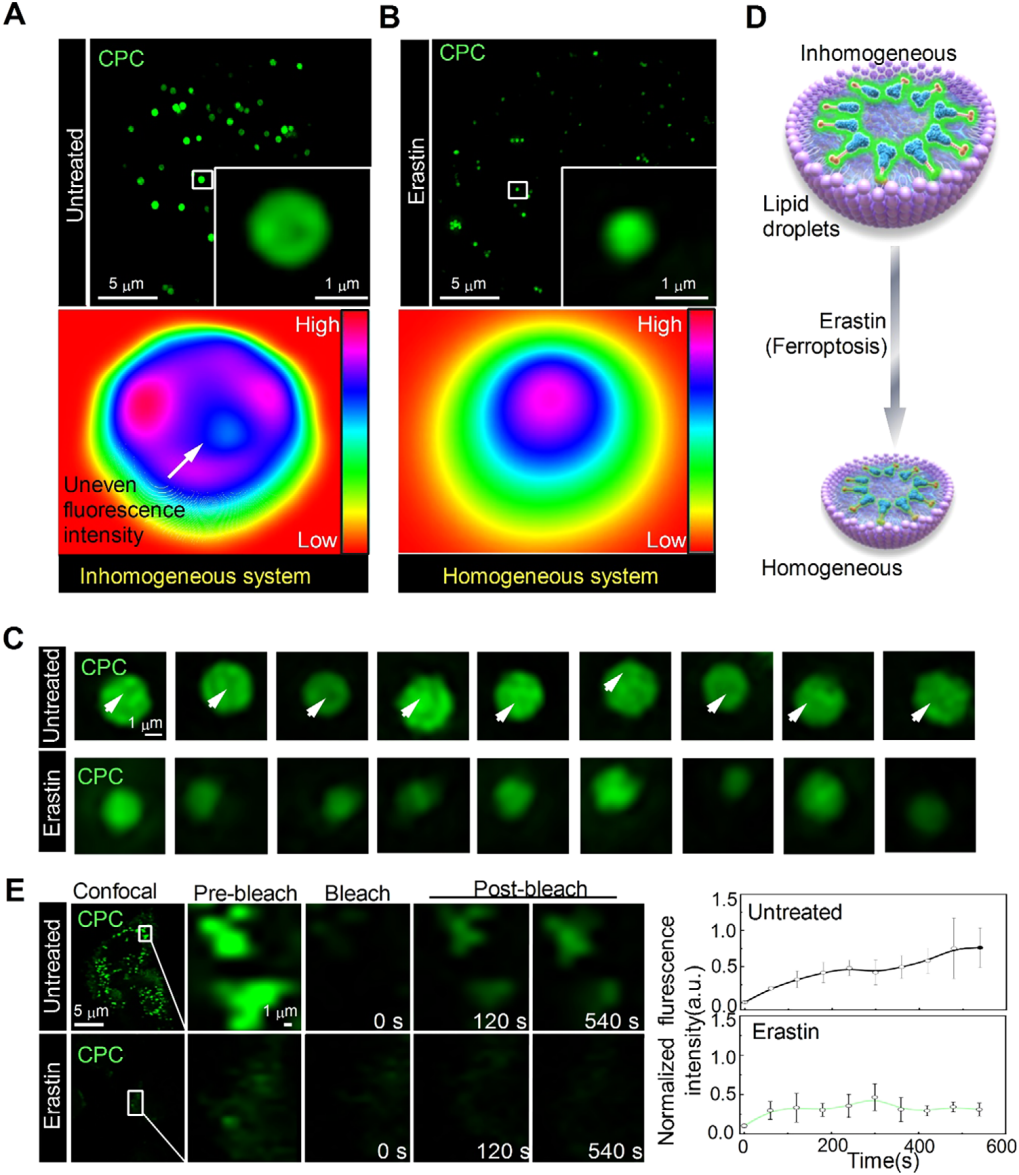
LDs homogeneity was formed during ferroptosis induction. (A) SIM images of **CPC**‐stained HepG2 cells (scale bar = 5 µm). (B) SIM images of **CPC**‐stained HepG2 cells after erastin treatment (scale bar = 5 µm). (C) Enlarged images of LDs in (A) and (B) (scale bar = 1 µm). (D) Schematic diagram of **CPC** in untreated and erastin‐treated LDs. (E) FRAP assay for untreated and erastin‐treated **CPC**‐stained LDs, normalized fluorescence‐recovery profile of LDs during fluorescence‐recovery process after erastin treatment or when LDs were left untreated; data are mean ± SEM (*n *= 3 measurements). **CPC**: 10 µm, *λ*_ex _= 405 nm, *λ*_em _= 505−550 nm.

The exceptional performance of the probe in detecting both mitochondrial ONOO^−^ and LDs polarity motivated us to examine changes in these parameters during ferroptosis. First, we utilized **CPC** probes to image and track LDs during ferroptosis. LDs are organelles responsible for storing lipids and include polar monolayers (e.g., phospholipids and cholesterol) and non‐polar contents (e.g., cholesteryl esters and triglycerides).^[^ ^7^, ^8^, ^16^ ^]^ During ferroptosis, there is an increase in polarity owing to lipid peroxidation, thus leading to the formation of a homogeneous system primarily composed of polarized material components. For this purpose, we examined untreated and erastin‐treated LDs stained with **CPC** (Figure 4A,B). The untreated group had an uneven distribution of fluorescence intensity, thus suggesting the inhomogeneous dispersion of LDs (Figure 4C). However, after erastin treatment, the uneven fluorescence intensity disappeared through the replacement and/or transformation of diverse components into components with relatively uniform polarity, thus resulting in no observed fluorescence collapse (Figure 4D). In addition, the full width at half maximum (FWHM) was calculated for the resolution of each image, including the untreated, erastin and apocynin groups, which were calculated as 98‐nm, 99‐nm and 124‐nm, respectively (Figure S25). These results indicated that the images were obtained at a high enough resolution to track LDs during ferroptosis. To confirm these results and assess the fluidity of the LDs, the fluorescence recovery after photobleaching (FRAP) experiment was determined.^[^ ^1a^ ^]^ Results demonstrated that **CPC** fluorescence recovery in normal LDs was faster compared to ferroptosis, indicating an increase in LDs density during ferroptosis (Figure 4E). Overall, ferroptosis affected the balance between the degradation and storage of LDs. Fatty acids were released in bioenergetic or anabolic reactions through the hydrolysis of triglycerides on the LDs surface.^[^ ^23^ ^]^ Our results showed that ferroptosis promoted this process and caused the excessive hydrolysis of triglycerides, illustrating an urgent need to reveal the mechanisms of LD dynamics during ferroptosis. Therefore, we propose that ferroptosis leads to an increase in the homogeneity of LDs and a loss of LDs function.

### 2.5 Mitochondrial ONOO^−^ levels and LDs polarity during ferroptosis

**FIGURE 5:**
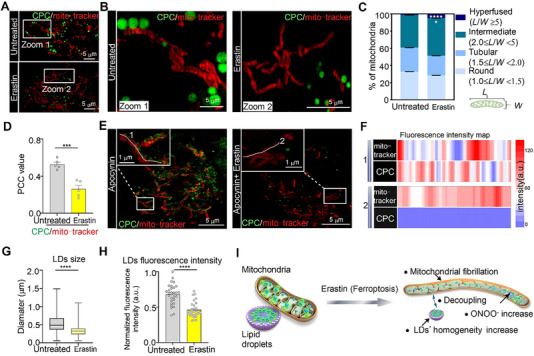
**CPC** revealed increased mitochondrial ONOO^−^ and improved LDs content polarity during ferroptosis. (A) Merged SIM images of untreated and erastin‐treated cells stained with **CPC** and mito‐tracker, PKMTDR after apocynin induction (scale bar = 5 µm). (B) Enlarged images of indicated regions in (A) (scale bar = 1 µm). (C) Quantitative analysis of untreated and erastin‐treated mitochondria; data are mean ± SEM (*n *= 3 cells; *p < 0.05, ****p < 0.0001). (D) Quantitative analysis of LDs and mitochondria co‐localization in untreated and erastin‐treated cells; data are mean ± SEM (*n *= 5 cells; ***p < 0.001). (E) Merged SIM images of untreated and erastin‐treated apocynin‐inducted cells stained with **CPC** and PKMTDR (scale bar = 5 µm); enlarged images of regions in (E) (scale bar = 1 µm). (F) Fluorescence intensity profiles along white line 1 in (E), fluorescence intensity profiles along white line 2 in (E). (G) Size distribution of untreated and erastin‐treated **CPC** puncta; data are mean ± SEM (*n *= 450 punctas from 6 cells). (H) Normalized mean fluorescence intensity of untreated and erastin‐treated LDs; data are mean ± SEM (*n *= 30 areas form 3 cells; ****p < 0.0001). (I) Schematic diagram of **CPC** reveals the changes of mitochondria and an increase in the homogeneity of LDs during ferroptosis. **CPC**: 10 µm, *λ*_ex _= 405 nm, *λ*_em _= 505−550 nm; PKMTDR: 250 nm, *λ*_ex _= 640 nm, *λ*_em _= 655−705 nm.

**CPC** was further used to reveal the relationship between mitochondrial ONOO^−^ levels and LDs polarity during ferroptosis. To determine changes in mitochondrial ONOO^−^ levels, HepG2 cells were treated with 20.0 µg mL^‐1^ of erastin for 12 h to induce ferroptosis. Then, the cells were stained with **CPC** and a mitochondria tracker (i.e., PKMTDR) (Figure 5A). Considerable differences in the morphology and fluorescence intensity of LDs and mitochondria were observed between their normal and corresponding states during ferroptosis (Figure 5B). Consistent with previous studies, we observed an increase in the presence of hyperfused mitochondria that resembled fibrosis‐like mitochondria (Figure 5C).^[^ ^14^, ^24^ ^]^ Moreover, the PCC of LDs and mitochondria decreased from 0.53 to 0.26 (Figure 5D), indicating that erastin promoted the uncoupling of LDs from mitochondria. Alternatively, owing to the “turn‐off” mechanism of **CPC**, erastin treatment may have led to higher levels of ONOO^−^, which in turn reduced the fluorescence at the mitochondria.

To examine this matter, a control experiment was performed where we inhibited the production of ROS in HepG2 cells treated with apocynin and stained with **CPC** and PKMTDR. These cells were either left untreated or treated with erastin (Figure 5E). The green fluorescence from the **CPC**‐labeled mitochondria was still present, but it diminished after erastin inducted ROS production (Figure 5F). This suggested that the presence of ONOO^−^, which is generated by erastin, deactivated visible **CPC** at the mitochondria, leading to ferroptosis. After erastin treatment, the average diameter of the LDs decreased from 0.52 to 0.32 µm, and the intensity of green fluorescence in the **CPC**‐labeled LDs was considerably reduced (Figure 5G,H). These results indicated that erastin treatment reduced the size of the LDs and an increase of LDs polarity. Furthermore, LDs and mitochondria were possibly become disconnected to some extent.

Although several probes for LDs have been reported, their ability to reveal the changes in LDs and other organelles is still lacking.^[^ ^25^ ^]^ Due to limitations in microscope imaging resolution and single organelle labeling, these probes only focus on studying changes in the fluorescence intensity of LDs. In contrast, the probe we reported could simultaneously label LDs and mitochondria. Furthermore, SIM imaging revealed the uncoupling of LDs and mitochondria during ferroptosis, indicating that ferroptosis caused changes in LD polarity and the upregulation of mitochondrial ONOO^−^. To summarize, our observations demonstrated changes in the morphology of the mitochondria as well as the increased homogeneity of LDs (i.e., changes in size and polarity) during ferroptosis (Figure 5I).

## 3 CONCLUSIONS

Based on a customizable engineering toolkit for the facile assembly of SIM probes, we developed a dual‐response probe (**CPC**) for the simultaneous monitoring of ONOO^−^ in mitochondria and polarity distribution in LDs. The toolkit resolved the stringent requirements of SIM for probes and provided a custom‐designed probe for tracking the interactions of multiple organelles. Initially, we utilized **CPC** to track changes during ferroptosis but found that it could also monitor various biological processes. Consequently, **CPC** proved to be an exceptional tool for observing changes in ONOO^−^ levels within mitochondria and gauging the polarity of LDs. Our investigation revealed that ferroptosis leads to the formation of fibrous‐like mitochondria and enhances the homogeneity of LDs. Moreover, we proposed that ferroptosis disrupts the interactions between LDs and mitochondria, although further visualization and examination are required to understand this process. **CPC** is thus a reliable dual‐targeting probe for the evaluation of cellular processes that impacts LDs and the production of mitochondrial ROS. Notably, **CPC** labeling offers a simplified single‐step process, thus reducing the complications associated with multi‐step labeling procedures. The design and development process of **CPC** will foster the advancement of fluorescent probe technology and provide additional comprehensive insights into the micro‐dynamics of different organelles during ferroptosis and other biological processes. This enhanced understanding of cellular functions will accelerate advancements in basic research and facilitate drug discovery and development.

## 4 EXPERIMENTAL SECTION

Experimental details are provided in the Supporting Information.

## AUTHOR CONTRIBUTIONS

Daili Liu and Guiqian Fang collected all 3D‐SIM super‐resolution microscopy data. Daili Liu analyzed and processed the SIM data. Guiqian Fang synthesized and characterized **CPC**. Daili Liu; Caicai Meng and Yanfeng Wang performed confocal laser scanning microscopy. Xintian Shao; Zhidong Liu and Qixin Chen conceived the project; designed the experiments; and wrote the manuscript with the help of all authors.

## CONFLICT OF INTEREST STATEMENT

The authors declare no conflicts of interest.

## Supporting information

Supporting Information

## Data Availability

This statement indicates that all the data pertaining to the study can be found in the article and the Supporting Information section. It further states that any additional data related to this work can be obtained by contacting the corresponding authors.
