# Supplementary material for: Facile construction of dual‐response super‐resolution probes for tracking organelles dynamics
Source: Exploration (Beijing). 2024 Mar 12;4(5):20230145. doi: 10.1002/EXP.20230145 (PMC11491301; doi:10.1002/EXP.20230145)
Supplement: Supplementary file 1 — Supporting Information [file EXP2-4-20230145-s001.docx]

Supporting Information

Facile Construction of Dual-response Super-resolution Probe for Tracking Organelles Dynamics

Daili Liu^1,2#^, Guiqian Fang^2,5#^, Yanfeng Wang^2^, Caicai Meng^3^, Zhidong Liu^1^*, Qixin Chen^2,4^*, Xintian Shao^3^*

^1^Institute of Traditional Chinese Medicine, Tianjin University of Traditional Chinese Medicine, Tianjin 301617, China.

^2^Institute of Materia Medica, Science and Technology Innovation Center, Shandong First Medical University & Shandong Academy of Medical Sciences, Jinan 250062, Shandong, China.

^3^School of Life Sciences, Science and Technology Innovation Center, Shandong First Medical University & Shandong Academy of Medical Sciences, Jinan 250062, Shandong, China.

^4^Departments of Diagnostic Radiology, Chemical and Biomolecular Engineering, and Biomedical Engineering, Yong Loo Lin School of Medicine and Faculty of Engineering, National University of Singapore, Singapore 119074, Singapore.

^5^Department of Cancer Biology, University of Cincinnati College of Medicine, Cincinnati, OH 45267, USA.

E-mail: lonerliuzd@163.com (Z. Liu); dnrv31@nus.edu.sg (Q. Chen); [shaoxintian@sdfmu.edu.cn (X. Shao)](mailto:shaoxintian@sdfmu.edu.cn%20(X.%20Shao);)

# D. Liu and G. Fang contributed equally to this work.

**Materials and methods**

**General materials**

7-(DiethylaMino)-2-oxo-2H-chroMene-3-carbaldehyde, 4-hydrazinobenzoic acid, 4-hydrazinylpyridine, phenylhydrazine, 4-aminoyphenylboronic acid, 1,4-butanesultone, 1,2-dichlorobenzene and other reagents for synthesis are analytically pure or chemically pure without further purification. These reagents for synthesis were purchased from Macklin or Aladdin company. In addition, fetal bovine serum (FBS) was obtained from VivaCell Shanghai (Shanghai, China). Dulbecco’s modified Eagle’s medium (#11965118, DMEM), penicillin-streptomycin (#15140163, 10,000 units/ml), Trypsin-EDTA (#25200-072), phenol-free medium (#1894117, Gibco, Grand Island, NY, USA) and other reagents for cell culture were obtained from Gibco BRL (Grand Island, NY, USA). Erastin, PK Mito-Tracker Deep Red (PKMTDR) and Lipi-Blue were obtained from Invitrogen (Eugene, Oregon, USA). Culture bottle was purchased from Guanzhou Jet Bio-Filtration Co., Ltd. HepG2 cells were gifted from Fengshan Wang’s lab (Shandong University).

**Synthesis and characterization**

All the probes were prepared using a one-step condensation reaction. Synthesis of **CPC**: a mixture of 7-(diethylamino)-2-oxo-2H-chromene-3-carbaldehyde (0.23 g, 0.94 mmol) and 4-hydrazinobenzoic acid (0.143 g, 0.94 mmol) were placed in a 50 mL flask with 10 mL dry ethanol for 3 h. The brown precipitate that formed was filtered, rinsed with ethyl acetate and dried to give 0.25 g (0.65 mmol, 69 %) of **CPC** as a brown solid. ^1^H NMR (600 MHz, DMSO-*d_6_*) *δ* (ppm) (Figure S26): 12.26 (s, 1H), 10.92 (s, 1H), 8.31 (s, 1H), 8.00 (s, 1H), 7.82 (d, *J* = 8.8 Hz, 2H), 7.59 (d, *J* = 8.9 Hz, 1H), 7.11 (d, *J* = 8.6 Hz, 2H), 6.74 (dd, *J* = 8.9, 2.3 Hz, 1H), 6.56 (d, *J* = 2.2 Hz, 1H), 3.45 (q, *J* = 7.0 Hz, 4H), 1.14 (t, *J* = 7.0 Hz, 6H). ^13^C NMR (151 MHz, DMSO-*d_6_*) *δ* (ppm) (Figure S27): 167.71, 161.28, 156.32, 151.12, 149.05, 136.54, 134.02, 131.61, 130.57, 120.90, 114.48, 111.68, 110.07, 108.89, 97.01, 44.64, 12.85. HRMS (Figure S28): For [M+H]^+^ m/z 380.1610. Found: [M+H]^+^ m/z 379.4800.

Synthesis and characterization of other probes are detailed in Supporting Information (Figure S29~72).

**Cytotoxicity assay**

The cytotoxicity assay was determined using a MTT assay. HepG2 cells were seeded in 96-well plates at a density of 5×10^3^ cells/well in DMEM with 10 % FBS and placed in an incubator (5 % CO_2_, 37 °C) for 24 h. The medium was replaced with 100 μL fresh medium containing different concentrations (0.1 μM, 1.0 μM, 5.0 μM, 10.0 μM, 20.0 μM and 30.0 μM) of **CPC**. After 12 h of incubation, 10 μL of MTT solution was added to each well, followed by incubation for 4 h under the same conditions. Then the medium was removed and added to 150 μL DMSO. After shaking 10 min, the absorbance of each well at 570 nm was determined by enzyme-linked immunosorbent assay. Cell viability (%) was calculated by the mean of absorbance value of treatment group divided by the mean of absorbance value of the control group.^[1-3]^

**Cell culture and imaging under OMX 3D-SIM or confocal laser scanning microscopy**

HepG2 cells were seeded at a density of 1×10^5^ on 35 mm glass-bottom culture dishes and incubated with 2 mL of DMEM with 10 % FBS for 24 h incubation (5 % CO_2_, 37 °C). HepG2 cells were incubated with 10.0 μM **CPC** for 40 min, and then were washed with fresh DMEM seven times. Finally, HepG2 cells were cultured in 1 mL of phenol-free medium and imaged under an OMX 3D-SIM extended-resolution microscope (DeltaVision, Inc) equipped with a 60×/1.42 numerical aperture oil-immersion objective lens and solid-state lasers.^[4]^ **CPC** was excited at 405 nm and emitted at 500-550 nm and cell images were obtained at 512 × 512 using Z-stacks with a step size of 0.125 μm. In addition, samples also were analyzed under a laser scanning microscope Nikon A1R (Nikon, Japan) equipped with 63×/1.4 numerical aperture oil immersion objective lens.

**Flow cytometry analysis**

HepG2 cells were seeded in 6-well plates at a density of 5×10^3^ cells/well in DMEM with 10 % FBS and placed in an incubator (5 % CO_2_, 37 °C) for 24 h. Then the medium culture was replaced with 1000 μL fresh medium containing different concentrations (0 μM, 0.1 μM, 1.0 μM, 10.0 μM, and 20.0 μM) of **CPC**. After being stained with **CPC** for 40 min, the cells were digested by trypsin and washed twice with precooled PBS. The cells were resuspended with 500 μL buffer and then detected by flow cytometry.

**Co-localization experiments**

HepG2 cells were seeded at a density of 1×10^5^ on 35 mm glass-bottom culture dishes and incubated with 2 mL of DMEM with 10 % FBS for 24 h incubation (5 % CO_2_, 37 °C). HepG2 cells were incubated with 10 μM **CPC** for 40 min, 100 nM PKMTDR for 30 min and 100 nmol/L Lipi-Blue for 30 min. Then HepG2 cells were washed with fresh DMEM seven times and incubated with 10.0 μM **CPC** for 40 min. Finally, HepG2 cells were cultured in 1 mL of phenol-free medium and imaged under an OMX 3D-SIM. PKMTDR was excited at 640 nm and monitored at 655–705 nm, Lipi-Blue was excited at 405 nm and monitored at 417–476 nm. The images were processed and analyzed using ImageJ.

**Data analysis**

Statistical analysis was performed using Origin 2018, GraphPad Prism 8 and ImageJ. Normality test is used to check the normal distribution. Data are presented as mean ± standard error of mean (SEM). SEM was used to compare experimental results with controls. In the case of non-normal distribution, the statistical comparison of results was test with a Mann-Whitney test, with levels of significance set at n.s. (no significant difference), ^*^*P*<0.05, ^**^*P*<0.01, ^***^*P* < 0.001 and ^****^*P* < 0.0001. Statistical significances and sample sizes in all graphs are indicated in the corresponding figure legends. DFT was calculated by Shiyanjia Lab ([www.shiyanjia.com](http://www.shiyanjia.com)).


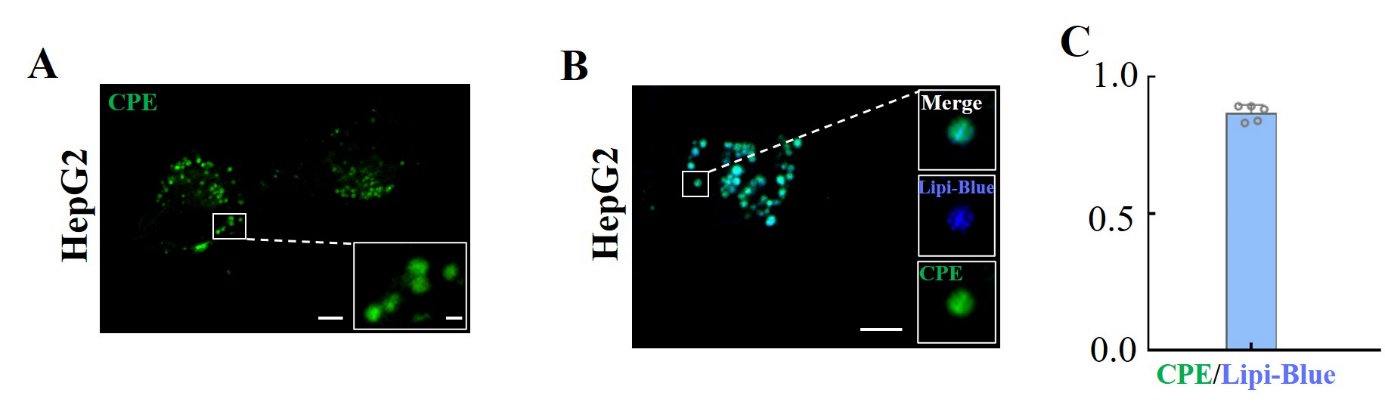


**FIGURE** S1 Microscopic images of HepG2 cells stained by CPE. (A) Confocal microscopic images of cells stained with **CPE** for 40 min (scale bar = 5 μm), enlarged images of regions in (A) (scale bar = 1 μm). (B) Merged confocal microscopic images of **CPE**‑ and Lipi‑Blue‑stained cells (scale bar = 5 μm); enlarged images of regions in (B) (scale bar = 1 μm). (C) Quantitative analysis of CPE and Lipi‑Blue co-localization; data are mean ±SEM (n =5). **CPE**: 10.0 μM, λ_ex_ = 488 nm, λ_em_ = 500–550 nm; Lipi‑Blue: 200 nM, λ_ex_ = 405 nm, λ_em_ = 417–476 nm.


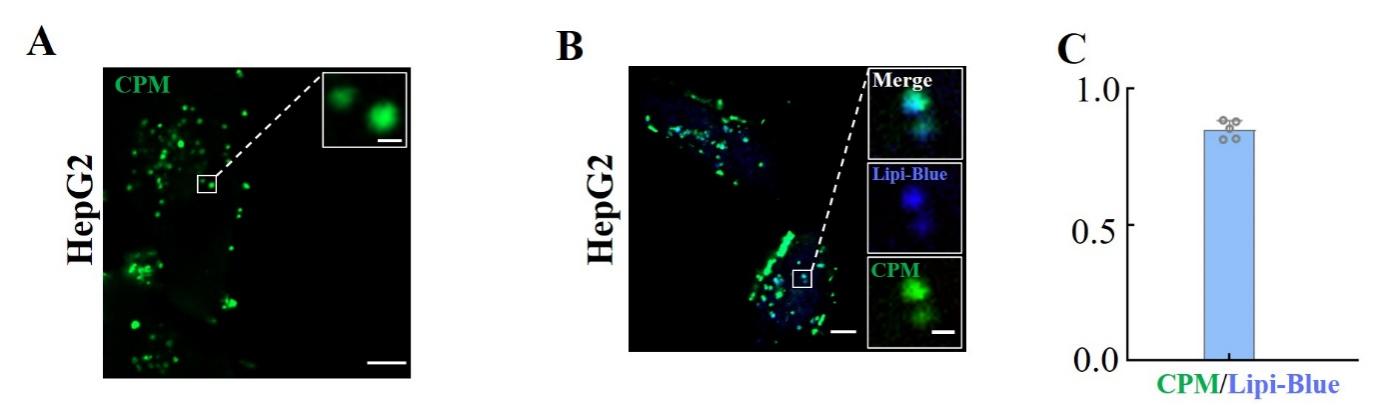


FIGURE S2 Microscopic images of HepG2 cells stained by CPM. (A) Confocal microscopic images of cells stained with **CPM** for 40 min (scale bar = 5 μm), enlarged images of regions in (A) (scale bar = 1 μm). (B) Merged confocal microscopic images of **CPM**‑ and Lipi‑Blue‑stained cells (scale bar = 5 μm); enlarged images of regions in (B) (scale bar = 1 μm). (C) Quantitative analysis of CPM and Lipi‑Blue co-localization; data are mean ±SEM (n =5). **CPM**: 10.0 μM, λ_ex_ = 488 nm, λ_em_ = 500–550 nm; Lipi‑Blue: 200 nM, λ_ex_ = 405 nm, λ_em_ = 417–476 nm.


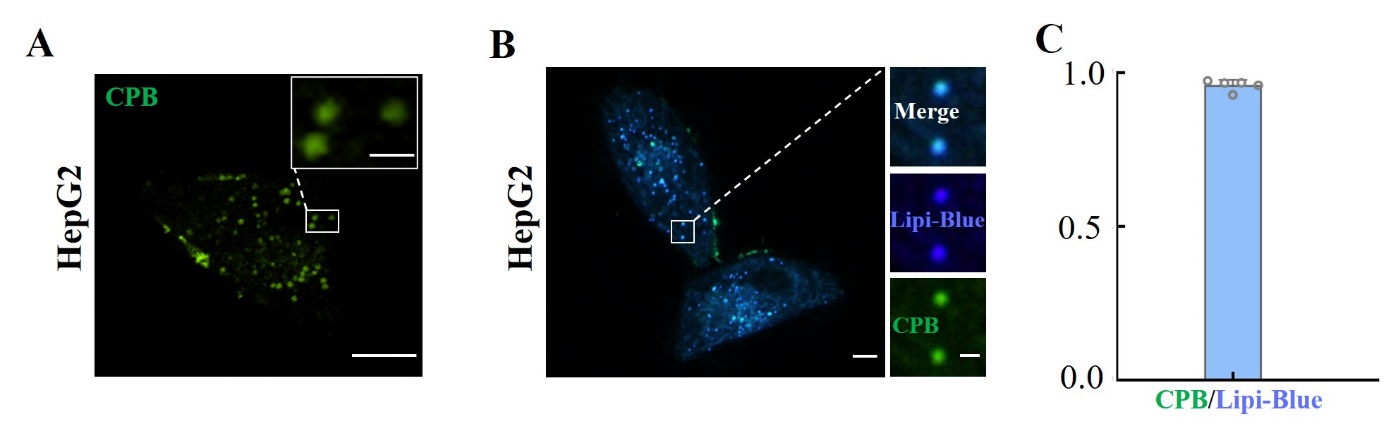


FIGURE S3 Microscopic images of HepG2 cells stained by CPB. (A) Confocal microscopic images of cells stained with **CPB** for 40 min (scale bar = 5 μm), enlarged images of regions in (A) (scale bar = 1 μm). (B) Merged confocal microscopic images of **CPB**‑ and Lipi‑Blue‑stained cells (scale bar = 5 μm); enlarged images of regions in (B) (scale bar = 1 μm). (C) Quantitative analysis of CPB and Lipi‑Blue co-localization; data are mean ±SEM (n =5). **CPB**: 10.0 μM, λ_ex_ = 488 nm, λ_em_ = 500–550 nm; Lipi‑Blue: 200 nM, λ_ex_ = 405 nm, λ_em_ = 417–476 nm.


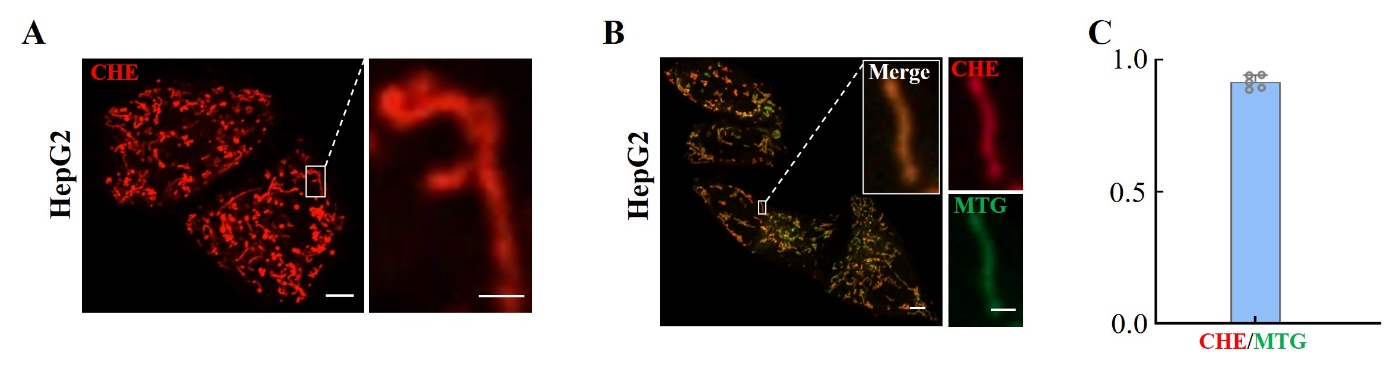


FIGURE S4 Microscopic images of HepG2 cells stained by CHE. (A) Confocal microscopic images of cells stained with **CHE** for 40 min (scale bar = 5 μm), enlarged images of regions in (A) (scale bar = 1 μm). (B) Merged confocal microscopic images of **CHE**‑ and MTG‑stained cells (scale bar = 5 μm); enlarged images of regions in (B) (scale bar = 1 μm). (C) Quantitative analysis of CPE and MTG co-localization; data are mean ±SEM (n =5). **CPE**: 5.0 μM, λ_ex_ = 561 nm, λ_em_ = 580–627 nm; MTG: 200 nM, λ_ex_ = 488 nm, λ_em_ = 500–550 nm.


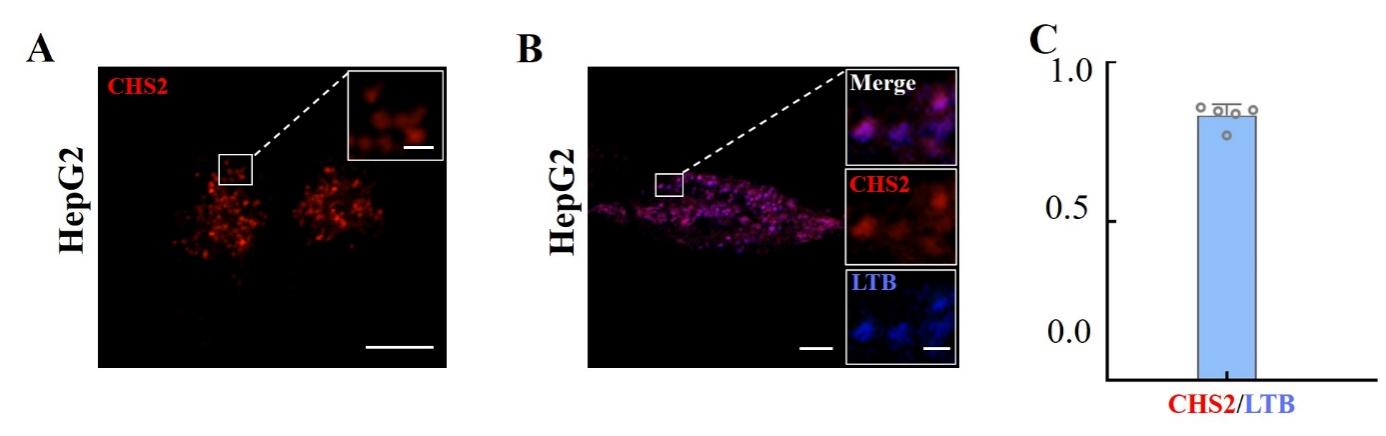


FIGURE S5 Microscopic images of HepG2 cells stained by CHS2. (A) Confocal microscopic images of cells stained with **CHS2** for 40 min (scale bar = 5 μm), enlarged images of regions in (A) (scale bar = 1 μm). (B) Merged confocal microscopic images of **CHS2**‑ and LTB‑stained cells (scale bar = 5 μm); enlarged images of regions in (B) (scale bar = 1 μm). (C) Quantitative analysis of CHS2 and LTB co-localization; data are mean ±SEM (n =5). CHS2: 10.0 μM, λ_ex_ = 405 nm, λ_em_ = 505–550 nm; LTB: 200 nM, λ_ex_ = 405 nm, λ_em_ = 417–476 nm.


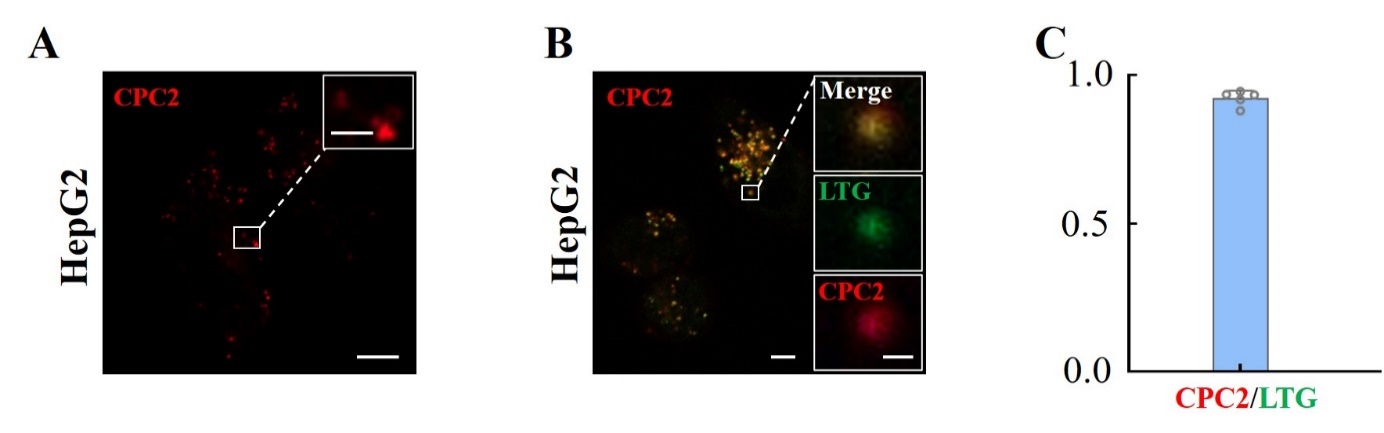


FIGURE S6 Microscopic images of HepG2 cells stained by CPC2. (A) Confocal microscopic images of cells stained with **CPC2** for 40 min (scale bar = 5 μm), enlarged images of regions in (A) (scale bar = 1 μm). (B) Merged confocal microscopic images of **CPC2**‑ and LTG‑stained cells (scale bar = 5 μm); enlarged images of regions in (B) (scale bar = 1 μm). (C) Quantitative analysis of CPC2 and LTG co-localization; data are mean ±SEM (n =5). **CPCS**: 10.0 μM, λ_ex_ = 405 nm, λ_em_ = 505–550 nm; LTG: 200 nM, λ_ex_ = 488 nm, λ_em_ = 500–550 nm.


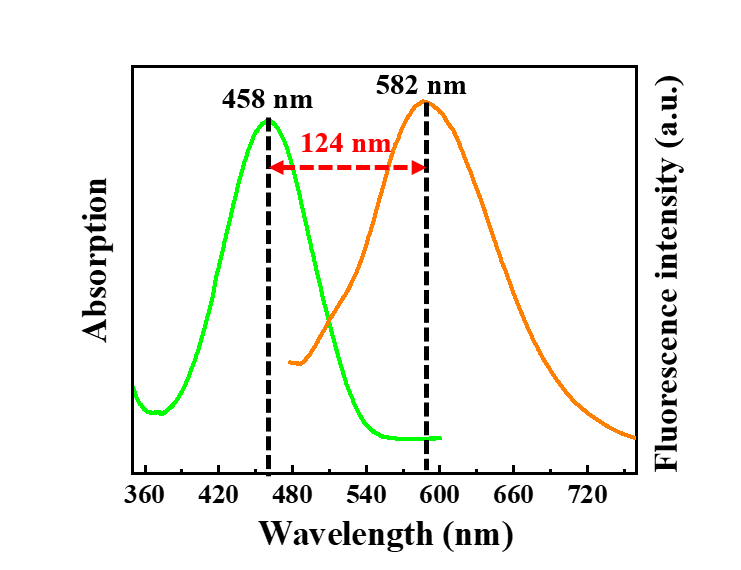


FIGURE S7 UV–vis absorption and fluorescence spectra of CPC (10.0 μM) in HEPES (0.01 M) at room temperature.


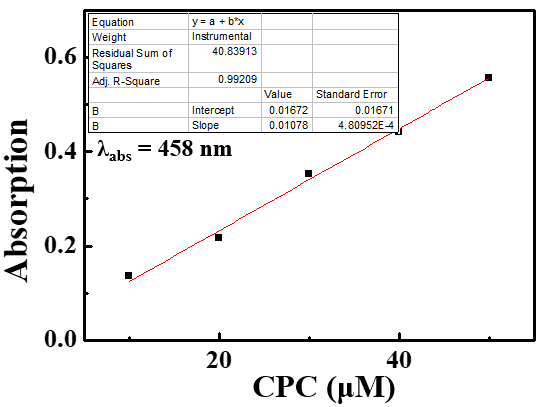


FIGURE S8 The standard curve (Y=0.01078X+0.01672) was determined using different concentrations of CPC (X) in octanol and its enhanced absorption intensity (Y).


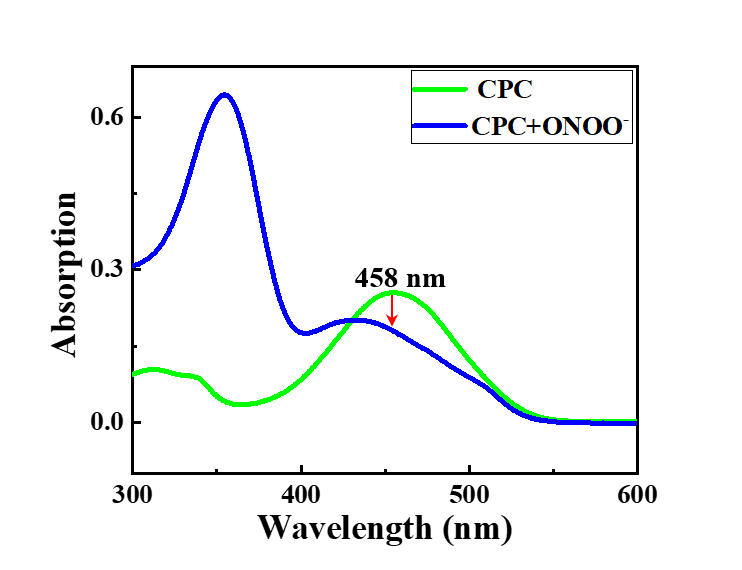


FIGURE S9 UV-vis absorption spectra of CPC (10.0 μM) without/with ONOO^-^ (10.0 μM) in HEPES (0.01 M), at room temperature.


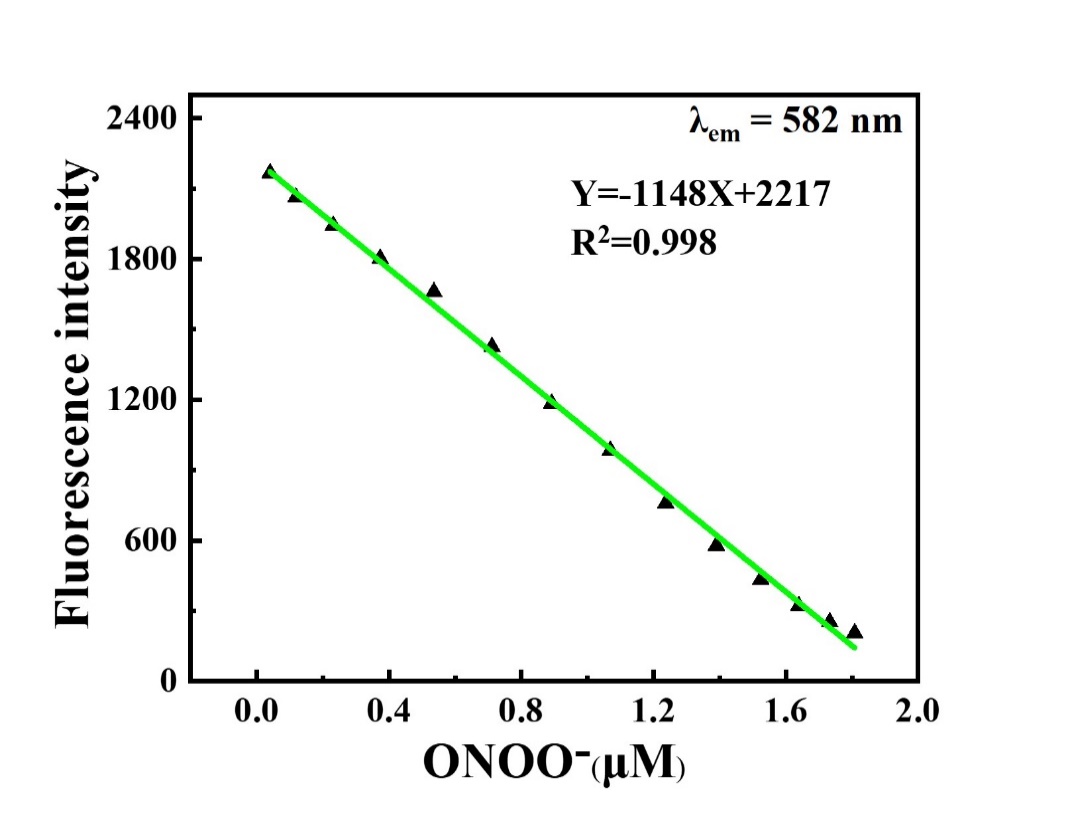


FIGURE S10 The standard curve of CPC with ONOO^-^ and limit of detection (LOD) was calculated using the equation LOD = 3*δ*/*S*, where *δ* is the standard deviation of the 8-time blank CPC signal and *S* is the calibration curve slope.

Linear Equation:

*Y*=-1148*X*+2217

*R^2^* =0.998

*S*=1147687630

*𝛿* =$\sqrt{\frac{\Sigma\left( F_{i}-F_{0} \right)^{2}}{N-1}}$ =47 (N=5) K=3

LOD =K × *δ*/*S*=123 nM


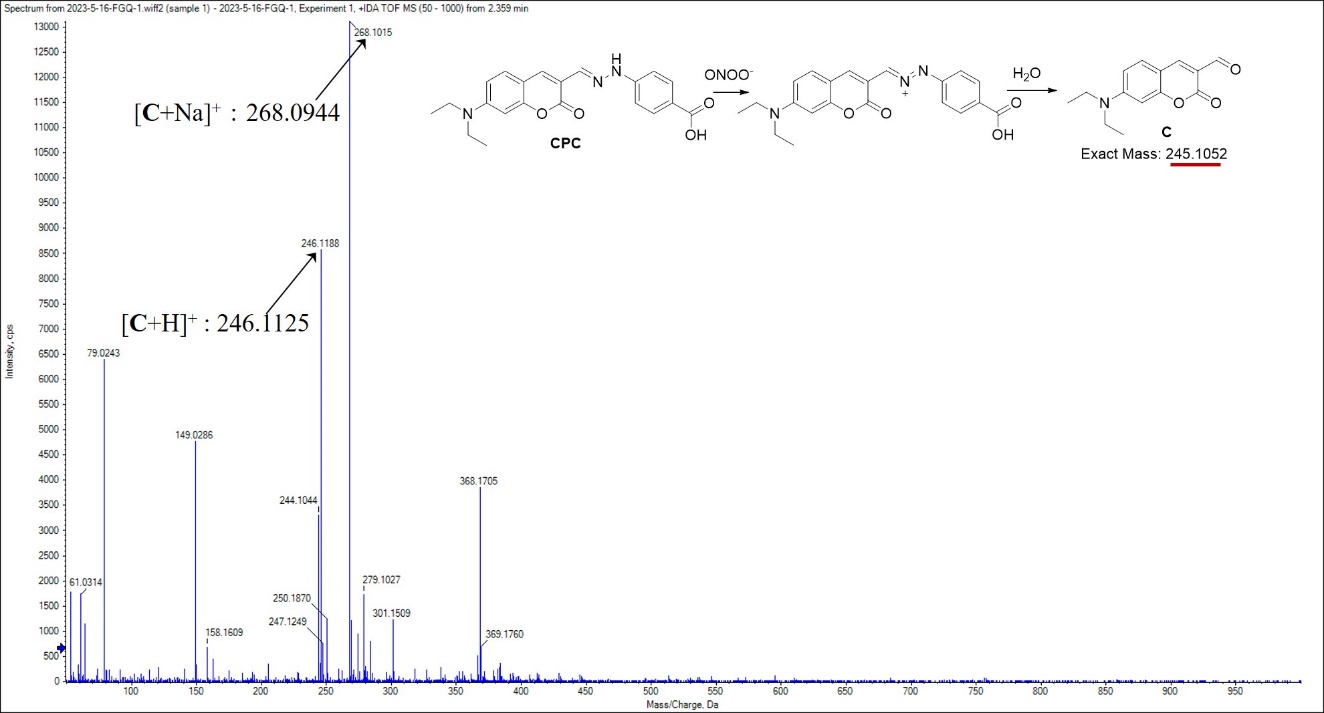


FIGURE S11 HRMS spectrum of CPC (0.1 mM) with ONOO^-^ (0.1 mM) in HEPES (0.01 M), at room temperature.

FIGURE S12 Fluorescence response of CPC (10.0 μM) to various related species (100.0 μM) in HEPES (0.01 M). Bars represent emission intensity at 582 nm at the excitation wavelength of 458 nm. 1: Blank; 2: Cd^2+^; 3: Co^2+^; 4: Cr^2+^; 5: Cs^+^; 6: Cu^2+^; 7: Li^+^; 8: Mg^2+^; 9: Mn^2+^; 10: CO_3_^2-^; 11: D-mannose; 12: D-ribose; 13: L-Ascorbic acid; 14: D-glucose 15: ClO^-^; 16: H_2_O_2_; 17: ^1^O_2_; 18: **⋅**OH; 19: NO; 20: t-BuOO**⋅**; 21: ONOO^-^. (λ_ex_ = 458 nm, λ_em_ = 582 nm, slits: 2.5/5 nm).


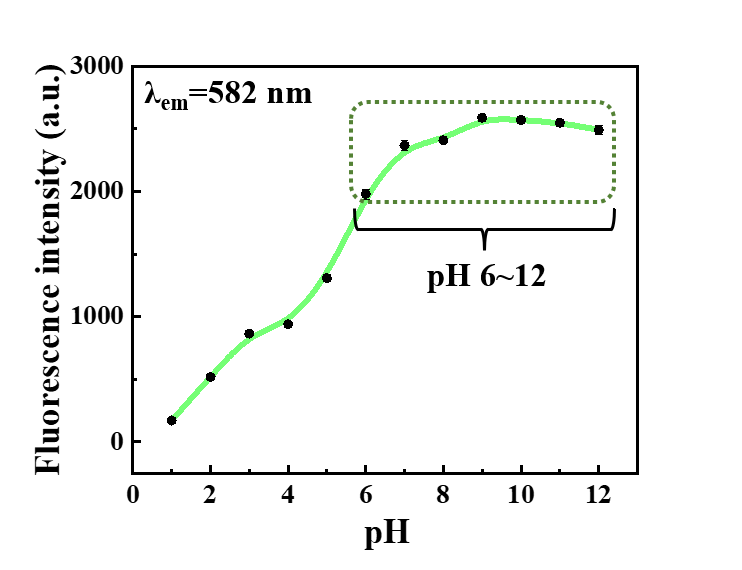


FIGURE S13 Fluorescence responses of CPC (10.0 μM) in HEPES (0.01 M) at different pH values (λ_ex_ = 458 nm, λ_em_ = 582 nm, slits: 2.5/5 nm).


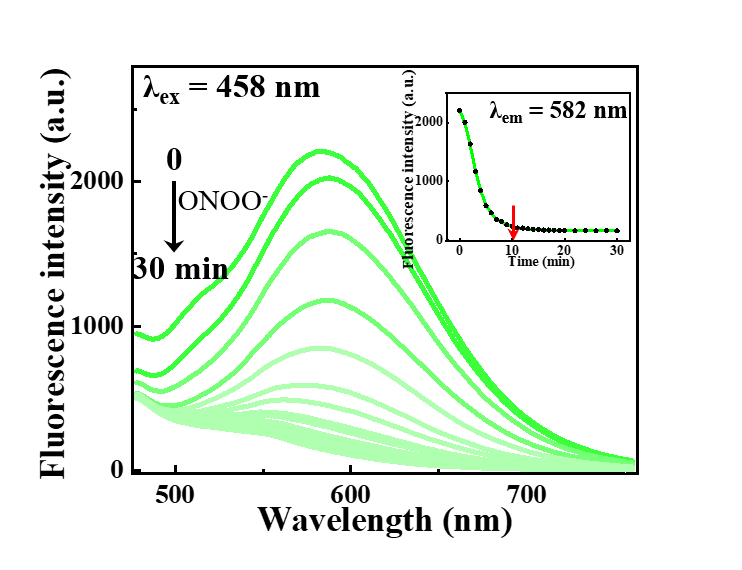


FIGURE S14 Fluorescence responses of CPC (10.0 μM) to ONOO^-^ (10.0 μM) in HEPES (0.01 M) over a period of 30 min (λ_ex_ = 458 nm, λ_em_ = 582 nm, slits: 2.5/5 nm). Inset: plot of the fluorescence intensity at 582 nm over a period of 30 min.


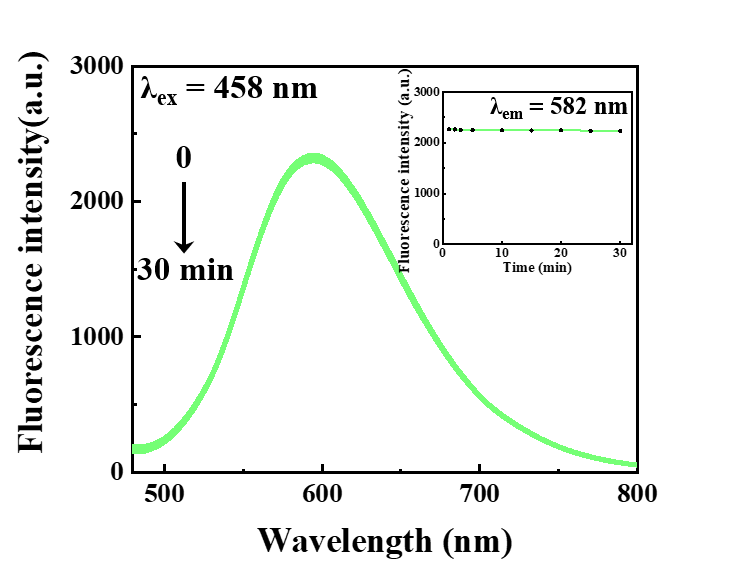


FIGURE S15 Fluorescence responses of CPC (10.0 μM) in HEPES (0.01 M) over a period of 30 min (λ_ex_ = 458 nm, λ_em_ = 582 nm, slits: 2.5/5 nm). Inset: plot of the fluorescence intensity at 582 nm over a period of 30 min.

FIGURE S16 Fluorescence lifetime of CPC (10.0 μM) in HEPES (0.01 M).

FIGURE S17 Quantitative analysis of CPC and Lipi‑Blue co-localization; data are mean ±SEM (n =10).


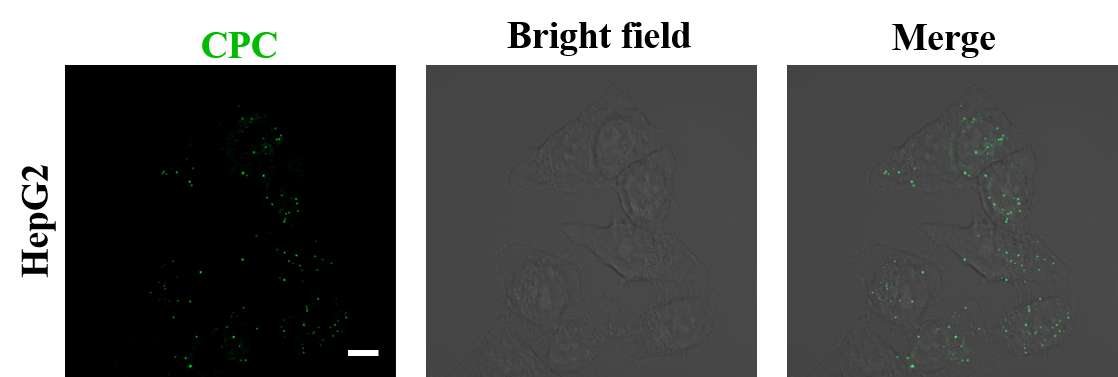


FIGURE S18 Microscopic images of HepG2 cells stained by CPC for 40 min (scale bar = 5 μm). **CPC**: 10.0 μM, λ_ex_ = 488 nm, λ_em_ = 500–550 nm.

FIGURE S19 Quantitative analysis of CPC and PKMTDR co-localization; data are mean ± SEM (n = 7).


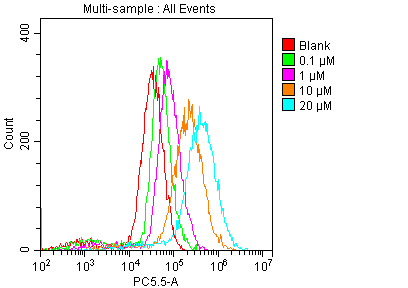


FIGURE S20 The permeability of the CPC at concentrations of 0.1–20.0 μM in HepG2 cells.


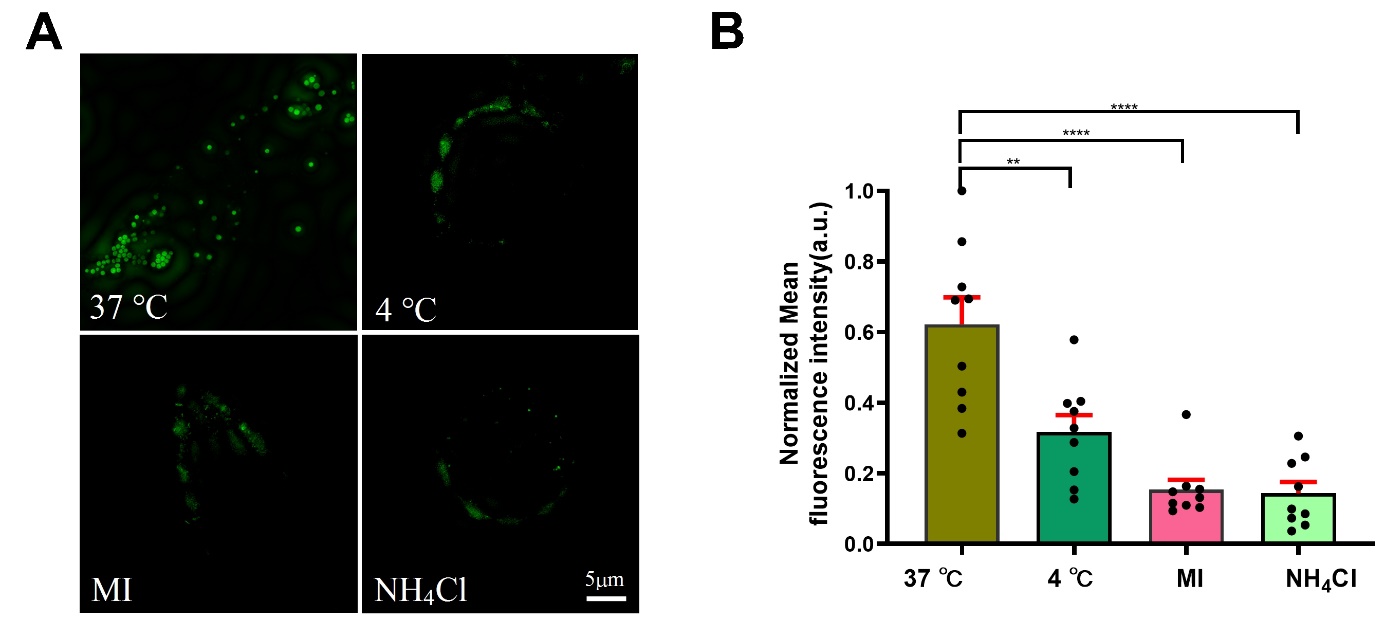


FIGURE S21 Investigation of the cell uptake of CPC by HepG2 cells via SIM imaging. (A) SIM images of lipid droplets labeled by **CPC**. At 37 °C, the cells were incubated with **CPC** for 40 min; at 4 °C, the cells were incubated with **CPC** for 40 min as well; with the metabolic inhibitors (MI), the cells were preincubated with 50.0 mM of 2-deoxy-D-glucose and 5.0 μM of oligomycin at 37 °C for 1 h and incubated with **CPC** at 37 °C for 40 min; last, the cells were preincubated with 50 mM of NH_4_Cl at 37 °C for 2 h and with **CPC** at 37 °C for 40 min. (B) Quantitative analysis of a. Data are mean ± SEM (*n* = 9 cells, ** *P* < 0.01, **** *P* < 0.0001). **CPC**: 10 μM, λ_ex_ = 405 nm, λ_em_ = 505–550 nm.

FIGURE S22 The cell viability of CPC at concentrations of 0.1–30.0 μM in HepG2 cells (n = 5 measurements).


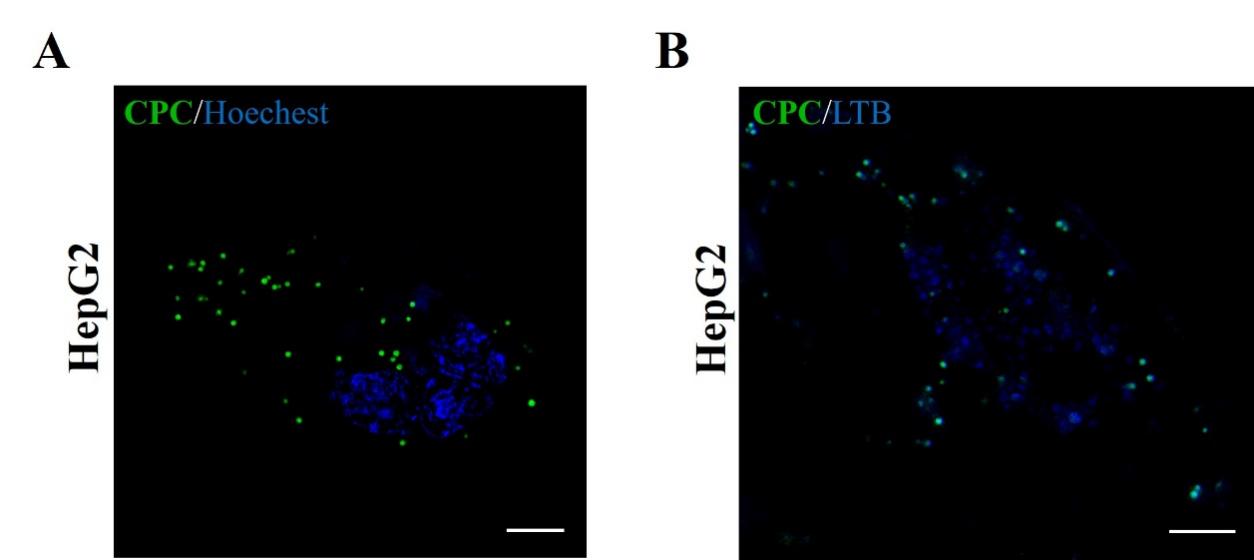


FIGURE S23 SIM images of cells stained with CPC and different organelles dye. (A) Merged SIM images of **CPC** and Hoechest (scale bar = 5 μm). (B) Merged SIM images of **CPC** and LTB (scale bar = 5 μm). **CPC**: 10 μM, λ_ex_ = 405 nm, λ_em_ = 505–550 nm; Hoechest: 100 μg/mL, λ_ex_ = 405 nm, λ_em_ = 417–476 nm; LTB: 200 nM, 405 nm, λ_em_ = 417–476 nm.


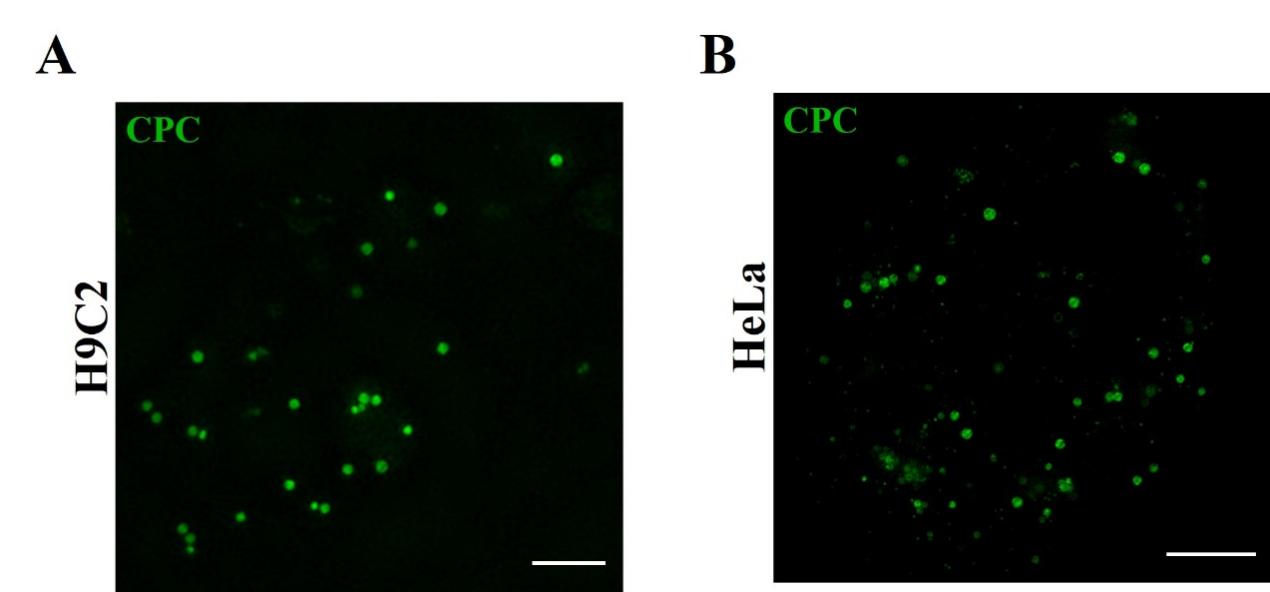


FIGURE S24 SIM images of different cells lines stained with CPC. (A) SIM image of H9C2 cells stained with **CPC**. (B) SIM image of HeLa cells stained with **CPC**. **CPC**: 10 μM, λ_ex_ = 405 nm, λ_em_ = 505–550 nm.


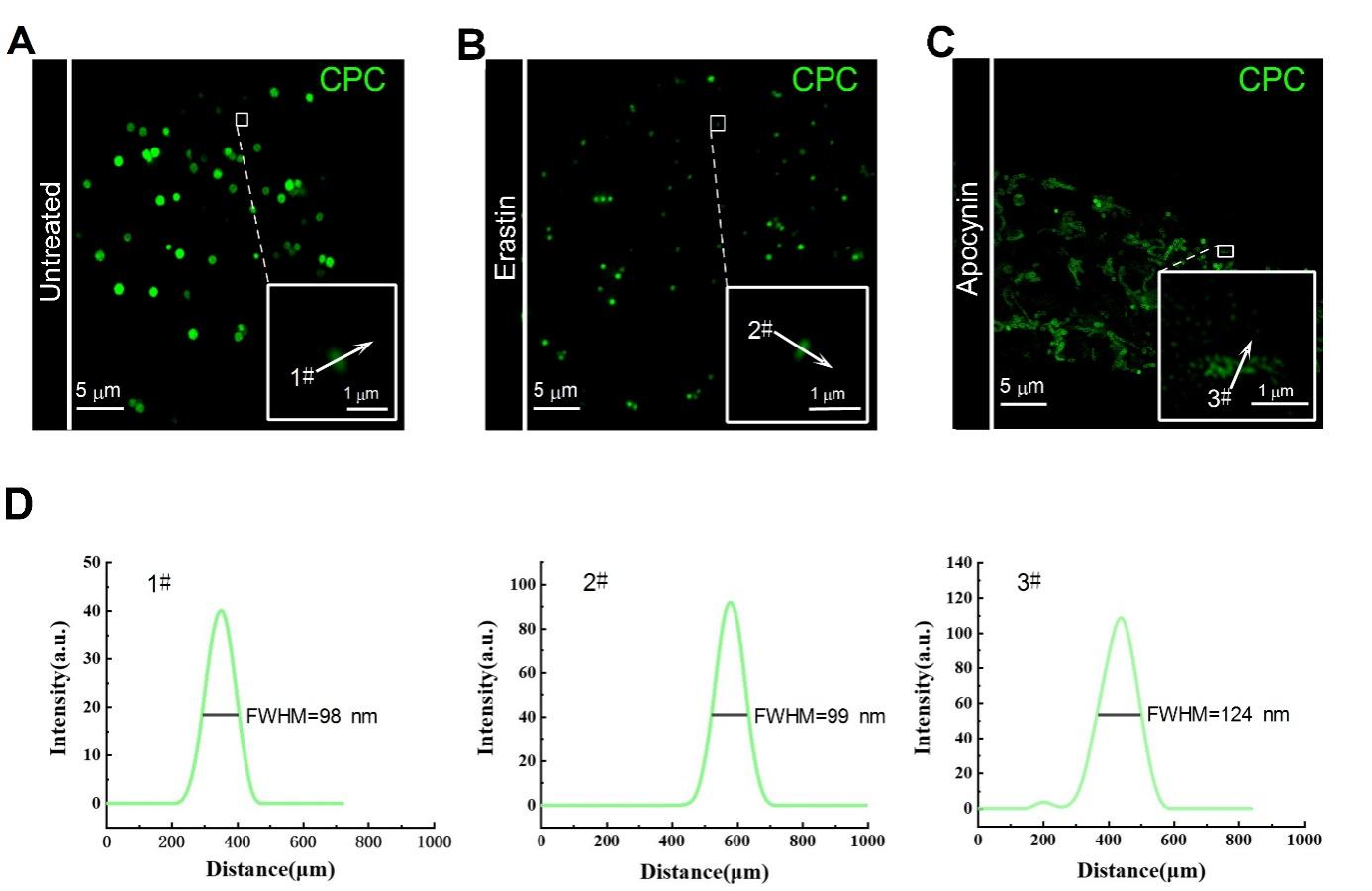


**FIGURE S25** SIM images of **CPC**-stained HepG2 cells after untreated (A) or erastin-treated (B) or apocynin-treated (C), and their FWHM (D), scale bar = 5 μm, enlarged images scale bar =1 μm. **CPC**: 10 μM, λ_ex_ = 405 nm, λ_em_ = 505–550 nm.


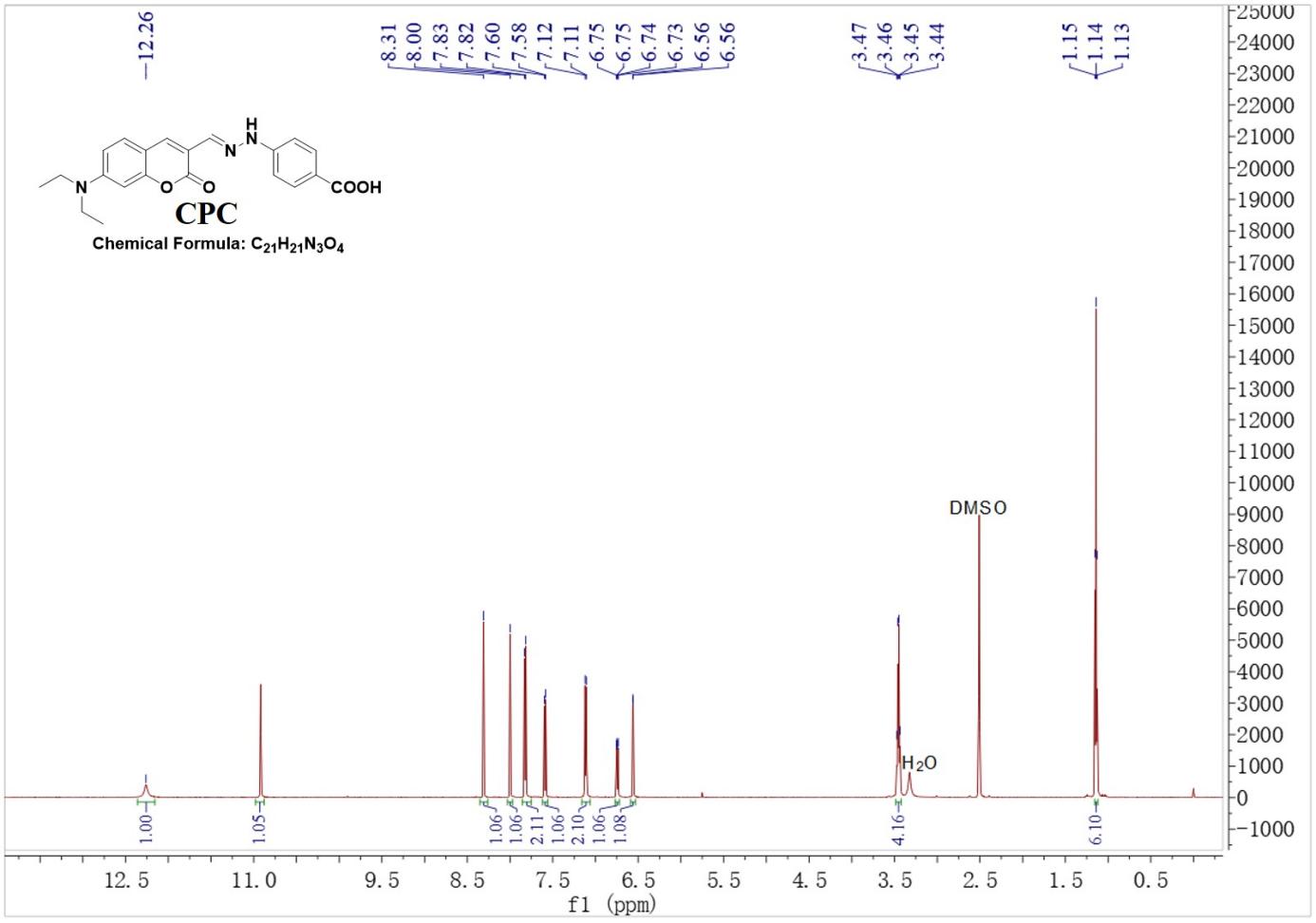


FIGURE S26 ^1^H NMR spectrum of CPC.


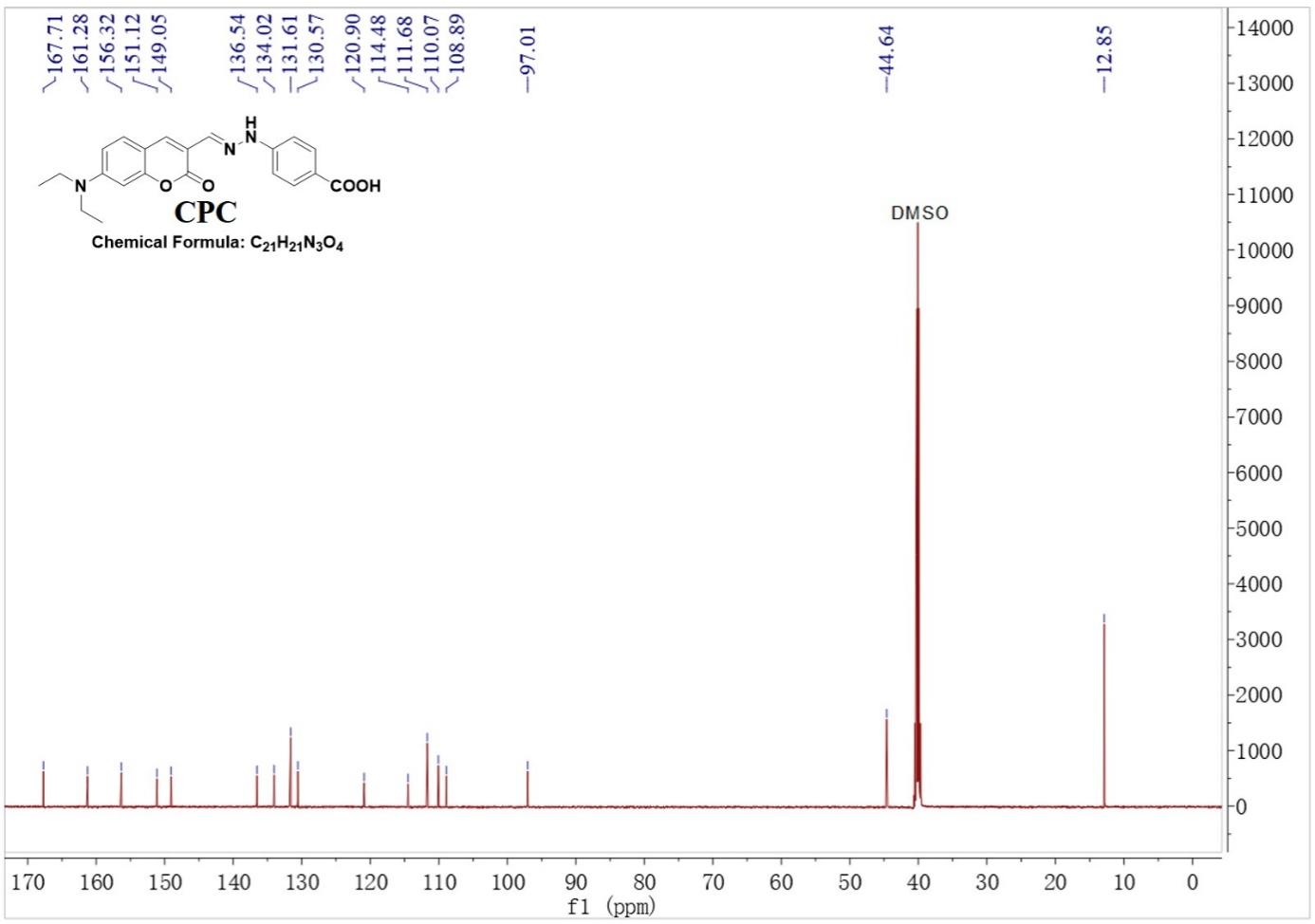


FIGURE S27 ^13^C NMR spectrum of CPC.


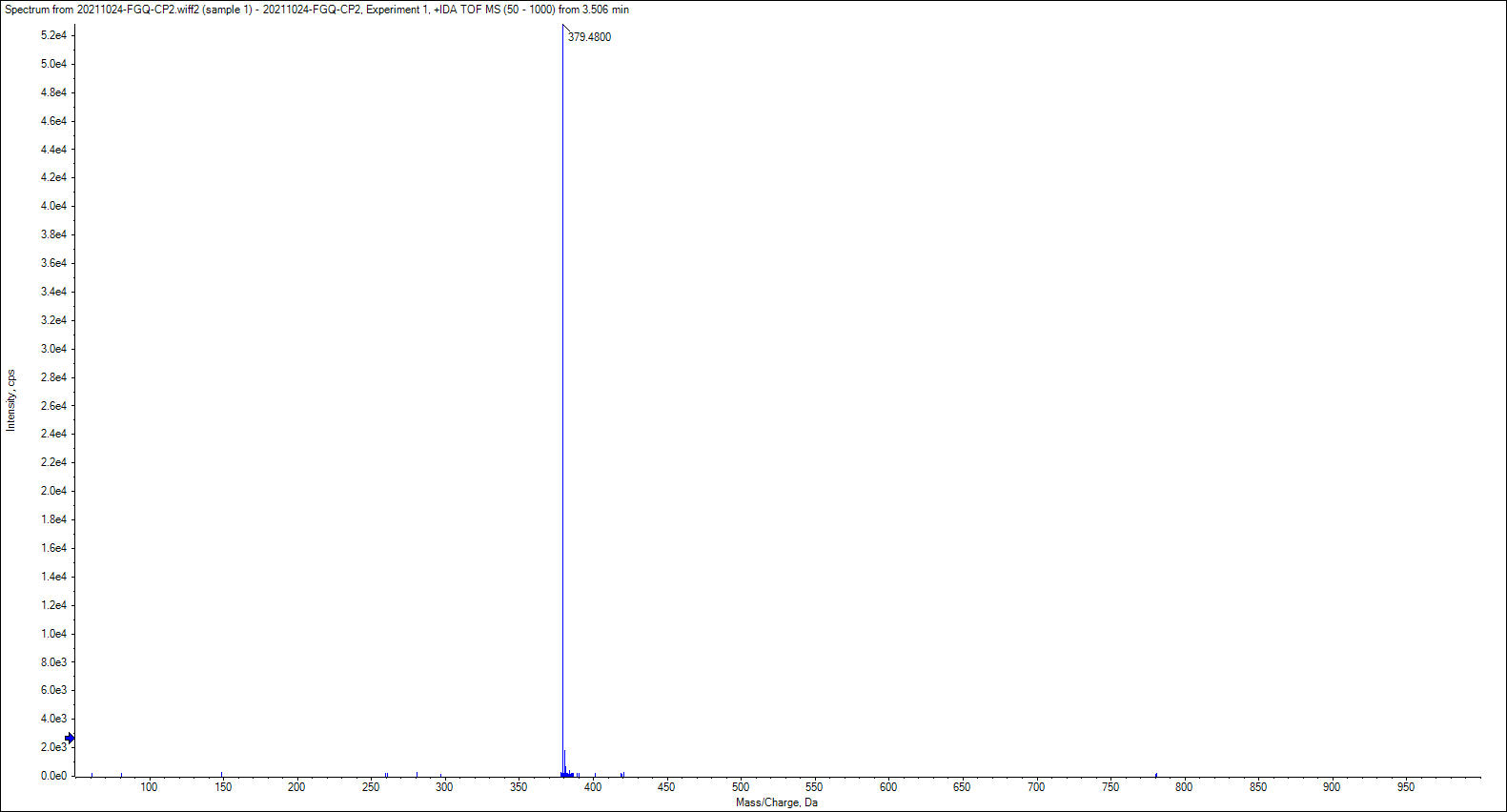


FIGURE S28 HRMS spectrum of CPC.

FIGURE S29 Synthesis of CPE: the synthesis operation is the same as that of CPC. ^1^H NMR (600 MHz, DMSO-*d_6_*) *δ* (ppm) (Figure S4): 8.33 (s, 1H), 8.19 (s, 1H), 7.53 (dd, *J* = 27.6, 19.9 Hz, 3H), 7.15 (s, 1H), 6.77 – 6.70 (m, 1H), 6.55 (dd, *J* = 7.7, 2.3 Hz, 1H), 6.04 (s, 1H), 4.02 (d, *J* = 6.6 Hz, 2H), 3.45 (q, *J* = 7.0 Hz, 4H), 1.28 (t, *J* = 7.1 Hz, 3H), 1.23 (s, 1H), 1.14 (t, *J* = 7.0 Hz, 6H). ^13^C NMR (151 MHz, DMSO-*d_6_*) *δ* (ppm) (Figure S5): 161.49, 156.33, 150.90, 142.53, 138.89, 138.88, 136.33, 136.29, 130.41, 114.01, 109.96, 109.02, 97.02, 46.14, 44.62, 14.21, 12.86. HRMS (Figure S6): For [M]^+^ m/z 365.1972. Found: [M]^+^ m/z 364.4373.


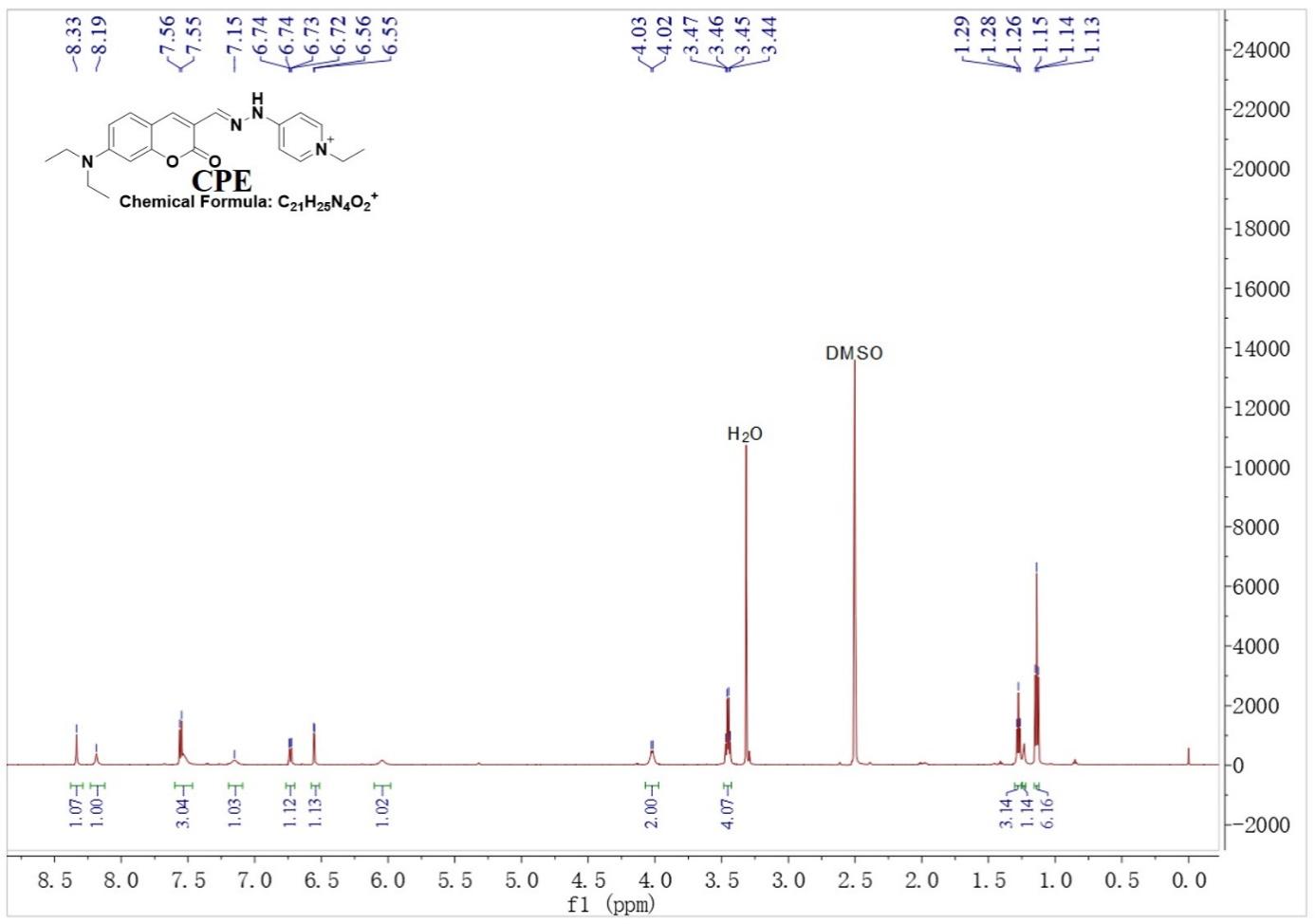


FIGURE S30 ^1^H NMR spectrum of CPE.


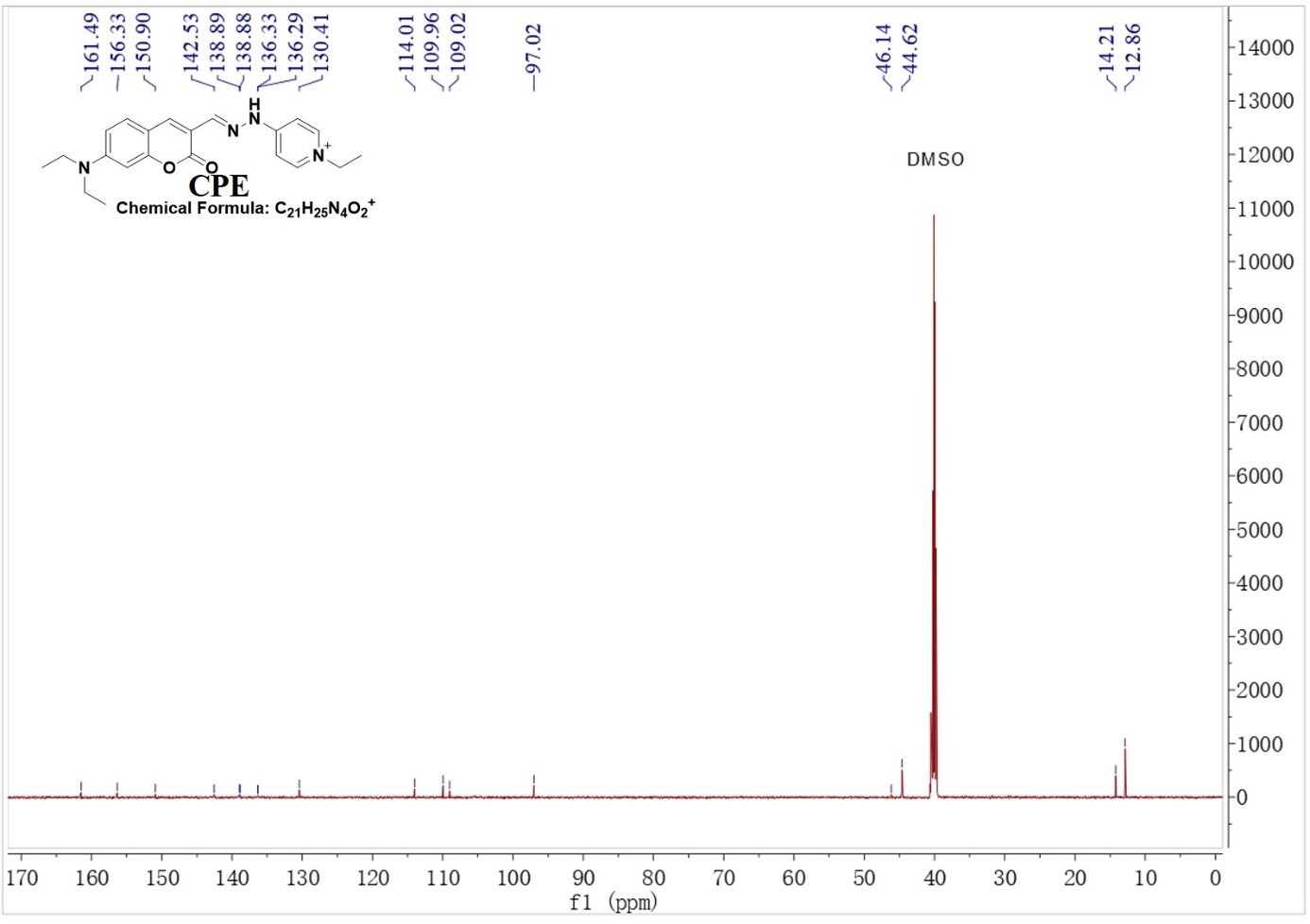


FIGURE S31 ^13^C NMR spectrum of CPE.


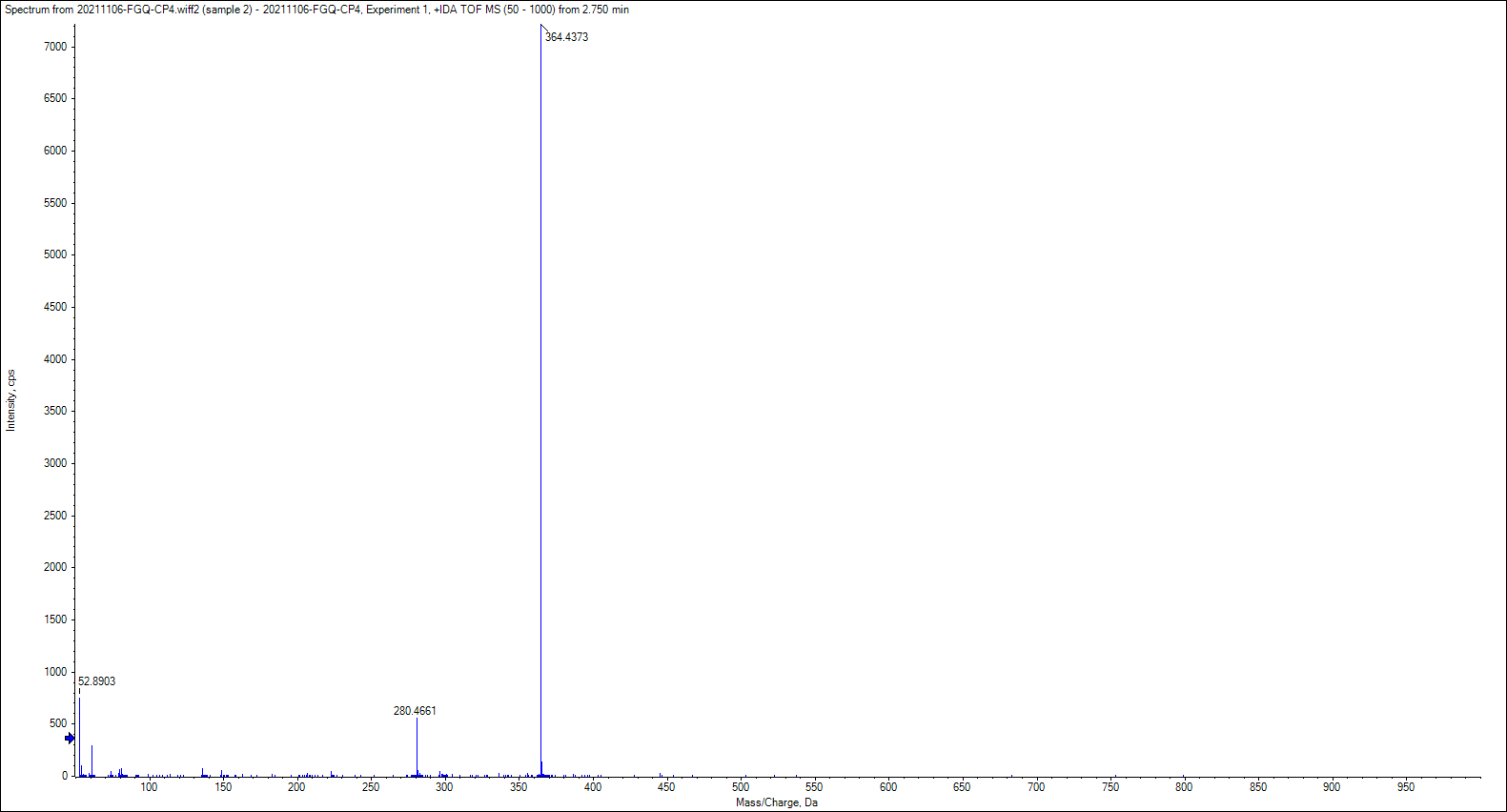


FIGURE S32 HRMS spectrum of CPE.

FIGURE S33 Synthesis of CPM: the synthesis operation is the same as that of CPC. ^1^H NMR (600 MHz, DMSO-*d_6_*) *δ* (ppm) (Figure S7): 11.00 (s, 1H), 8.31 (s, 1H), 8.11 (s, 1H), 8.06 (s, 1H), 7.64 (dd, *J* = 11.3, 4.2 Hz, 1H), 7.58 (d, *J* = 8.9 Hz, 1H), 7.28 (d, *J* = 8.4 Hz, 1H), 6.80 – 6.72 (m, 2H), 6.56 (d, *J* = 2.2 Hz, 1H), 3.46 (q, *J* = 7.0 Hz, 4H), 1.14 (t, *J* = 7.1 Hz, 6H). ^13^C NMR (151 MHz, DMSO-*d_6_*) *δ* (ppm) (Figure S8): 161.27, 156.32, 151.08, 138.35, 136.56, 133.88, 130.54, 115.42, 114.69, 110.04, 108.85, 106.98, 96.99, 44.63, 12.86. HRMS (Figure S9): For [M+H]^+^ m/z 336.3950. Found: [M+H]^+^ m/z 337.1665.


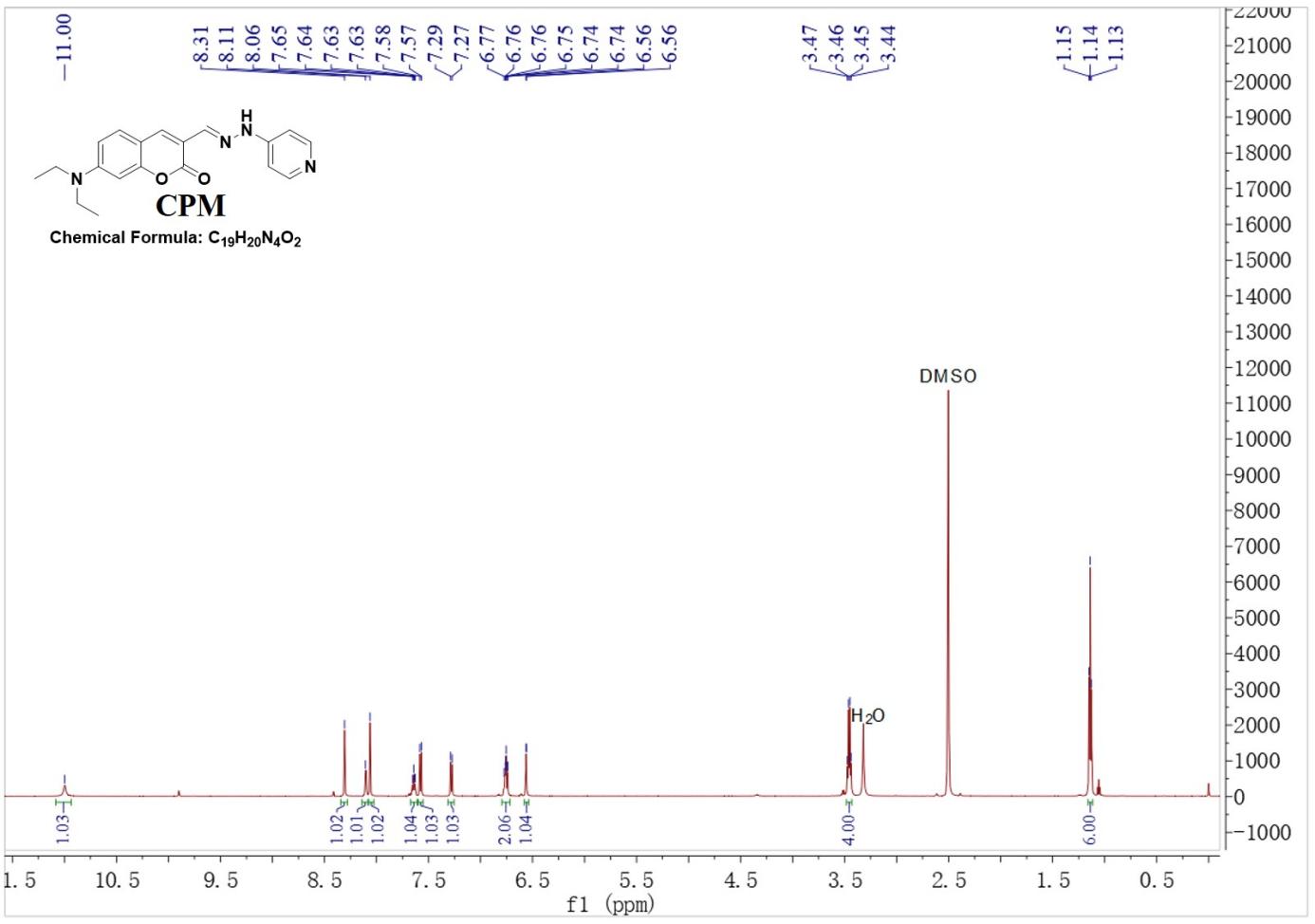


FIGURE S34 ^1^H NMR spectrum of CPM.


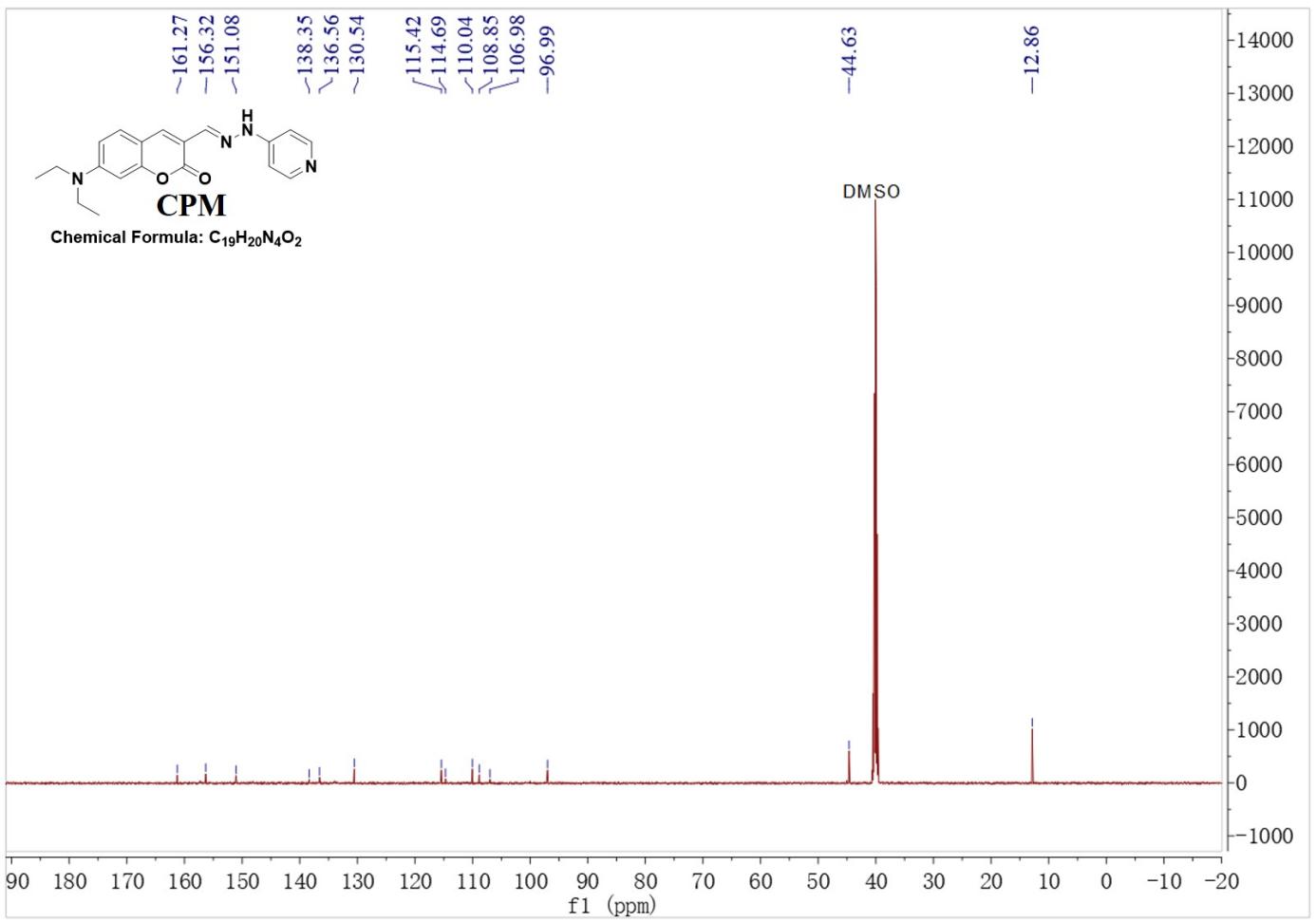


FIGURE S35 ^13^C NMR spectrum of CPM.

FIGURE S36 HRMS spectrum of CPM.

FIGURE S37 Synthesis of CPB: the synthesis operation is the same as that of CPC. ^1^H NMR (600 MHz, DMSO-*d_6_*) *δ* (ppm) (Figure S10): 10.47 (s, 1H), 8.24 (s, 1H), 7.91 (s, 1H), 7.57 (d, *J* = 8.9 Hz, 1H), 7.22 (dd, *J* = 8.3, 7.4 Hz, 2H), 7.08 (d, *J* = 7.6 Hz, 2H), 6.78 – 6.71 (m, 2H), 6.55 (d, *J* = 2.2 Hz, 1H), 3.44 (q, *J* = 7.0 Hz, 4H), 1.14 (t, *J* = 7.0 Hz, 6H). ^13^C NMR (151 MHz, DMSO-*d_6_*) *δ* (ppm) (Figure S11): 161.36, 156.04, 150.81, 145.59, 135.42, 131.30, 130.29, 129.53, 119.32, 115.22, 112.53, 109.97, 109.00, 97.04, 44.60, 12.85. HRMS (Figure S12): For [M+H]^+^ m/z 336.1712. Found: [M+H]^+^ m/z 335.6197.


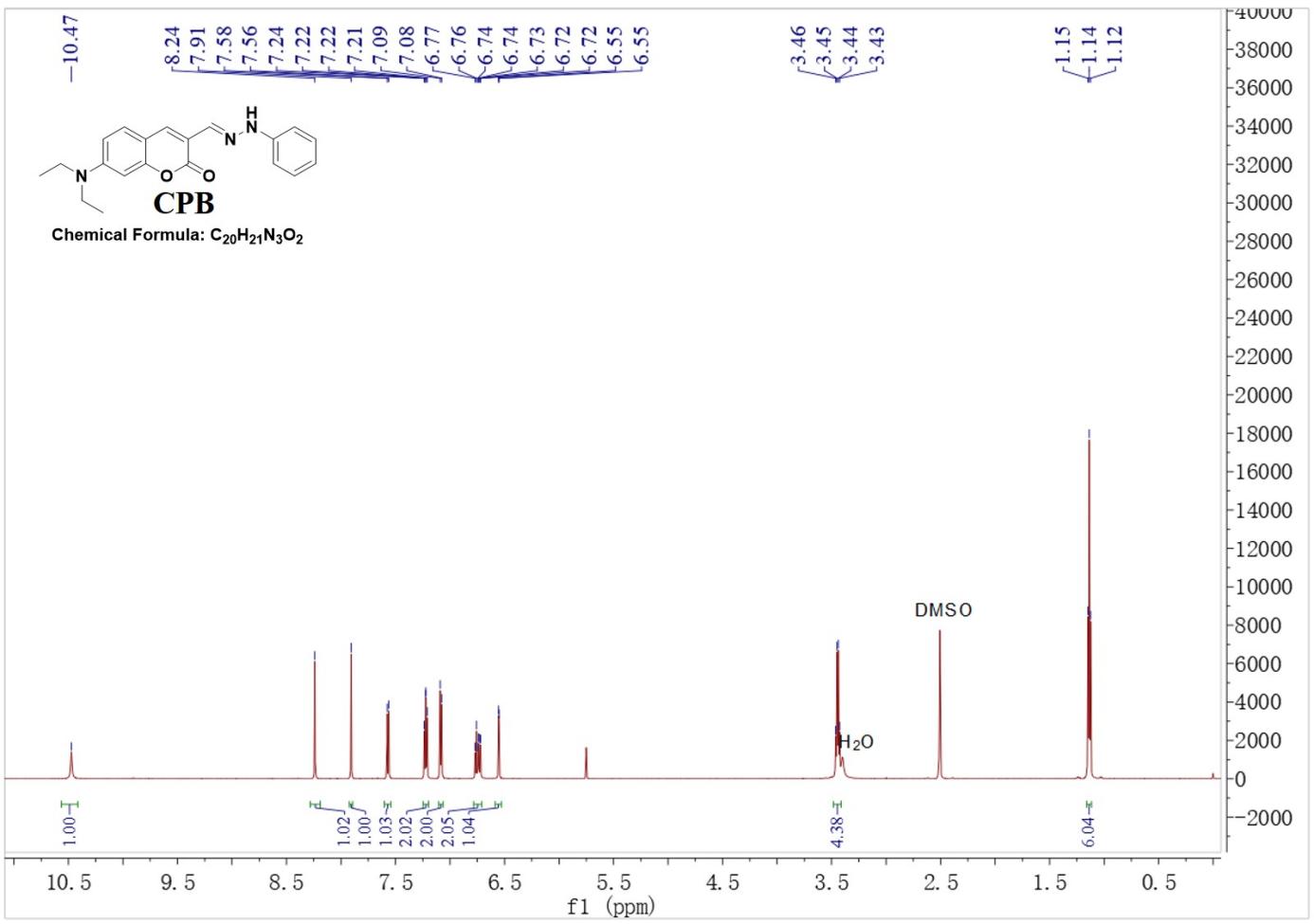


FIGURE S38 ^1^H NMR spectrum of CPB.


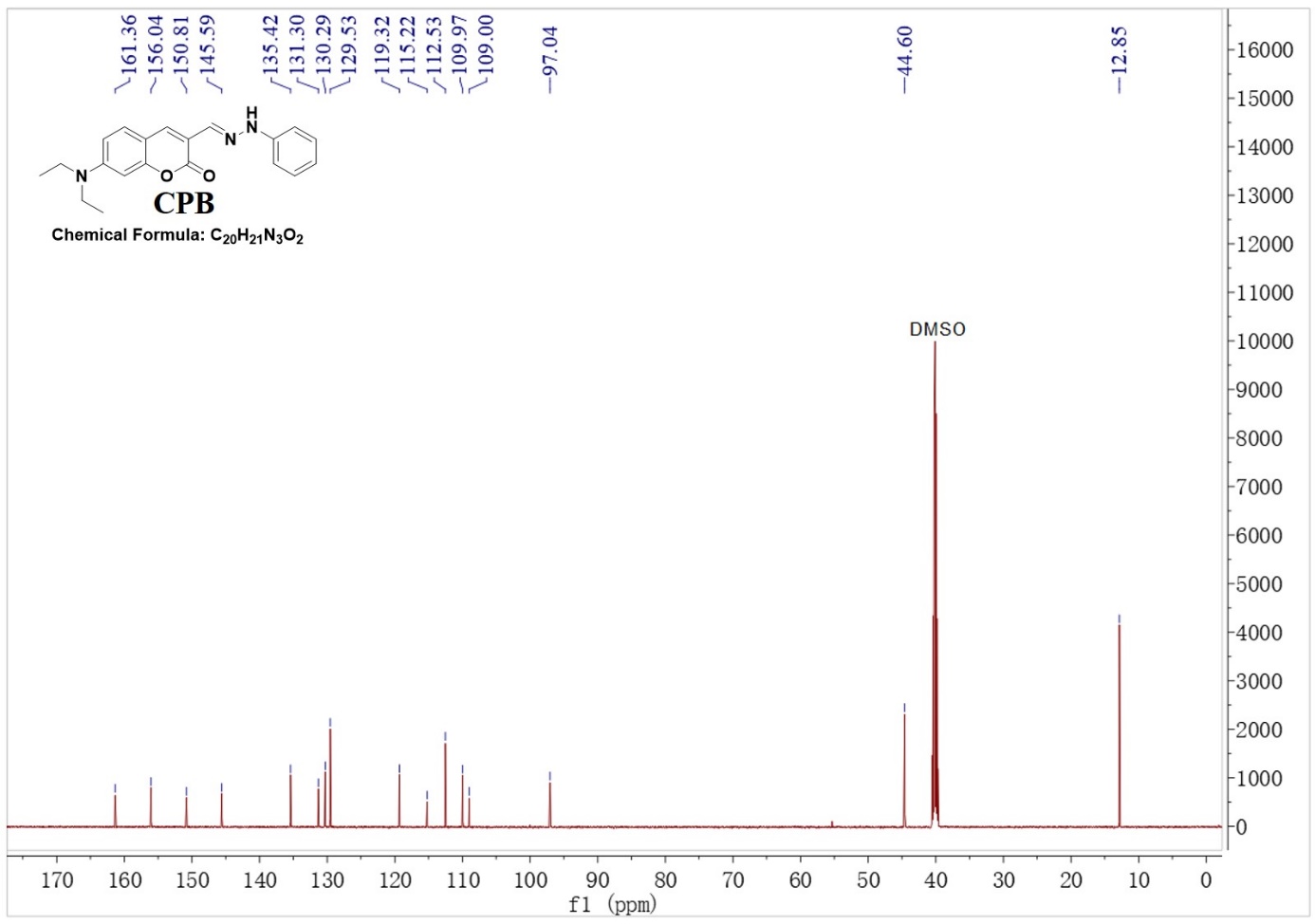


FIGURE S39 ^13^C NMR spectrum of CPB.


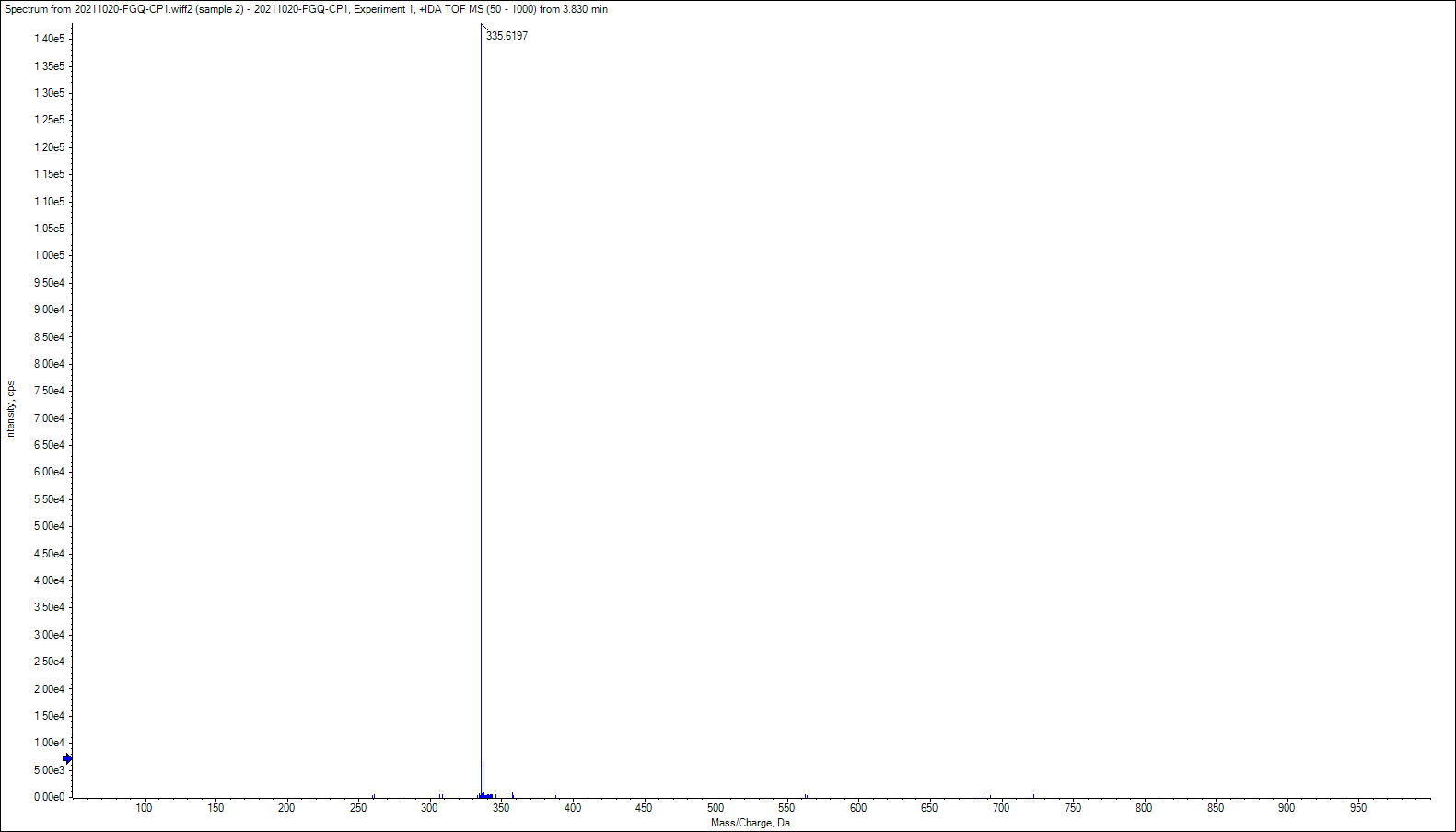


FIGURE S40 HRMS spectrum of CPB.

FIGURE S41 Synthesis of CHE: the synthesis operation is the same as that of CPC. ^1^H NMR (600 MHz, DMSO-*d_6_*) *δ* (ppm) (Figure S13): 8.87 (s, 1H), 8.30 (d, *J* = 15.7 Hz, 1H), 7.93 – 7.81 (m, 3H), 7.65 – 7.54 (m, 3H), 6.93 (dd, *J* = 9.1, 2.3 Hz, 1H), 6.72 (d, *J* = 2.1 Hz, 1H), 4.50 (d, *J* = 7.3 Hz, 2H), 3.58 (q, *J* = 7.0 Hz, 4H), 1.78 (s, 6H), 1.47 (t, *J* = 7.3 Hz, 3H), 1.19 (t, *J* = 7.1 Hz, 6H). ^13^C NMR (151 MHz, DMSO-*d_6_*) *δ* (ppm) (Figure S14): 180.84, 159.88, 158.18, 154.59, 150.70, 150.47, 143.83, 141.06, 132.89, 129.53, 129.09, 123.47, 114.82, 112.74, 111.88, 109.99, 109.86, 97.12, 51.96, 45.36, 42.12, 26.62, 13.48, 12.98. HRMS (Figure S15): For [M]^+^ m/z 415.2380. Found: [M]^+^ m/z 414.0684.


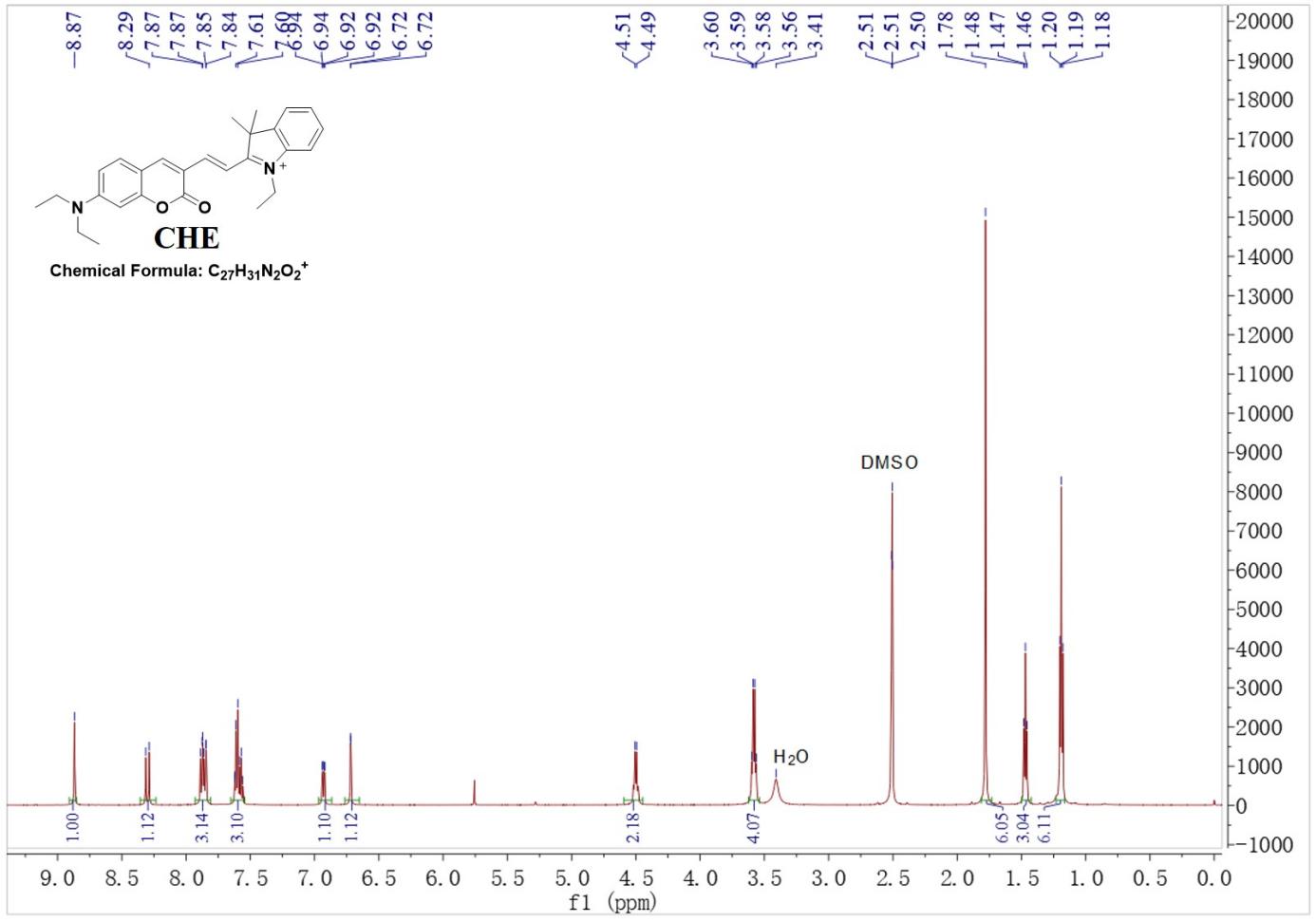


FIGURE S42 ^1^H NMR spectrum of CHE.


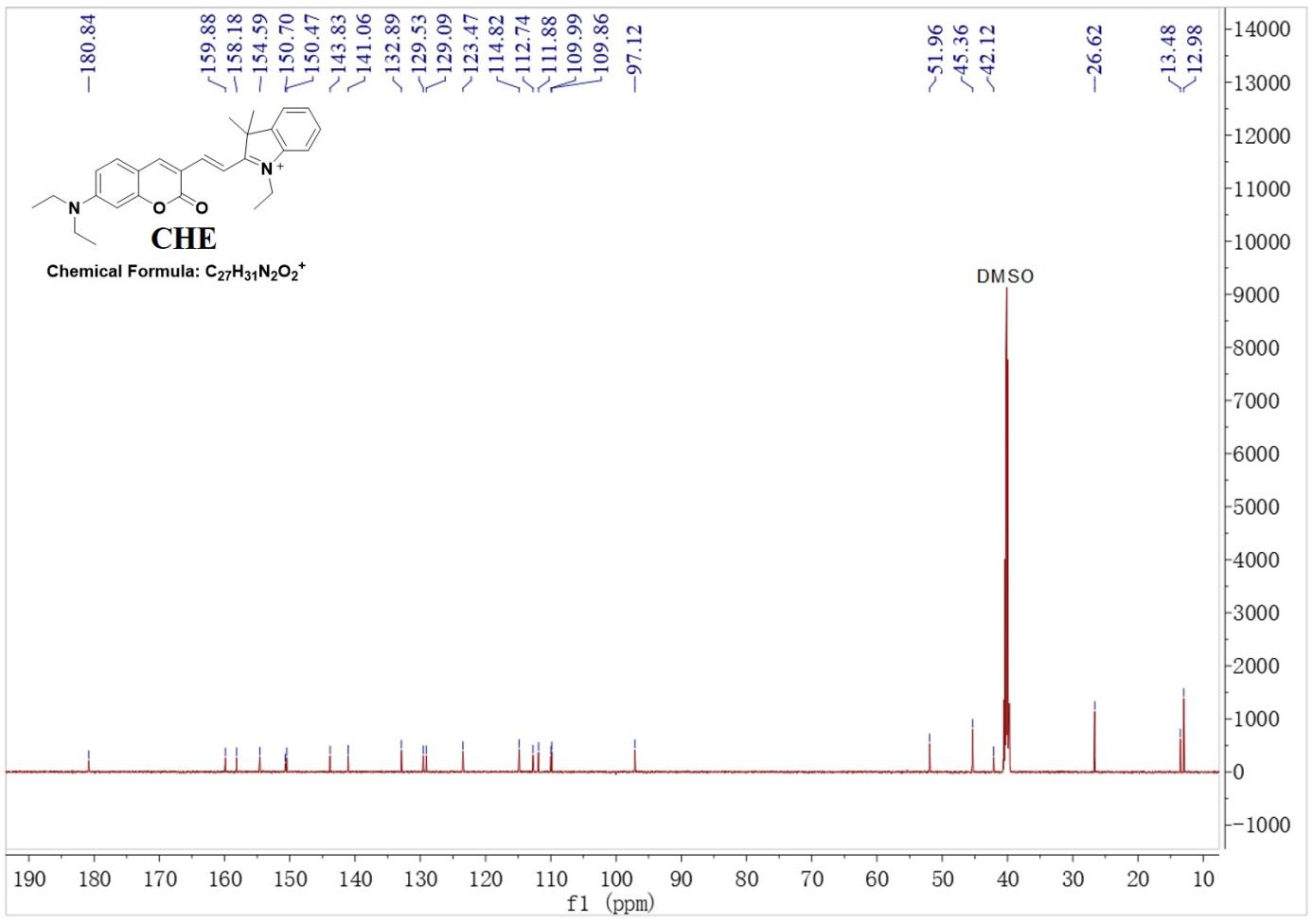


FIGURE S43 ^13^C NMR spectrum of CHE.


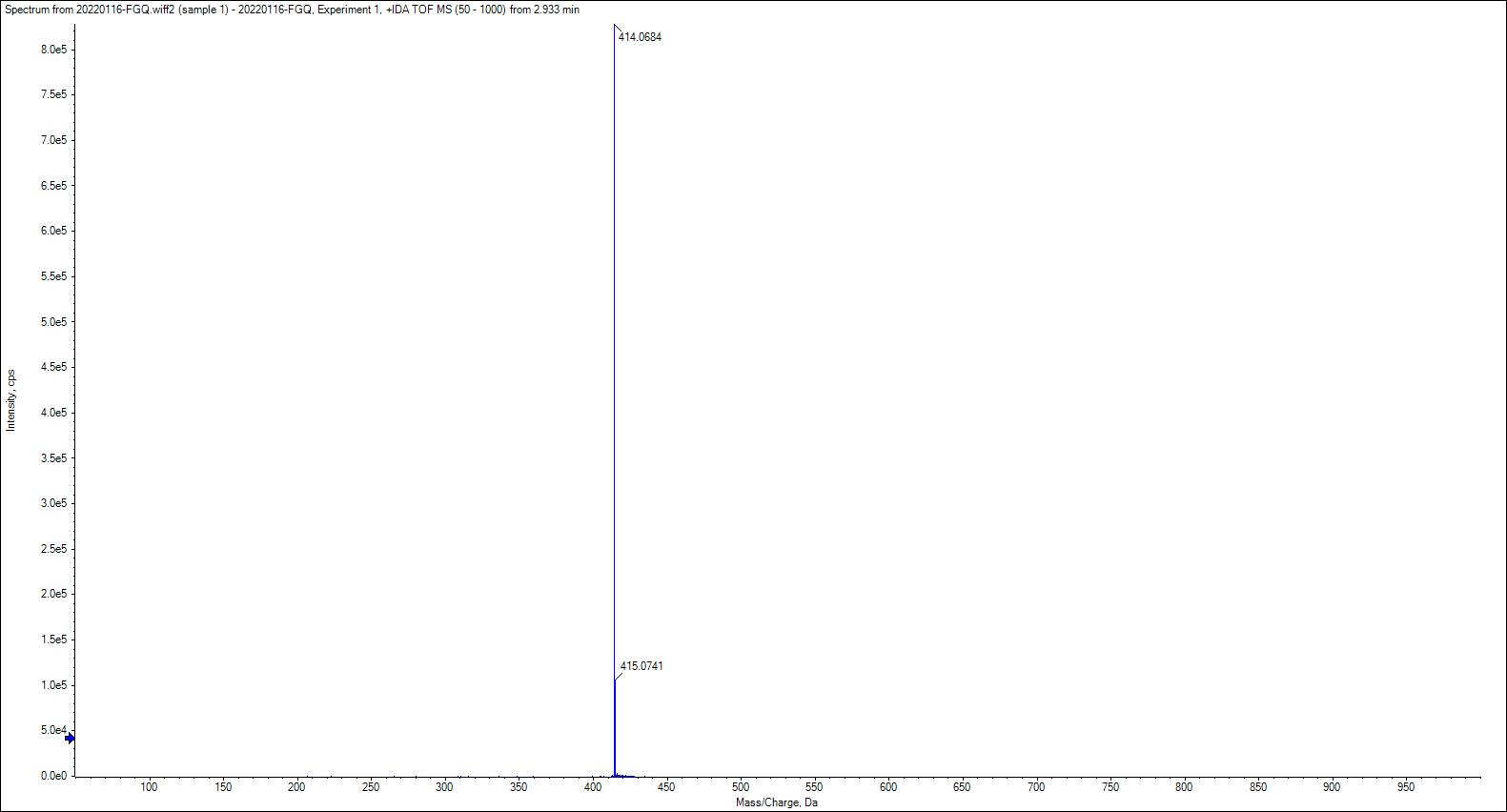


FIGURE S44 HRMS spectrum of CHE.

FIGURE S45. Synthesis of CPS2: the synthesis operation is the same as that of CPC. ^1^H NMR (600 MHz, DMSO-*d_6_*) *δ* (ppm) (Figure S16): 10.37 (s, 1H), 8.89 (s, 1H), 8.38 (d, *J* = 15.4 Hz, 2H), 8.19 (dd, *J* = 8.4, 1.5 Hz, 1H), 8.00 (d, *J* = 8.5 Hz, 1H), 7.87 (d, *J* = 15.7 Hz, 1H), 7.82 (d, *J* = 8.4 Hz, 2H), 7.77 (d, *J* = 8.5 Hz, 2H), 7.61 (d, *J* = 9.2 Hz, 1H), 6.95 (dd, *J* = 9.2, 2.2 Hz, 1H), 6.74 (d, *J* = 2.0 Hz, 1H), 4.53 (d, *J* = 7.3 Hz, 2H), 3.60 (d, *J* = 7.1 Hz, 4H), 3.45 (d, *J* = 7.0 Hz, 2H), 1.85 (s, 6H), 1.49 (t, *J* = 7.3 Hz, 3H), 1.20 (t, *J* = 7.1 Hz, 6H). ^13^C NMR (151 MHz, DMSO-*d_6_*) *δ* (ppm) (Figure S17): 182.09, 164.98, 159.80, 158.38, 154.95, 151.78, 143.69, 143.44, 141.03, 135.15, 133.18, 129.46, 129.11, 122.94, 119.74, 114.49, 112.79, 112.13, 110.32, 109.57, 97.22, 65.48, 56.51, 51.97, 45.48, 42.27, 26.73, 19.00, 13.39, 13.00. HRMS (Figure S18): For [M]^+^ m/z 578.2821. Found: [M]^+^ m/z 578.2603.


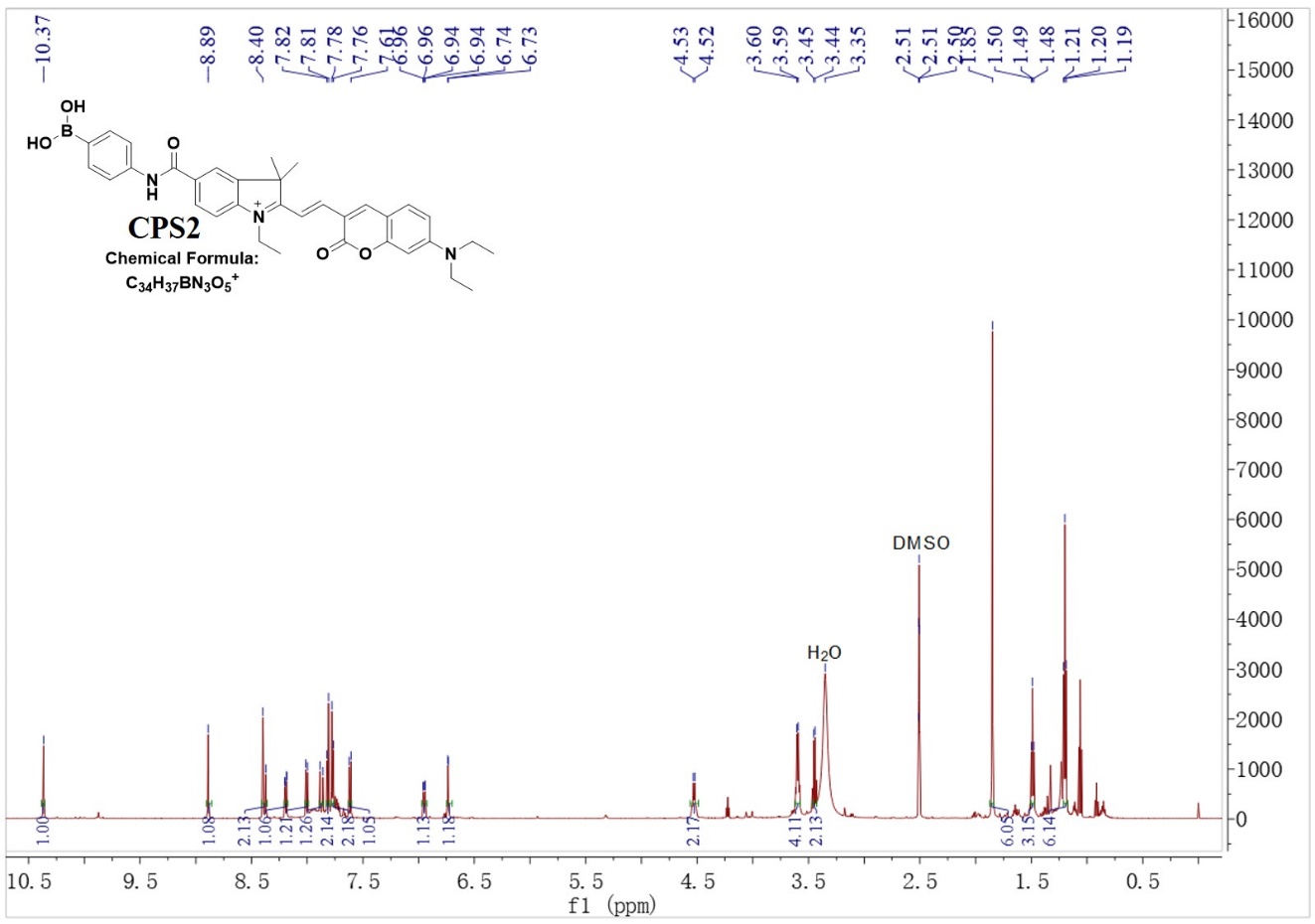


FIGURE S46 ^1^H NMR spectrum of CPS2.


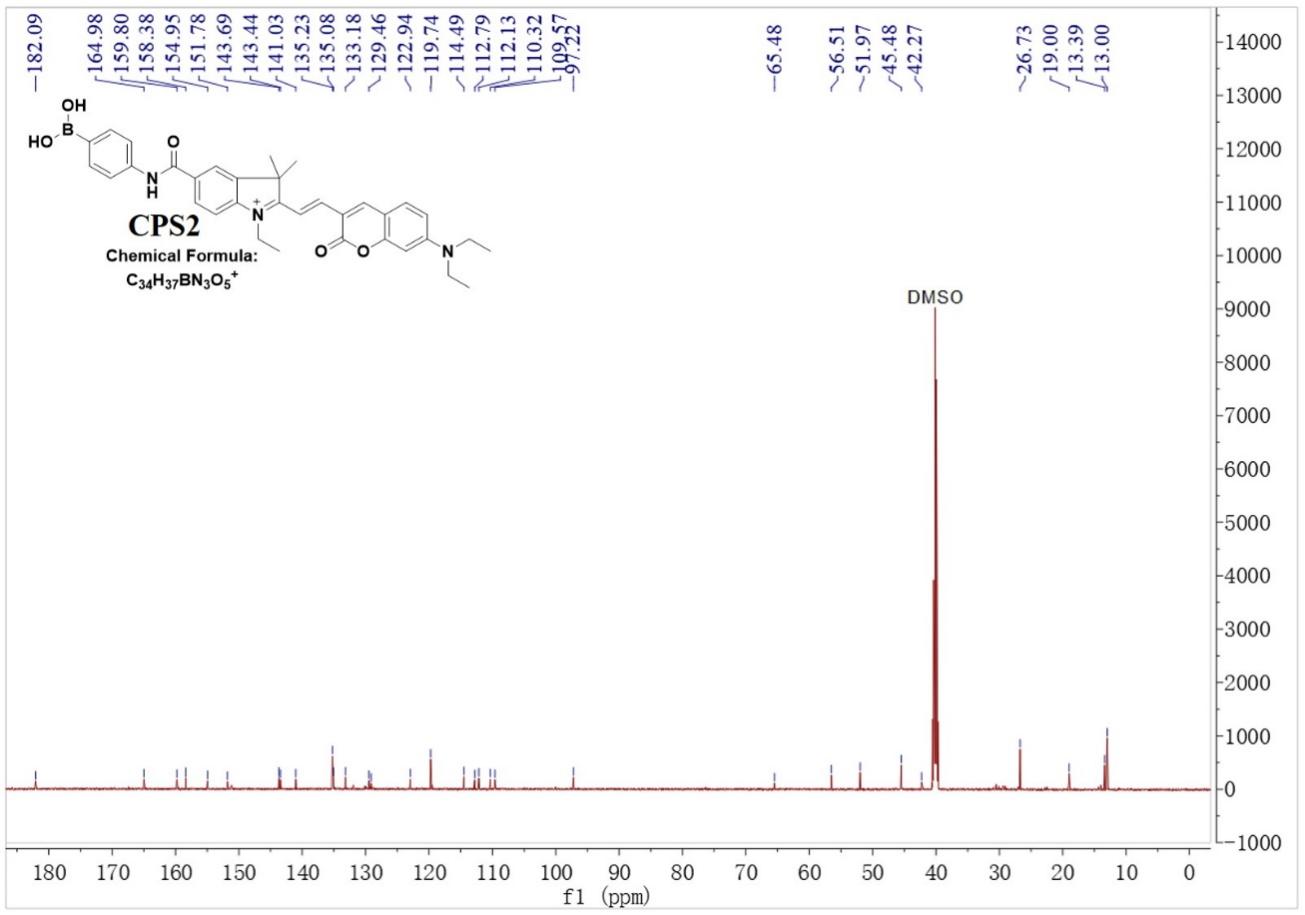


FIGURE S47 ^13^C NMR spectrum of CPS2.

FIGURE S48 HRMS spectrum of CPS2.

FIGURE S49 Synthesis of CPC2: the synthesis operation is the same as that of CPC. ^1^H NMR (600 MHz, DMSO-*d_6_*) *δ* (ppm) (Figure S19): 8.90 (s, 1H), 8.41 (s, 1H), 8.38 (d, *J* = 4.5 Hz, 1H), 8.15 (d, *J* = 8.4 Hz, 1H), 7.96 (d, *J* = 8.5 Hz, 1H), 7.86 (d, *J* = 15.6 Hz, 1H), 7.61 (d, *J* = 9.1 Hz, 1H), 6.95 (d, *J* = 9.3 Hz, 1H), 6.74 (s, 1H), 4.49 (d, *J* = 7.2 Hz, 2H), 3.60 (dd, *J* = 13.7, 6.7 Hz, 4H), 1.82 (s, 6H), 1.46 (t, *J* = 7.2 Hz, 3H), 1.19 (t, *J* = 7.0 Hz, 6H). ^13^C NMR (151 MHz, DMSO-*d_6_*) *δ* (ppm) (Figure S20): 182.37, 167.05, 159.78, 158.38, 154.96, 152.18, 151.44, 144.50, 143.93, 133.27, 131.06,130.97, 124.37, 114.66, 112.72, 112.15, 110.33, 109.41, 97.18, 51.88, 45.48, 42.22, 26.56, 13.33, 13.00. HRMS (Figure S21): For [M]^+^ m/z 459.2278. Found: [M]^+^ m/z 459.2086.


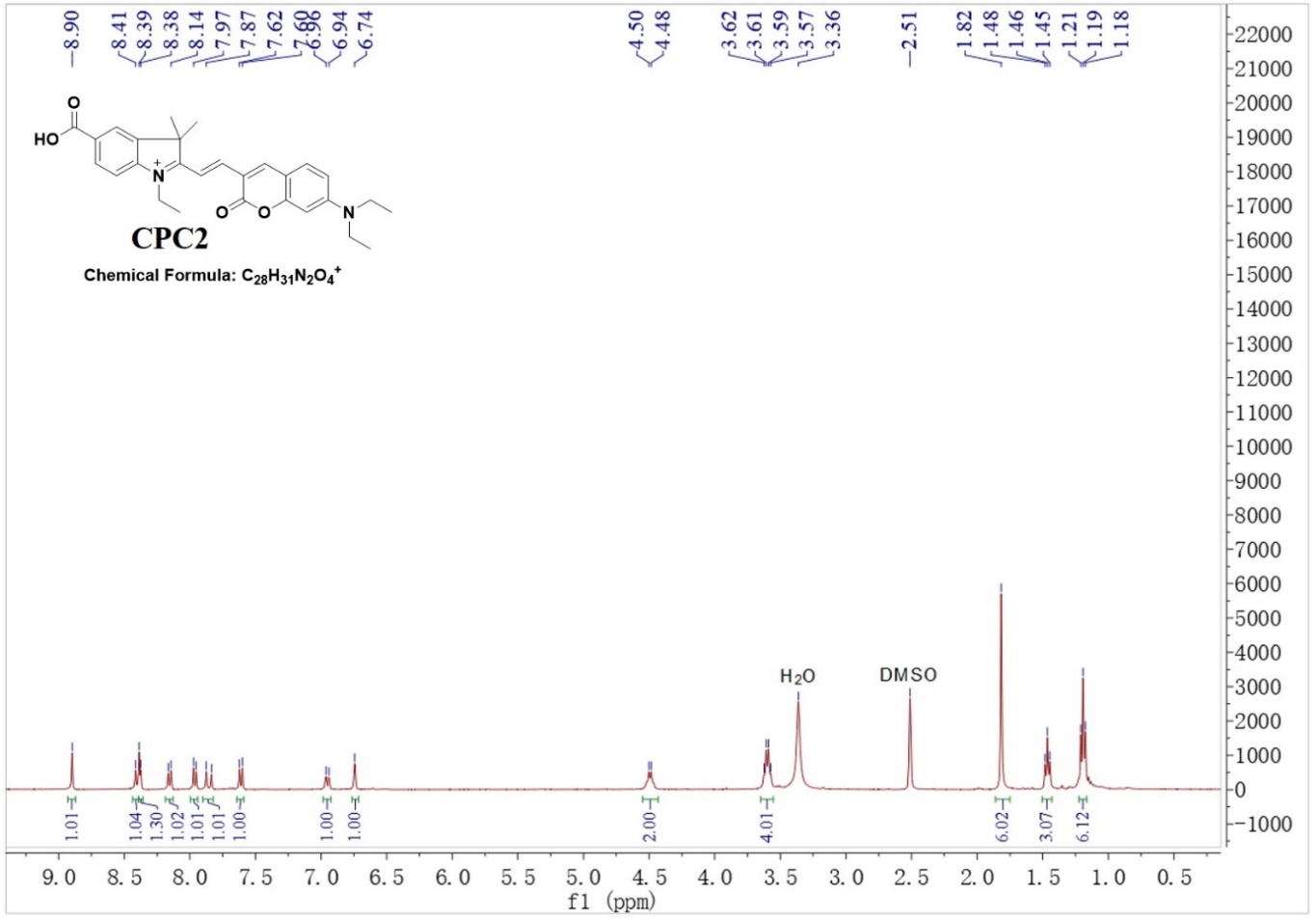


FIGURE S50 ^1^H NMR spectrum of CPC2.


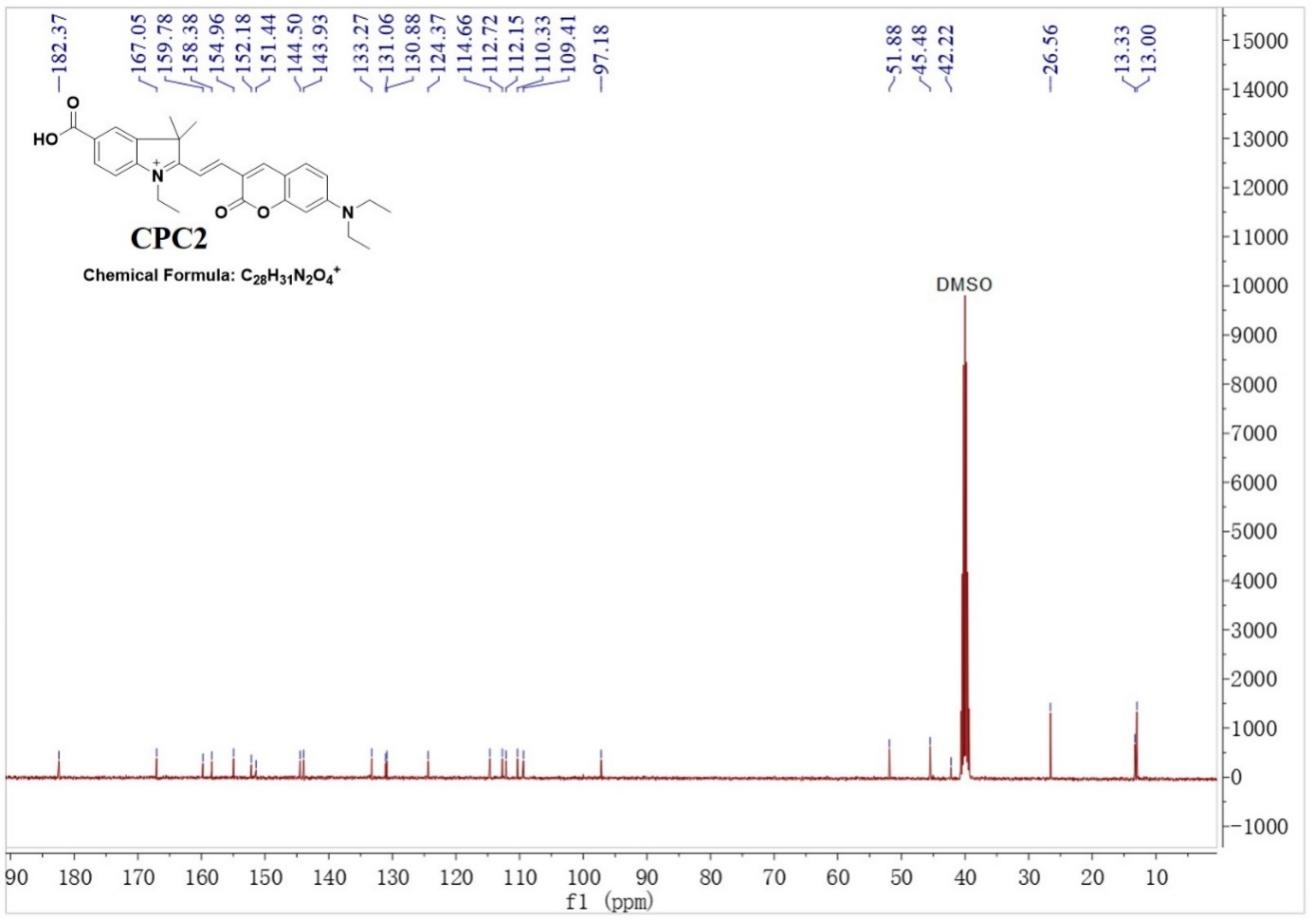


FIGURE S51 ^13^C NMR spectrum of CPC2.

FIGURE S52 HRMS spectrum of CPC2.

FIGURE S53 Synthesis of CHS: the synthesis operation is the same as that of CPC. ^1^H NMR (600 MHz, DMSO-*d_6_*) *δ* (ppm) (Figure S22): 9.06 (s, 1H), 8.39 (s, 1H), 8.36 (s, 1H), 8.13 (d, *J* = 8.1 Hz, 1H), 8.00 (d, *J* = 8.4 Hz, 1H), 7.85 (d, *J* = 15.7 Hz, 1H), 7.64 (d, *J* = 9.2 Hz, 1H), 6.95 (d, *J* = 9.0 Hz, 1H), 6.73 (s, 1H), 4.50 (s, 2H), 3.59 (d, *J* = 6.7 Hz, 4H), 2.57 (d, *J* = 6.5 Hz, 2H), 2.00 (s, 2H), 1.79 (s, 6H), 1.23 (s, 2H), 1.19 (t, *J* = 6.6 Hz, 6H). ^13^C NMR (151 MHz, DMSO-*d_6_*) *δ* (ppm) (Figure S23): 182.35, 167.05, 160.10, 158.51, 154.94, 151.30, 144.89, 143.72, 133.43, 132.00, 131.04, 130.12, 129.13, 124.26, 114.92, 112.83, 112.10, 110.48, 109.52, 97.25, 65.49, 51.76, 50.50, 45.48, 30.47, 26.82, 22.81, 19.12, 14.02, 13.00. HRMS (Figure S24): For [M]^+^ m/z 567.2165. Found: [M]^+^ m/z 567.1946.


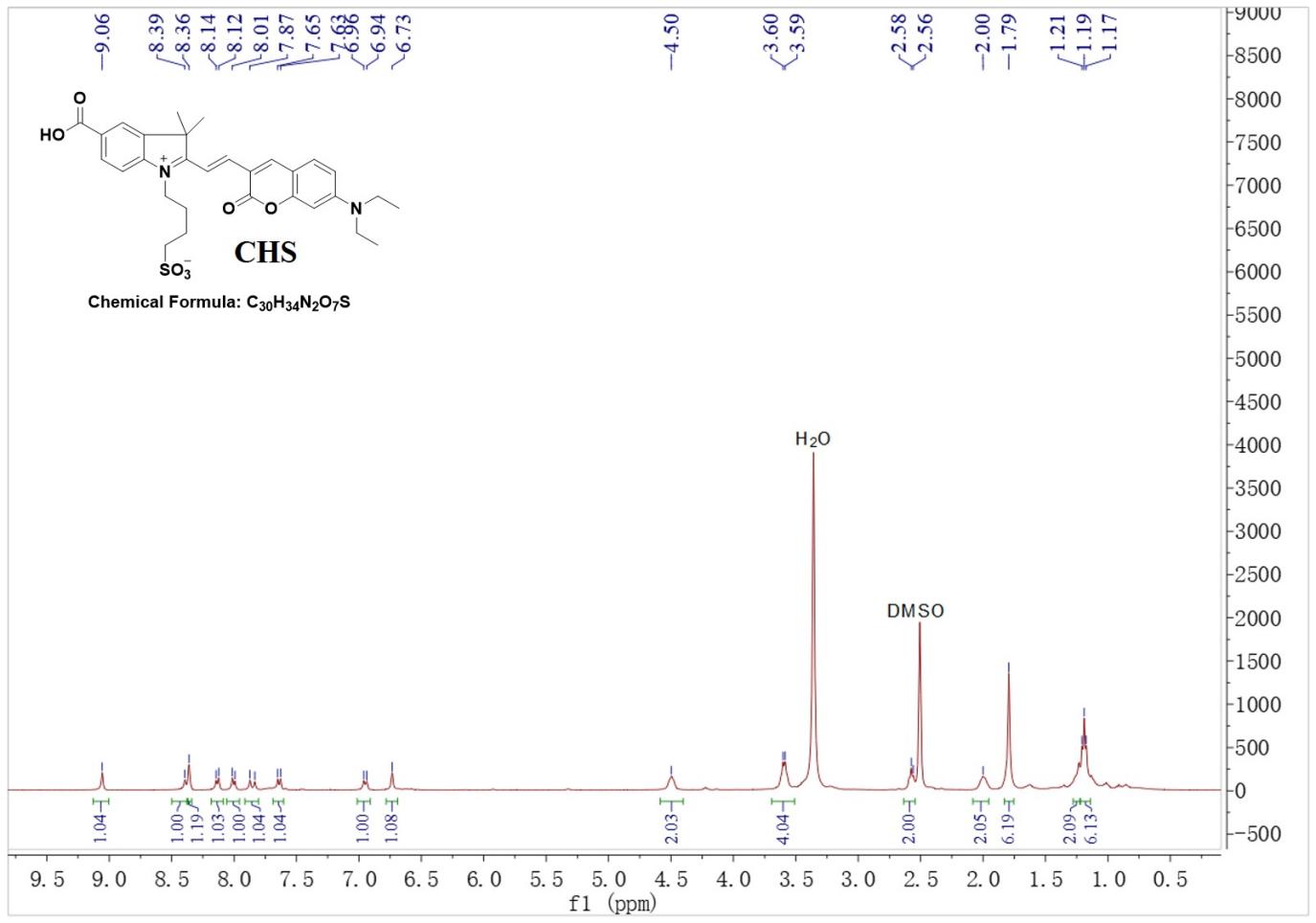


FIGURE S54 ^1^H NMR spectrum of CHS.


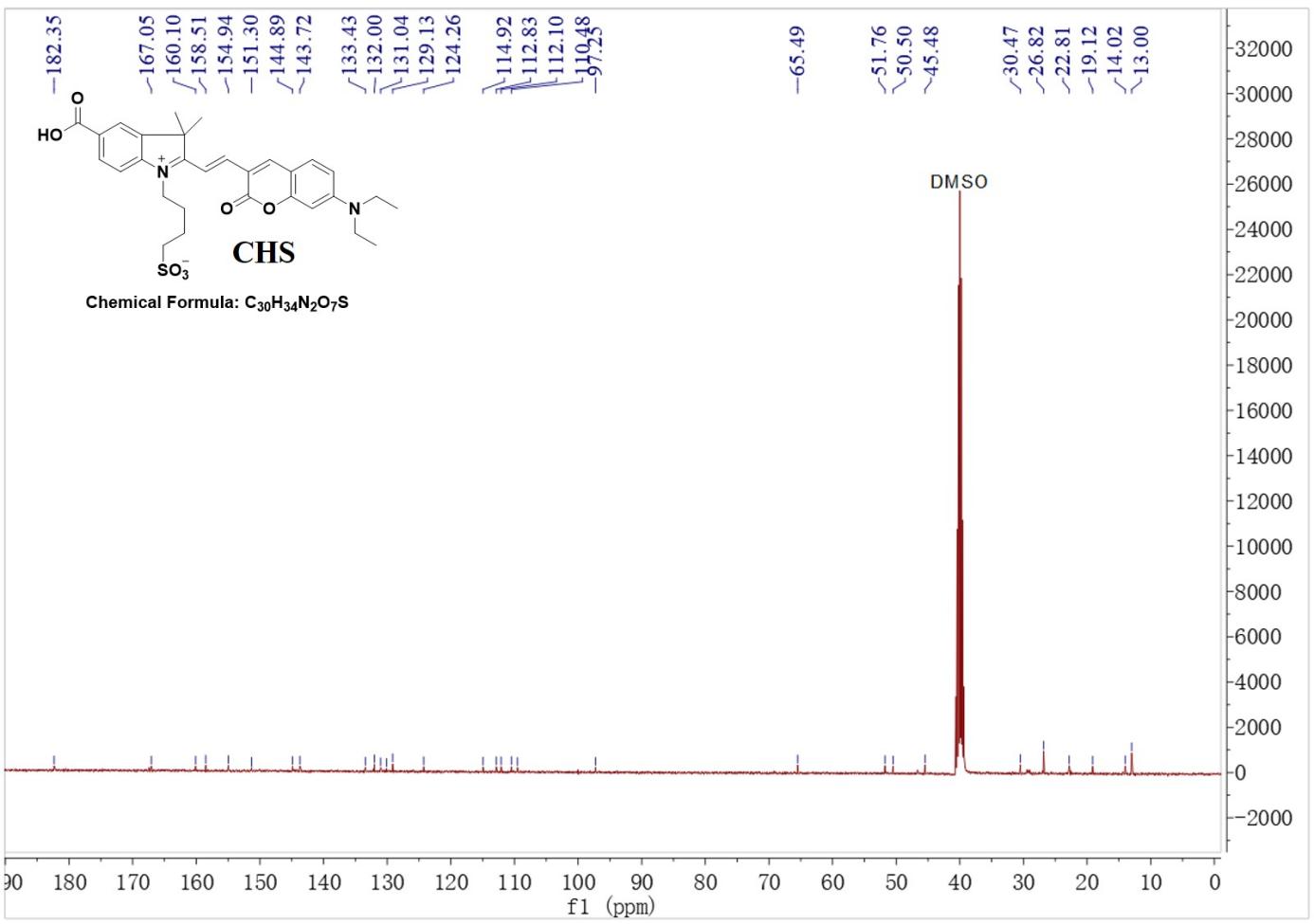


FIGURE S55 ^13^C NMR spectrum of CHS.

FIGURE S56 HRMS spectrum of CHS.

FIGURE S57 Synthesis of CPE2: the synthesis operation is the same as that of CPC. ^1^H NMR (600 MHz, DMSO-*d_6_*) *δ* (ppm) (Figure S25): 9.05 (d, *J* = 7.1 Hz, 2H), 8.84 (s, 1H), 8.39 (s, 1H), 8.32 (d, *J* = 7.1 Hz, 2H), 7.66 (d, *J* = 9.1 Hz, 1H), 6.88 (dd, *J* = 9.1, 2.4 Hz, 1H), 6.68 (d, *J* = 2.2 Hz, 1H), 4.62 (q, *J* = 7.3 Hz, 2H), 3.56 (q, *J* = 7.0 Hz, 4H), 1.55 (t, *J* = 7.3 Hz, 3H), 1.18 (t, *J* = 7.1 Hz, 6H). ^13^C NMR (151 MHz, DMSO-*d_6_*) *δ* (ppm) (Figure S26): 160.46, 158.10, 154.22, 150.01, 145.28, 144.98, 144.82, 132.97, 123.23 116.91, 111.58, 111.31, 108.85, 102.17, 97.29, 56.04, 45.28, 16.61, 12.94. HRMS (Figure S27): For [M]^+^ m/z 374.1863. Found: [M]^+^ m/z 374.1775.


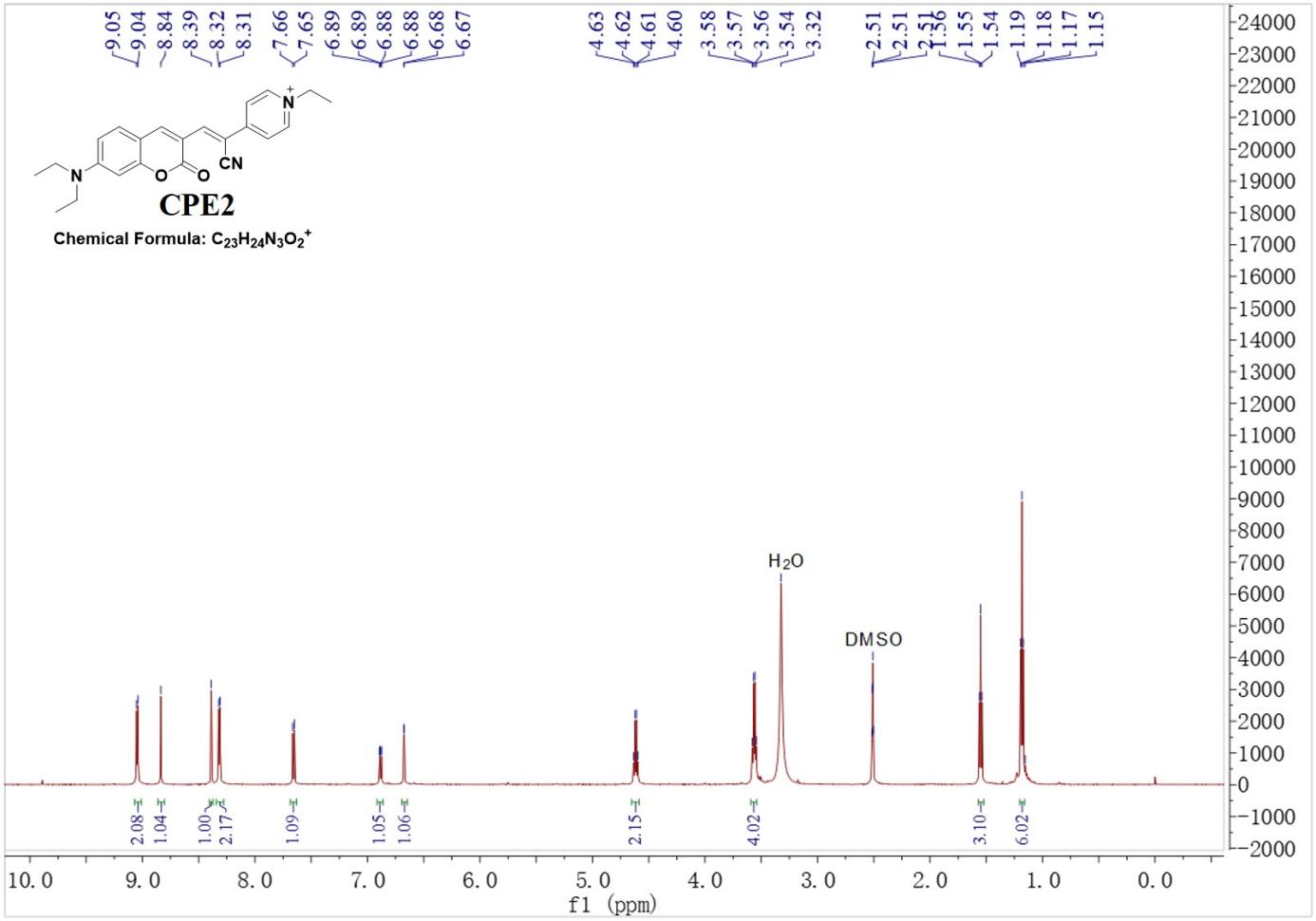


FIGURE S58 ^1^H NMR spectrum of CPE2.


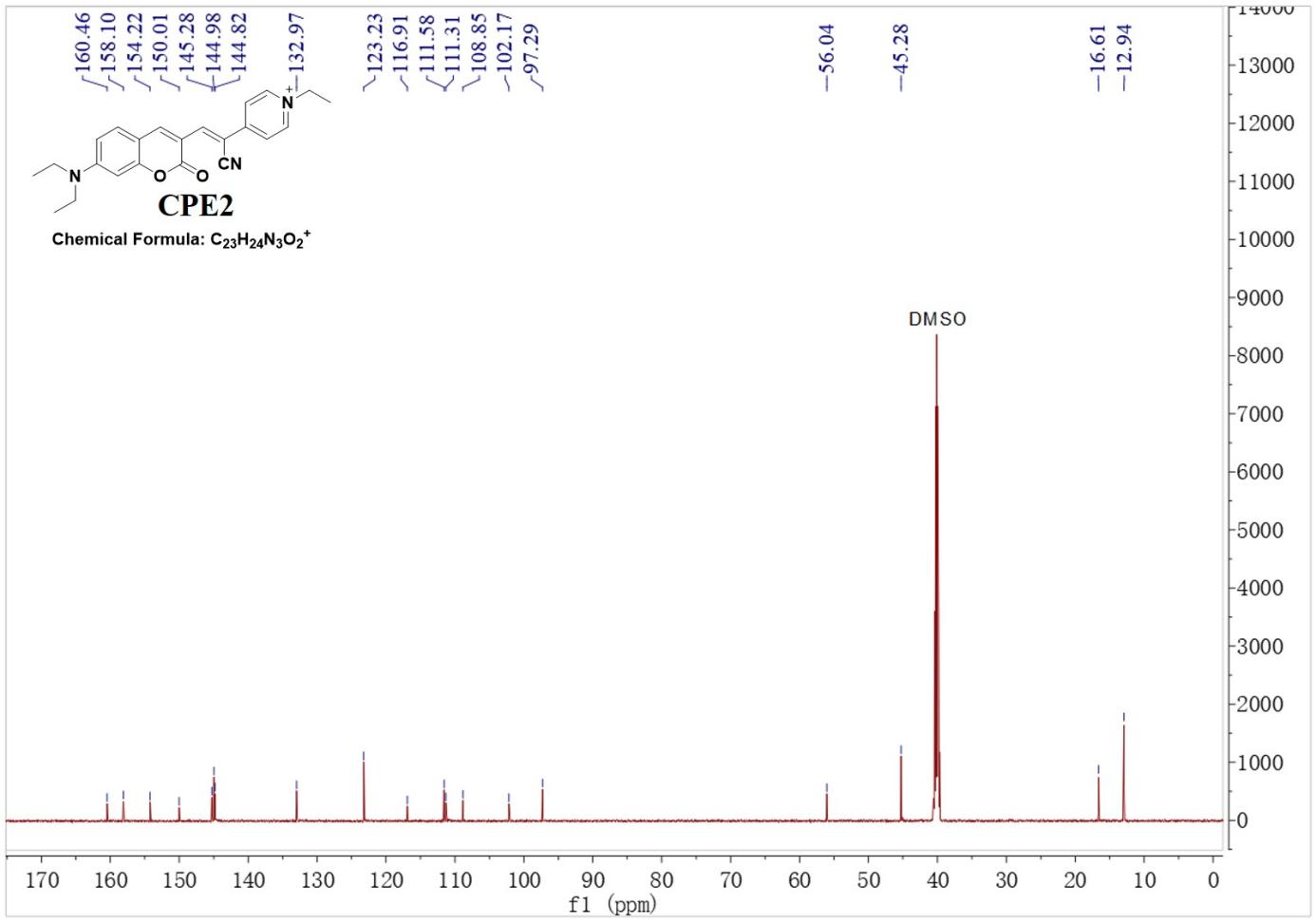


FIGURE S59 ^13^C NMR spectrum of CPE2.


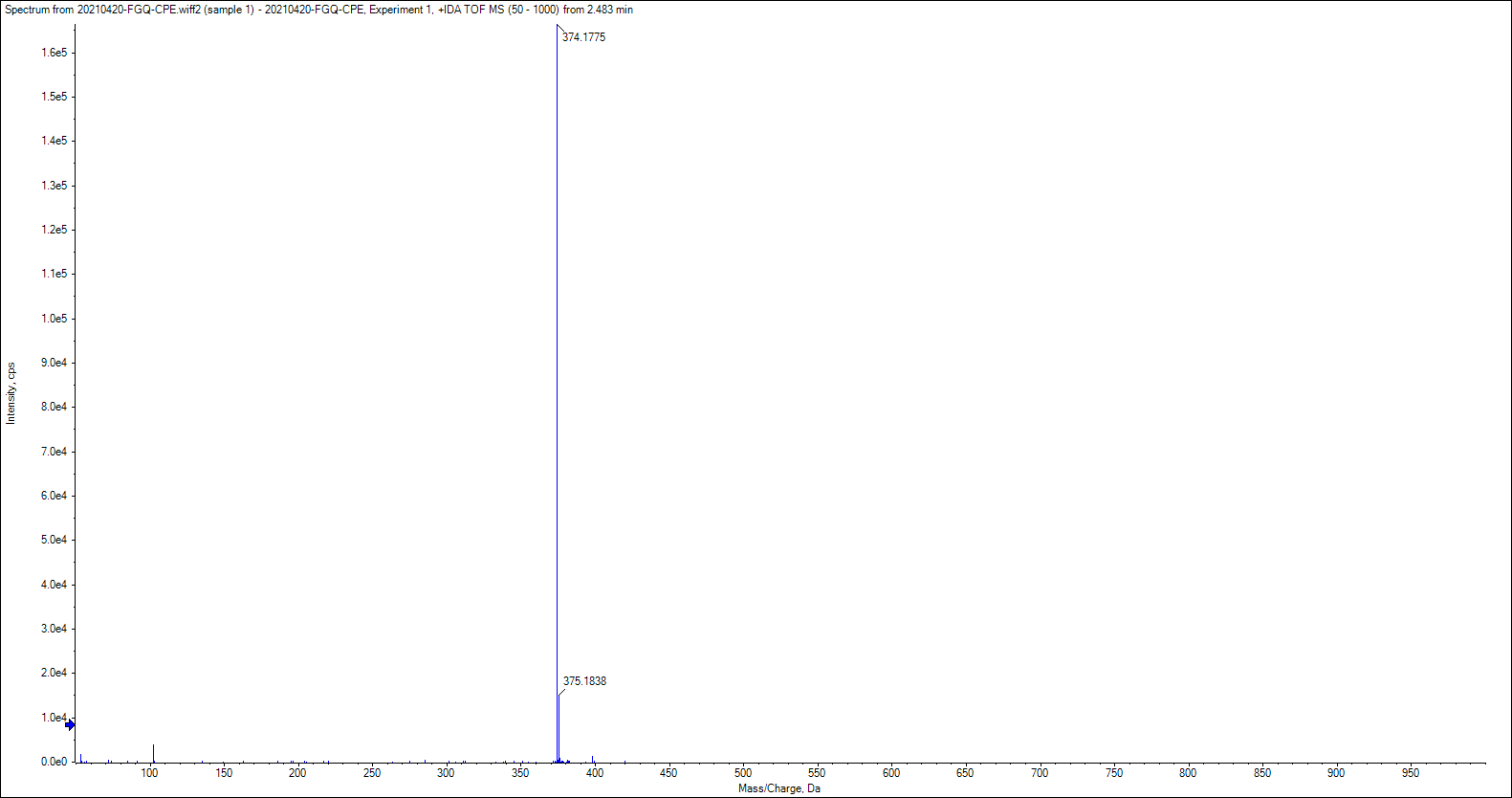


FIGURE S60 HRMS spectrum of CPE2.

FIGURE S61. Synthesis of NLC: the synthesis operation is the same as that of CPC. ^1^H NMR (600 MHz, DMSO-*d_6_*) *δ* (ppm) (Figure S28): 8.96 (d, *J* = 7.1 Hz, 2H), 8.83 (s, 1H), 8.43 (s, 1H), 8.37 (s, 1H), 8.31 (d, *J* = 7.2 Hz, 2H), 7.82 (dd, *J* = 7.3, 151 1.6 Hz, 1H), 7.65 (d, *J* = 9.1 Hz, 1H), 7.49 (td, *J* = 7.5, 1.6 Hz, 1H), 7.44 (td, *J* = 7.4, 152 1.3 Hz, 1H), 7.32 (d, *J* = 7.7 Hz, 1H), 6.88 (dd, *J* = 9.2, 2.4 Hz, 1H), 6.67 (d, *J* = 2.3 Hz, 1H), 6.01 (s, 2H), 3.56 (q, *J* = 7.0 Hz, 4H), 3.17 (s, 1H), 1.18 (t, *J* = 7.1 Hz, 6H). ^13^C NMR (151 MHz, DMSO-*d_6_*) *δ* (ppm) (Figure S29): 160.43, 158.11, 154.26, 150.27, 145.37, 145.28, 144.82, 138.22, 135.92, 133.00, 130.88, 130.03, 128.85, 123.12, 116.85, 111.59, 111.29, 108.89, 102.05, 97.31, 63.23, 49.05, 45.28, 12.94. HRMS (Figure S30): For [M]^+^ m/z 480.3505. Found: [M]^+^ m/z 480.2041.


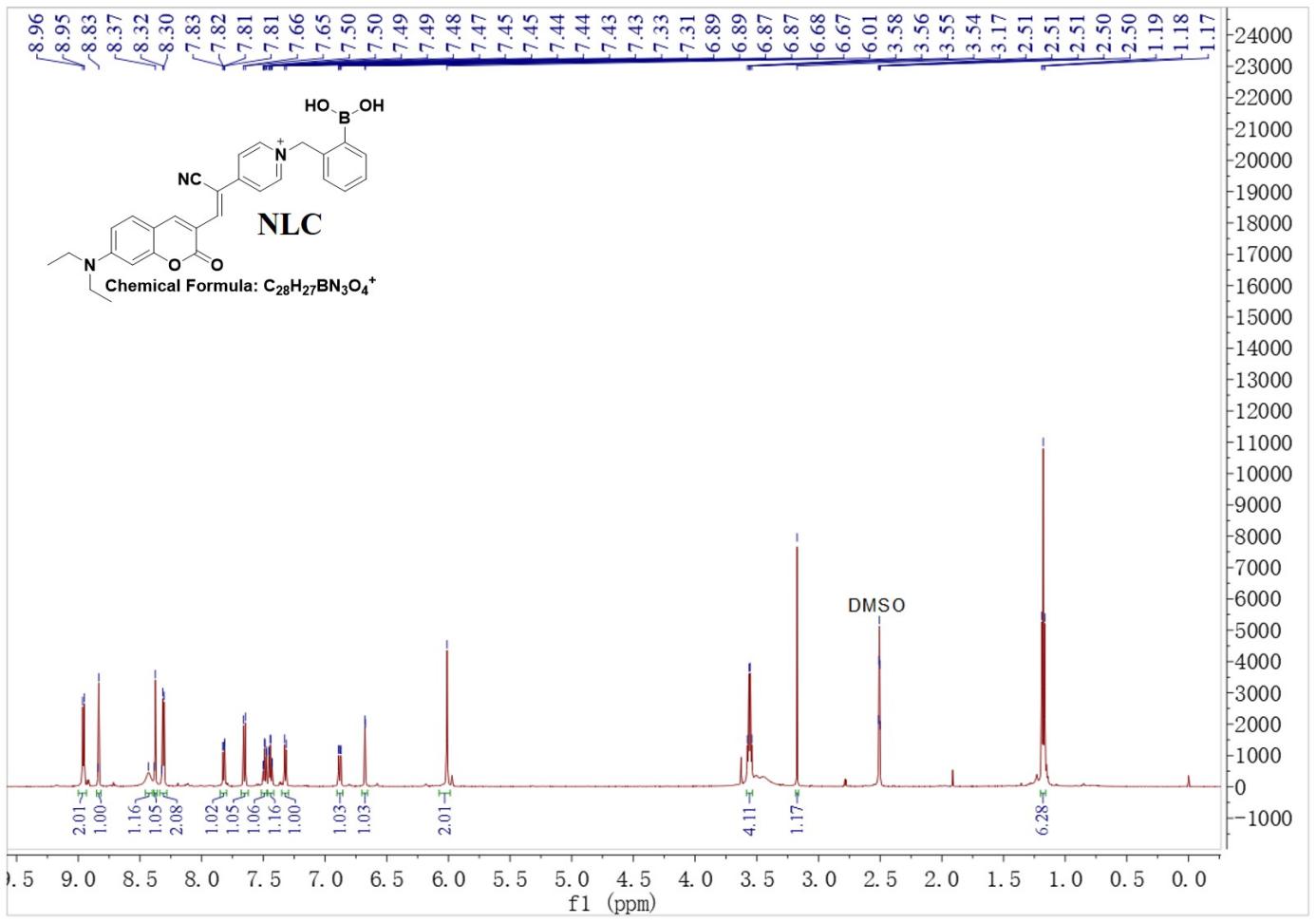


FIGURE S62 ^1^H NMR spectrum of NLC.


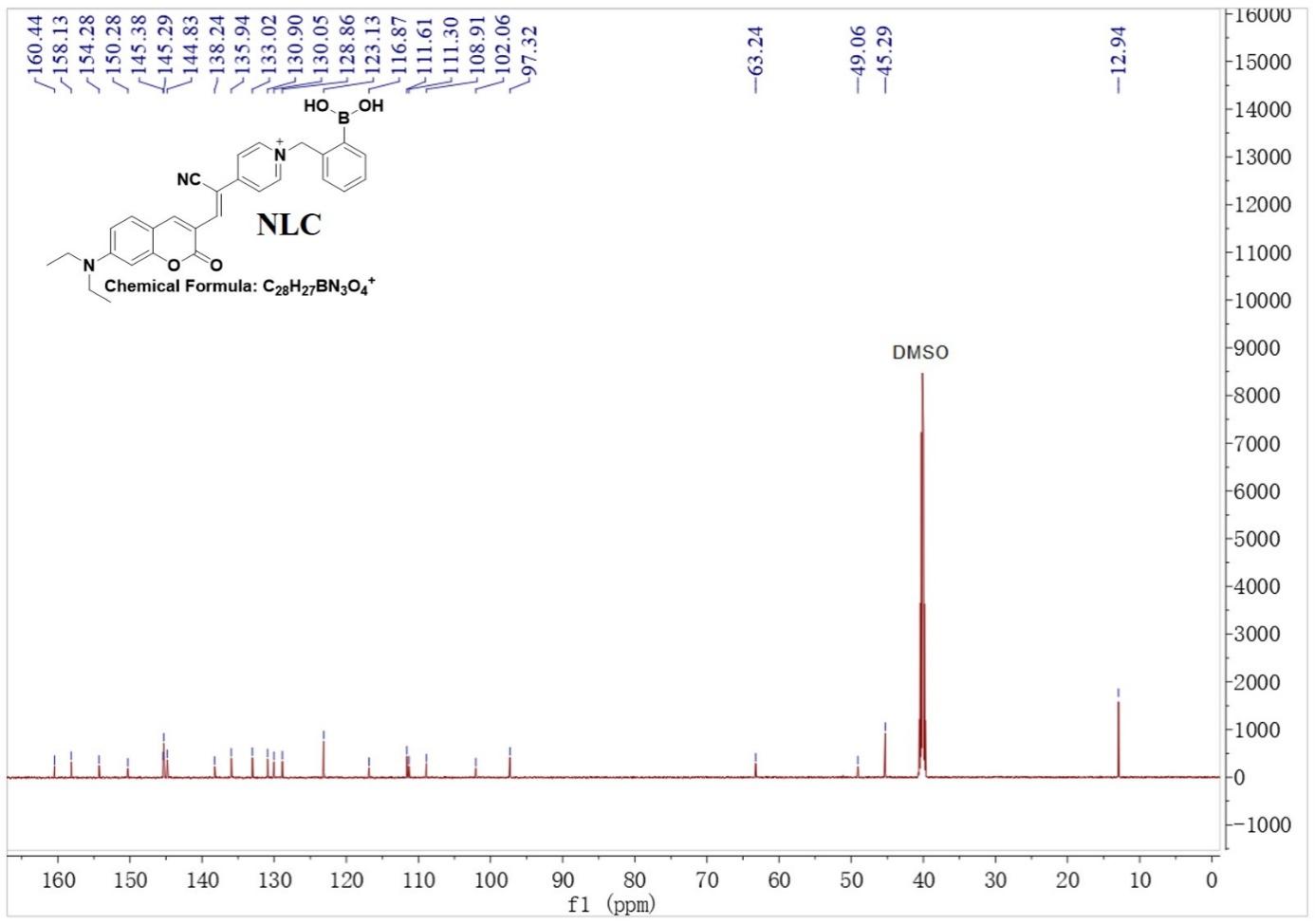


FIGURE S63 ^13^C NMR spectrum of NLC.

FIGURE S64 HRMS spectrum of NLC.

FIGURE S65 Synthesis of NDC: the synthesis operation is the same as that of CPC. ^1^H NMR (600 MHz, DMSO-*d_6_*) *δ* (ppm) (Figure S31): 9.15 (d, *J* = 6.6 Hz, 2H), 8.83 (s, 1H), 8.39 (s, 1H), 8.35 – 8.31 (m, 2H), 8.10 (s, 2H), 7.88 – 7.84 (m, 2H), 7.65 (d, *J* = 9.2 Hz, 1H), 7.48 (d, *J* = 7.8 Hz, 2H), 6.88 (dd, *J* = 9.2, 2.4 Hz, 1H), 6.67 (d, *J* = 2.2 Hz, 1H), 5.87 (s, 2H), 3.56 (q, *J* = 7.1 Hz, 4H), 1.18 (t, *J* = 7.1 Hz, 6H). ^13^C NMR (151 MHz, DMSO-*d_6_*) *δ* (ppm) (Figure S32): 160.40, 158.12, 154.29, 150.56, 145.50, 145.24, 144.86, 136.37, 135.30, 133.03, 128.01, 123.49, 116.86, 111.62, 111.27, 108.92, 101.99, 97.31, 62.91, 45.29, 25.11, 12.94. HRMS (Figure S33): For [M]^+^ m/z 480.3505. Found: [M]^+^ m/z 480.1216.


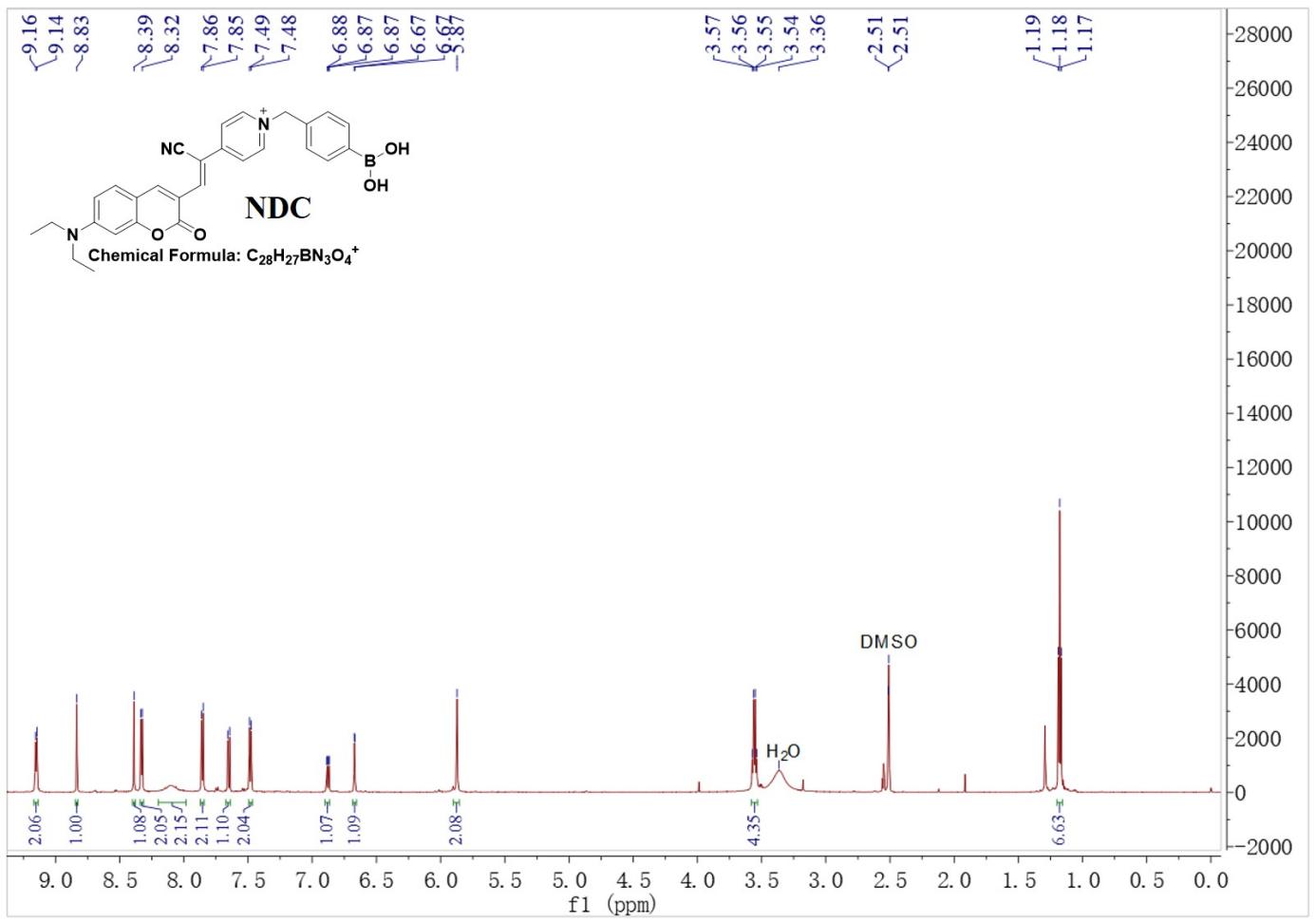


FIGURE S66 ^1^H NMR spectrum of NDC.


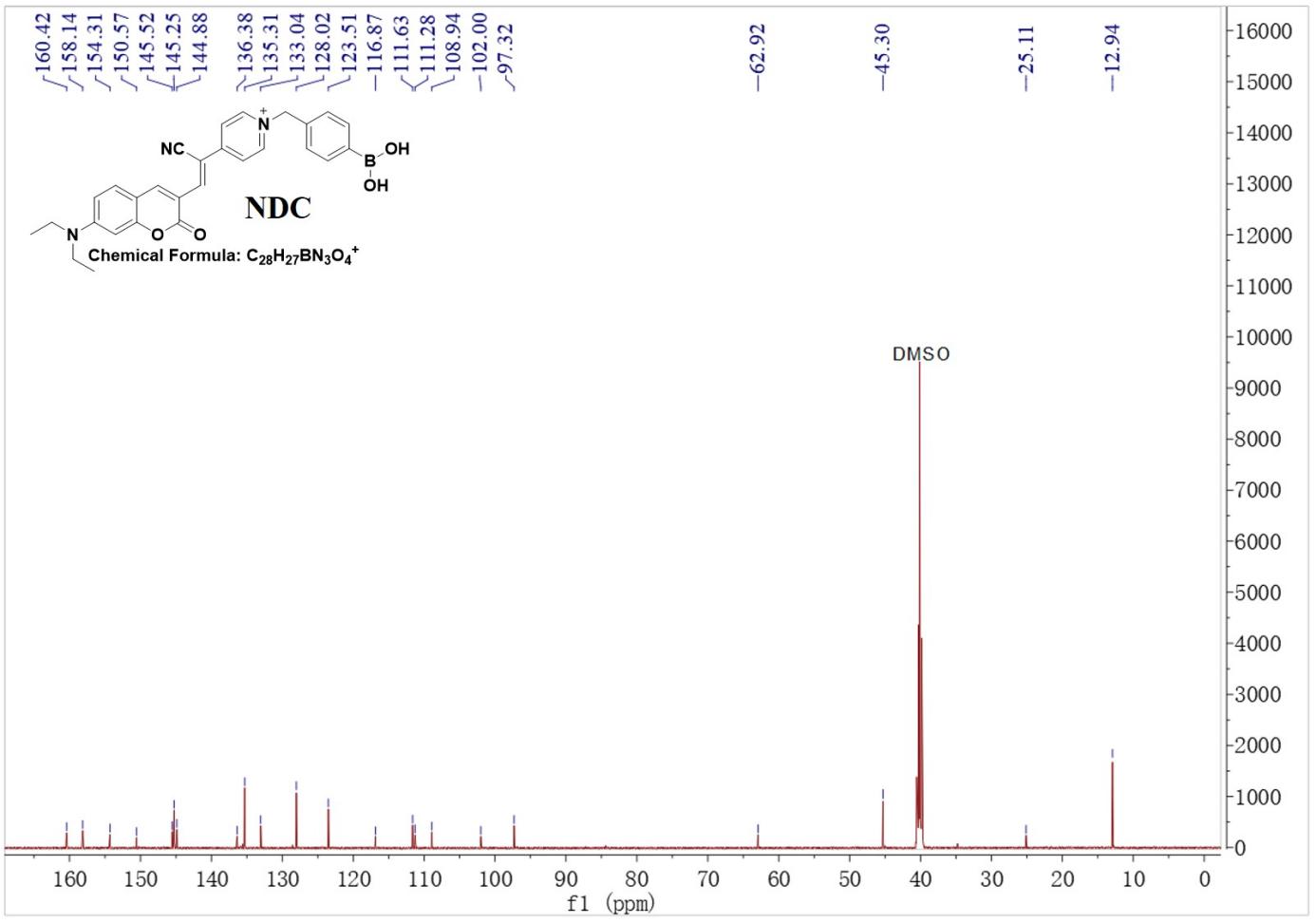


FIGURE S67 ^13^C NMR spectrum of NDC.

FIGURE S68 HRMS spectrum of NDC.

FIGURE S69 Synthesis of NJC: the synthesis operation is the same as that of CPC. ^1^H NMR (600 MHz, DMSO-*d_6_*) *δ* (ppm) (Figure S34): 9.16 (d, *J* = 7.1 Hz, 2H), 8.83 (s, 1H), 8.39 (s, 1H), 8.33 (d, *J* = 7.1 Hz, 2H), 8.10 (s, 2H), 7.89 (s, 1H), 7.84 (d, *J* = 7.4 Hz, 1H), 7.64 (d, *J* = 9.2 Hz, 1H), 7.58 (d, *J* = 7.7 Hz, 1H), 7.43 (t, *J* = 7.5 Hz, 1H), 6.87 (dd, *J* = 9.1, 2.3 Hz, 1H), 6.66 (d, *J* = 2.2 Hz, 1H), 5.88 (s, 2H), 3.33 (s, 4H), 1.18 (t, *J* = 7.1 Hz, 6H). ^13^C NMR (151 MHz, DMSO-*d_6_*) *δ* (ppm) (Figure S35):160.38, 158.12, 154.29, 150.50, 145.48, 145.22, 144.87, 135.34, 134.72, 133.79, 133.01, 130.78, 128.69, 123.48, 116.84, 111.61, 111.30, 108.92, 102.03, 97.31, 63.17, 45.29, 29.39, 12.93. HRMS (Figure S36): For [M]^+^ m/z 480.3505. Found: [M]^+^ m/z 480.1222.


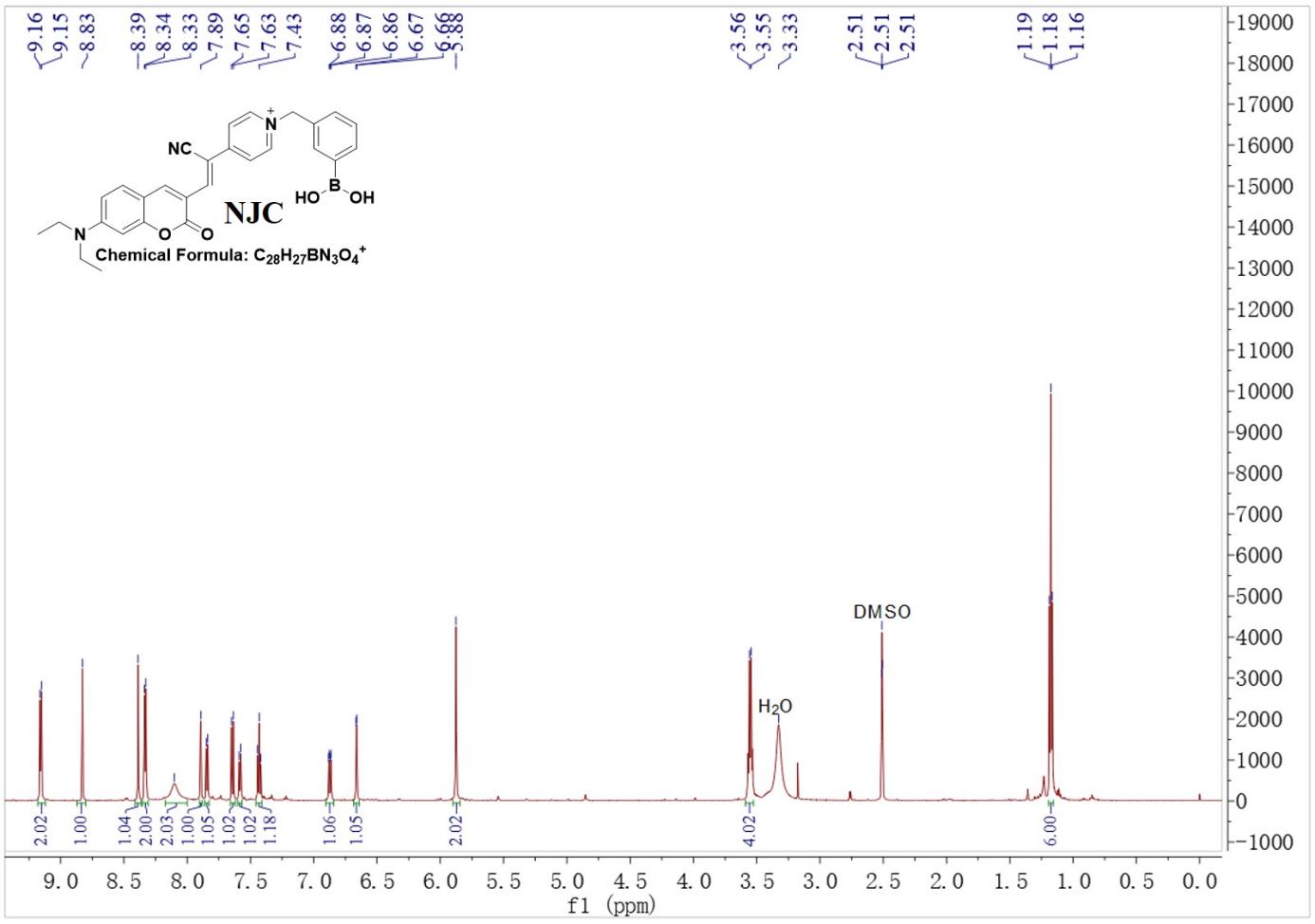


FIGURE S70 ^1^H NMR spectrum of NJC.


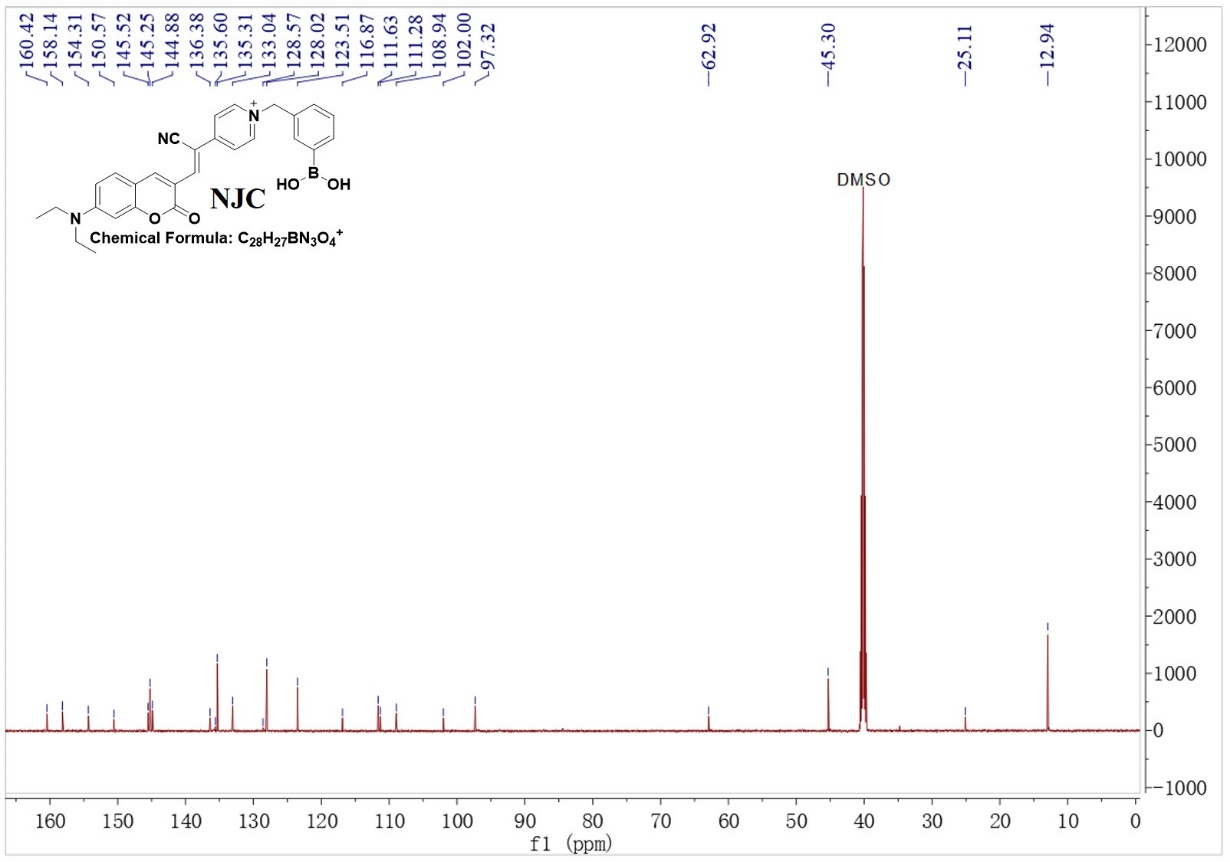


FIGURE S71 ^13^C NMR spectrum of NJC.

FIGURE S72 HRMS spectrum of NJC.

Table S1 Water-octanol partition coefficient of CPC.

|  | ***A_1_*** | ***C_1_*** | ***A_2_*** | ***C_2_*** | ***P*** | **Log*P*** | **Mean** | **Error** |
| --- | --- | --- | --- | --- | --- | --- | --- | --- |
| 1 | 0.212 | 18.115 | 0.636 | 57.447 | 3.171 | 0.501 | 0.482 | 0.02 |
| 2 | 0.227 | 19.506 | 0.657 | 59.395 | 3.045 | 0.484 |  |  |
| 3 | 0.243 | 20.991 | 0.670 | 60.601 | 2.887 | 0.460 |  |  |

*Notes:* ***A_1_*** and ***A_2_*** were the UV absorption values (458 nm) in the water phase and octanol phase, respectively; ***C_1_*** and ***C_2_*** were the concentration of **CPC** in the water phase and octanol phase, respectively (μM). ***C_1_*** and ***C_2_*** were calculated form the standard curve of **CPC** (*Y=*0.01078*X+*0.01672); ***P*** was calculated by [***C_2_***] / [***C_1_***].

**REFERENCES**

[1] Liu, Y.; Zhang, C.; Wei, Y.; Chen, H.; Kong, L.; Chen, Q.; Wang, Y., *Chem Eng J* **2021,** *422*, 130151.

[2] Carravilla, P.; Dasgupta, A.; Zhurgenbayeva, G.; Danylchuk, D. I.; Klymchenko, A. S.; Sezgin, E.; Eggeling, C., *Biophysical Reports* **2021,** *1* (2), 100023.

[3]Collot, M.; Pfister, S.; Klymchenko, A. S., *Current Opinion in Chemical Biology* **2022,** *69*, 102161.

[4]Chen, Q.; Jin, C.; Shao, X.; Guan, R.; Tian, Z.; Wang, C.; Liu, F.; Ling, P.; Guan, J. L.; Ji, L.; Wang, F.; Chao, H.; Diao, J., *Small* **2018,** *14* (41), e1802166.
